# Design, Synthesis, and Safener Activity of Novel Methyl (*R*)-*N*-Benzoyl/Dichloroacetyl-Thiazolidine-4-Carboxylates

**DOI:** 10.3390/molecules23010155

**Published:** 2018-01-12

**Authors:** Li-Xia Zhao, Hao Wu, Yue-Li Zou, Qing-Rui Wang, Ying Fu, Chun-Yan Li, Fei Ye

**Affiliations:** College of Science, Northeast Agricultural University, Harbin 150030, China; zhaolixia@neau.edu.cn (L.-X.Z.); wuhao2015ji@163.com (H.W.); zouyueli@163.com (Y.-L.Z.); qingruiwang@hotmail.com (Q.-R.W.); fuying@neau.edu.cn (Y.F.); chunyanli@neau.edu.cn (C.-Y.L.)

**Keywords:** methyl (*R*)-*N*-benzoyl/dichloroacetyl-thiazolidine-4-carboxylates, active substructure combination, safener activity

## Abstract

A series of novel methyl (*R*)-*N*-benzoyl/dichloroacetyl-thiazolidine-4-carboxylates were designed by active substructure combination. The title compounds were synthesized using a one-pot route from l-cysteine methyl ester hydrochloride, acyl chloride, and ketones. All compounds were characterized by IR, ^1^H NMR, ^13^C NMR, and HRMS. The structure of **4q** was determined by X-ray crystallography. The biological tests showed that the title compounds protected maize from chlorimuron-ethyl injury to some extent. The ALS activity assay showed that the title compounds increased the ALS activity of maize inhibited by chlorimuron-ethyl. Molecular docking modeling demonstrated that Compound **4e** competed against chlorimuron-ethyl to combine with the herbicide target enzyme active site, causing the herbicide to be ineffective.

## 1. Introduction

Acetolactate synthase (ALS) is an essential enzyme in the biosynthesis of branched-chain amino acids. It is the target of ALS-inhibiting herbicides, including imidazolinone, sulfonylurea, sulfonylamino-carbonyl-triazolinone, triazolopyrimidine, and pyrimidinyl-thiobenzoate herbicides [1]. ALS-inhibiting herbicides prevent the synthesis of isoleucine, leucine, and valine, resulting in subsequent weed death [2]. In addition, these herbicides have played a significant role in controlling weeds since the early 1980s due to their low toxicity, low cost, high activity, and safety [3]. As a type of ALS-inhibiting herbicide, chlorimuron-ethyl has been widely used to control a range of broadleaf weeds in farmland, especially in soybean fields over the past decade [4,5]. However, chlorimuron-ethyl treatments have been reported to cause phytotoxicity in beans, leading to reduced plant height and shoot dry matter [6]. Residues of this herbicide in soil may also inhibit the growth of succeeding crops, such as maize [7]. In order to reduce the injury, various methods have been reported, including restricted use of long residual herbicides, developing new herbicides and so on [8]. In addition to these methods, herbicide safeners, a class of agrochemicals that can reduce the negative effects of a herbicide on crops, have been commercialized [9,10].

**Scheme 1:**
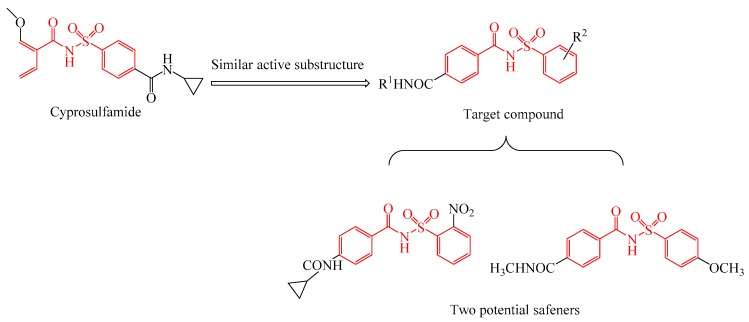
Design of new molecules as potential safeners.

Over the past decade, novel herbicide safeners have been synthesized using a structure-based bioisosterism design, which is a useful strategy for structural modification [11]. On the other hand, active substructure combinations have also proved to be very significant for synthesizing novel safeners, and these combinations can provide useful information about chemical substituents. Novel acylsulfamoylbenzamide safeners with excellent bioactivity have been designed using the previously developed safener cyprosulfamide, as the leading compounds based on similar active substructures (Scheme 1) [12]. Two compounds based on acylsulfamoylbenzamide have better bioactivities than cyprosulfamide and could serve as leading compounds in the design of new safeners. In addition, isoxadifen-ethyl was designed by combining the active substructures of known active molecules [13]. Furthermore, molecular docking has been performed to compare the binding affinities of herbicides and the target compound with the target enzyme (i.e., ALS) [14]. The binding energy was calculated, which helped researchers predict the mechanism of the herbicide safener [15].

**Scheme 2:**
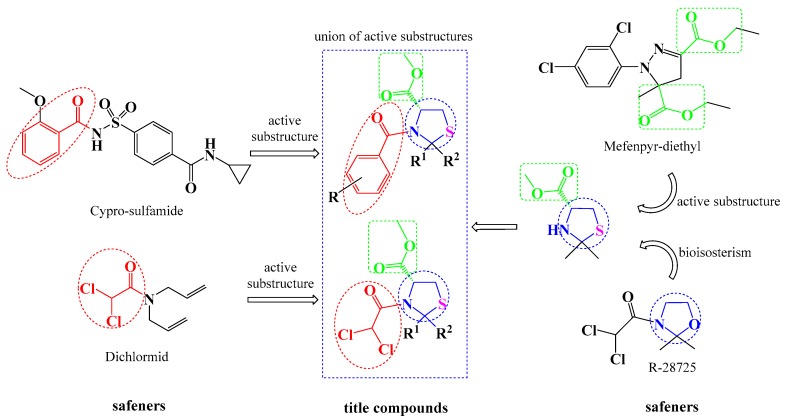
Design of the title compounds.

As a mature safener, R-28725 (2,2-dichloro-1-(2,2-dimethyloxazolidin-3-yl)ethan-1-one) shows good safener biological activity [9], and thiazolidine may possess similar chemical properties to R-28725 due to bioisosterism [16]. Recently, researchers reported some thiazolidine compounds with favorable biological activities in protecting the maize from herbicide phytotoxicity [11]. According to the facts mentioned above and continuing our previous research on the design of nitrogen-containing heterocyclic herbicide safeners [17], a series of thiazolidine-4-carboxylates combined with different groups at the *N*-3 position were designed and synthesized based on bioisosterism and active substructure combinations retaining the thiazolidine ring as the parent skeleton structure (Scheme 2).

**Scheme 3:**
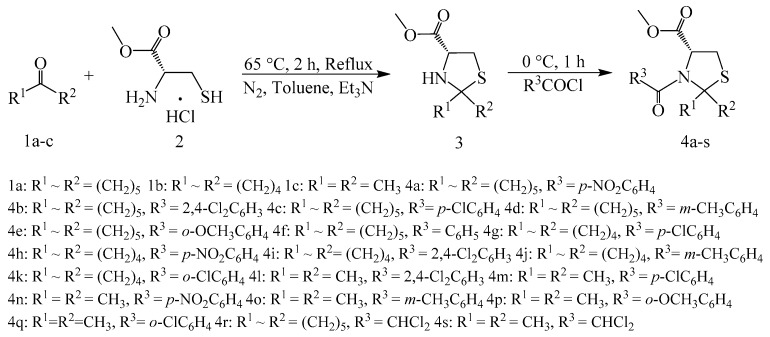
Route for the synthesis of the title compounds.

Due to the importance of thiazolidine derivatives, various synthetic routes to these compounds have been reported in many fields. The most frequently used method is the condensation of cysteine methyl ester hydrochloride with ketones in the presence of K_2_CO_3_ [18,19]. As for *N*-benzoylthiazolidines, they are usually prepared by the acylation of the corresponding thiazolidine compound, which is treated with an acyl chloride in the presence of the base [20]. However, these reactions, which involve cyclization and acylation, are time-consuming and complicated. According to our previous research [13], the final products were directly synthesized by a one-pot reaction (Scheme 3), representing an efficient and time saving route. The aim of this study was to synthesize new methyl (*R*)-*N*-benzoyl/dichloroacetyl-thiazolidine-4-carboxylates and determine their biological activities using biological tests. Molecular docking was also performed to research the mechanism of the safener.

## 2. Results and Discussion

### 2.1. Chemistry

In this paper, a one-pot synthesis route was designed (Scheme 3) and the effects of solvents and cyclization temperature were investigated. l-cysteine methyl ester hydrochloride, **2**, was cyclized with ketones, **1**, to generate thiazolidines, **3**, with Et_3_N as the attaching acid agent under nitrogen atmosphere. The title compounds, **4**, were prepared by direct acylation of the corresponding thiazolidine compound with an acyl chloride. Notably, use of toluene as the solvent provided better yields than CH_2_Cl_2_, THF, and CHCl_3_. In addition, the effect of temperature variation on the cyclization was determined by increasing the temperature from 25 to 75 °C. It was found that the best yields were obtained when controlling the cyclization temperature at 65 °C.

**Scheme 4:**
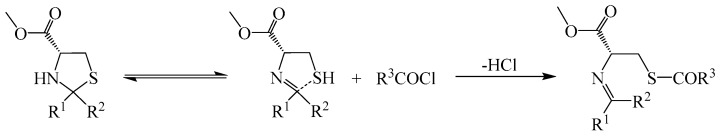
Mechanistic outline for the ring-opening reaction.

As shown in Table 1, the structure of ketone greatly affected the yield. When cyclohexanone was used, the formation of spiro compounds made the product more stable than other products. Thus, the yields of Compounds **4a**–**f** with spiro structures were higher than those of the other compounds, which were 69–91%. The yields were also considerably affected by the substituent structure on the benzene ring. For *p*-substituted phenyl with –NO_2_, the yields were significantly increased, especially the yield of Compound **4a**, which was increased by approximately 91%. Compounds **4d**, **4j**, and **4o** with Me-substituted phenyl were obtained in low yields; for example, the yield of Compound **4j** was decreased by 65%. Notably, the yields of Compounds **4r** and **4s**, with dichloromethyl substituents at R^3^, were among the lowest of all compounds, at 48% and 41%, respectively. Finally, side reactions affected the yield to some extent. These results were likely due to a chemical equilibrium between the thiazolidine and Schiff base [21], which resulted in the acyl chloride combining with the mercapto group, resulting in a decreased yield (Scheme 4).

The structures of all compounds, **4a**–**s**, were confirmed by ^1^H NMR, ^13^C NMR, and HRMS. All the compounds showed similar spectroscopic characteristics because of their structural similarity. In the IR spectra, two characteristic carbonyl bands at approximately 1630–1740 cm^−1^ proved the presence of the amide and ester groups. In the ^1^H NMR spectra of **4a**–**q**, the aromatic protons appeared in the region of 7.00–7.40 ppm, which also confirmed the successful acylation. The measured HRMS data also confirmed the proposed structures.

### 2.2. Crystal Structure of Compound ***4q***

**Figure 1:**
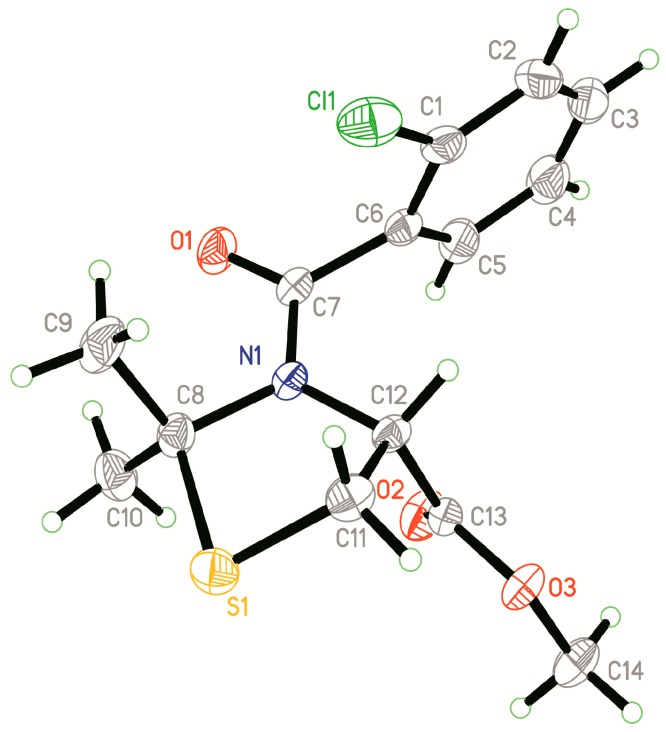
Molecular structure for Compound **4q** at 30% probability level.

**Figure 2:**
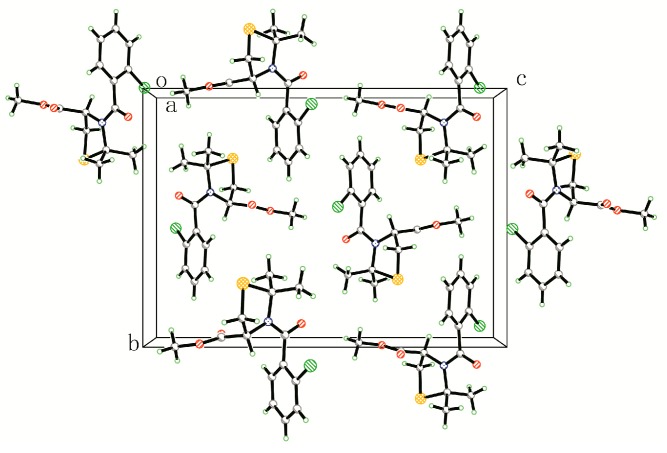
Packing view of Compound **4q**. The unit cell axes are labelled as o, a, b, c.

As shown in Figure 1, in the structure of methyl (*R*)-3-(*o*-chlorobenzoyl)-2,2-dimethylthiazolidine-4-carboxylate, the dihedral angle of the phenyl (C1/C2/C3/C4/C5/C6) and thiazolidine (N1/C8/S1/C11/C12) is 70.25°. The X-ray structure indicated that Compound **4q** contained a chiral carbon, C12, with R configuration. However, no obvious intermolecular hydrogen bonds and π–π interactions were observed in the structure of Compound **4q** (Figure 2).

### 2.3. Biological Activity Tests

According to our previous research on the biological activity of herbicide safeners [9], the residual concentration of chlorimuron-ethyl in soil was determined as 24 μg/kg. Before the tests, a preliminary screening was carried out to determine the best concentration of the title compounds, and Compound **4a** was selected for the preliminary screening. The best concentration of Compound **4a** was determined by varying the concentration from 5 to 100 mg/kg. It was found that the best growth index was obtained when controlling the concentration at 25 mg/kg. Thus, the best concentration was applied to the biological activity tests.

The protective effects of Compounds **4a**–**s** to maize from injury of chlorimuron-ethyl were evaluated, as shown in Table 2. Significant recovery of maize growth was observed when the title compounds were used as safeners and when the chlorimuron-ethyl concentration in soil was 24 μg/kg. All title compounds showed varying recovery rates for root length, root fresh weight, plant height, and plant fresh weight, indicating the successful design of the title compounds.

Comparing the protective effects of Compounds **4a**–**s** showed that substituents R^1^, R^2^, and R^3^ played a significant role in the safener activity of all title compounds. As shown in Table 2, biological activity tests revealed that Compounds **4a**–**k** with ring substituents at R^1^ and R^2^ displayed better safener activity than Compounds **4l**–**q** with Me substituents at R^1^ and R^2^. Similarly, Compound **4r** with ring substituents at R^1^ and R^2^ also showed increasing safener activity compared to Compound **4s** with Me substituents at R^1^ and R^2^. These results were likely due to the formation of spiro compounds, resulting in good safener activities. Furthermore, another crucial result was revealed regarding R^3^ substitution. For example, when substitution was introduced at different positions on the benzene ring, the title compounds showed varying recovery rates. Compound **4e** with methoxy at *o*-position on the benzene ring would show higher recovery rates for maize growth than Compounds **4a**, **4b**, **4c**, **4d**, and **4f**. Additionally, Compound **4c** with a chloro substituent at the *p*-position on the benzene ring also showed better safener activity than Compounds **4a**, **4b**, **4d**, and **4f**. Thus, it was found that the introduction of *o*-methoxybenzoyl and *p*-chlorobenzoyl at R^3^ did have an effect on safener activities. By comparison, Compounds **4c**, **4e**, and **4g** showed better recovery rates for maize growth than the other compounds. The protective effects of **4e** were superior to the effects of R-28725, which is a commercial safener and effectively reduced injury from chlorimuron-ethyl herbicides.

### 2.4. Effect of Safeners on ALS Activity

Chlorimuron-ethyl controls weeds by inhibiting ALS, which is important in the biosynthesis of the branched-chain amino acid. A direct assay on the ALS activity was carried out to confirm the positive effect of the title compounds on ALS activity. As shown in Table 3, ALS activity was expressed as the amount of acetylmethylcarbinol formed per hour per milligram protein. Comparing the ALS activity values of the control treatment and chlorimuron-ethyl treatment showed that chlorimuron-ethyl provoked an obvious decrease in the ALS activity; however, a significant increase was observed after the treatment of R-28725 and Compounds **4a**–**s**. The tested compounds showed a varying degree of positive effects with ALS activity values between 0.055 and 0.085 nmol h^−1^ mg^−1^ protein when compared with the chlorimuron-ethyl treatment (0.046 nmol h^−1^ mg^−1^ protein). In contrast to other tested compounds, Compounds **4c** and **4e** almost reversed the inhibition caused by chlorimuron-ethyl, which showed similar effects as R-28725. Among the series, Compound **4e** revealed the best effects with an ALS activity value of 0.85 nmol h^−1^ mg^−1^ protein. These data could indicate that the title compounds can increase ALS activity of maize inhibited by chlorimuron-ethyl significantly.

### 2.5. Molecular Docking Studies

**Figure 3:**
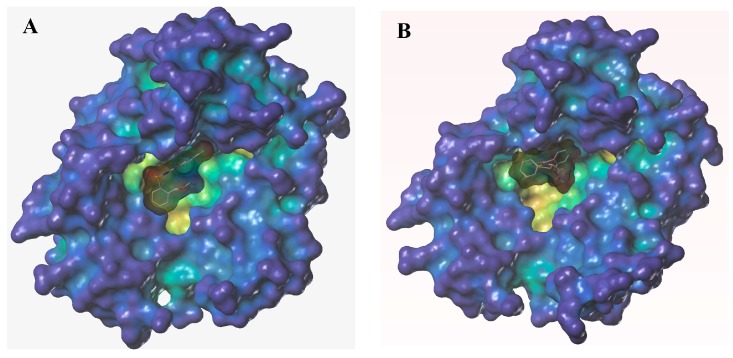
Docking modeling of chlorimuron-ethyl (**A**) and **4e** (**B**) with ALS.

ALS is a key enzyme in the biosynthesis of branched-chain amino acids, which is the target of chlorimuron-ethyl. The crystal structure of ALS was provided by the Protein Data Bank (PDB ID 1N0H). In molecular docking studies, the binding modes of chlorimuron-ethyl to ALS were clarified (Figure 3A). Compound **4e** was selected to dock with ALS due to its superior safener activity (Figure 3B). Obviously, both chlorimuron-ethyl and Compound **4e** bound to the target active site of ALS. Molecular docking revealed the mechanism of the herbicidal activity: chlorimuron-ethyl prevented the substrate from binding with the active pocket by clogging the entrance to the channel. In comparison, Compound **4e** sufficiently bound to the target active site and prevented the combination of chlorimuron-ethyl with the target active site. Additionally, Compound **4e** had a smaller structure than chlorimuron-ethyl, hardly blocking the entrance to the channel. Therefore, there was an increased opportunity for the small substrate to enter into the channel and catalyze the active site.

**Figure 4:**
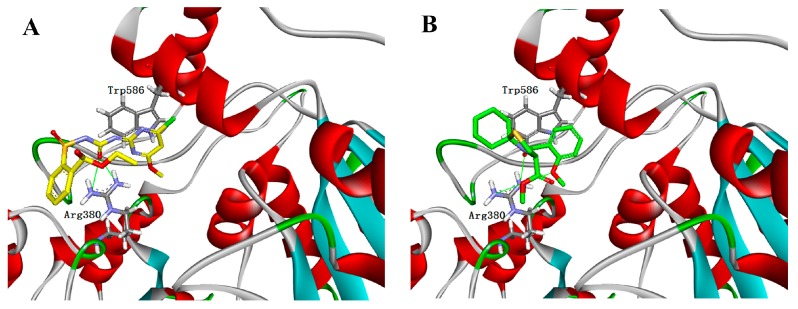
Zoomed-in view of the docking modeling of chlorimuron-ethyl (**A**) and **4e** (**B**). The carbon atoms are shown in yellow (**A**) and green (**B**); the sulfur atoms are shown in brown (**A**) and yellow (**B**); the oxygen atoms are shown in red (**A**,**B**); the nitrogen atoms are shown in blue (**A**,**B**), and the chlorine atoms are shown in green (**A**).

The docking modeling is revealed in greater detail in Figure 4. Chlorimuron-ethyl docked to ALS between the two active site residues (Arg 380 and Trp 586) and binding interactions included two hydrogen bonds between the oxygen atom and Arg 380. Compound **4e** also bound to the target site by hydrogen bonds. Obviously, three hydrogen bonds were formed between Compound **4e** and Arg 380, resulting in a stable combination. Upon application, **4e** possibly competed with chlorimuron-ethyl at the target active site by stopping the herbicide from acting on the ALS active pocket, which caused chlorimuron-ethyl to lose its effect. This function may be the detoxification mechanism of the title compound.

## 3. Materials and Methods

### 3.1. Reagents and Analysis

All the reagents were of analytical grade and used without further purification. Melting points were determined on a Beijing Taike melting point apparatus (X-4) (Taike, Beijing, China) and were uncorrected. IR spectra were obtained on a Bruker ALPHA-T spectrometer (BRUKER Inc., Beijing, China). ^1^H NMR and ^13^C NMR spectra were recorded on a Bruker AV400 spectrometer (BRUKER Inc., Beijing, China) with CDCl_3_ (Energy Chemical., Shanghai, China) as the solvent and TMS (Energy Chemical., Shanghai, China) as the internal standard. HRMS spectra were recorded on an FTICR-MS spectrometer (BRUKER Inc.). X-ray diffraction data were recorded on a BRUKER D8 VENTURE X-diffractometer (BRUKER Inc.) with Mo K*α* radiation (*λ* = 0.71073 Å) at 273(2) K.

### 3.2. General Procedure for the Preparation of ***4***

The synthetic route of the title compound **4**, is shown in Scheme 3. l-cysteine methyl ester hydrochloride (4.29 g, 25 mmol), an appropriate ketone (25 mmol), and Et_3_N (5.05 g, 50 mmol) were stirred for 2 h in toluene (20 mL) at 65 °C under nitrogen atmosphere. Benzoyl chloride or dichloroacetyl chloride (25 mmol) was added dropwise to the reaction mixture at 0 °C and reacted for 1 h. The mixture was washed with saturated NaCl solution (3 × 20 mL) and dried using anhydrous sodium sulfate. The solvent was removed under reduced pressure to yield crude methyl (*R*)-*N*-benzoyl/dichloroacetyl-thiazolidine-4-carboxylate (**4**). The title compounds were purified by column chromatography. The spectra data of compound **4** are presented in Supplementary Materials (Figures S2–S77).

*Methyl (R)-4-(p-nitrobenzoyl)-1-thia-4-azaspiro[4.5]decane-3-carboxylate* (**4a**). Yellow solid, m.p. 148 °C. Yield 91%. IR (KBr): *ν* (cm^−1^) 2914 (C-H), 1727, 1642 (C=O), 1515 (C=C); ^1^H NMR (400 MHz, CDCl_3_): *δ* (ppm) 8.26 (d, *J* = 8.8 Hz, 2H, Ar-H), 7.52 (d, *J* = 8.8 Hz, 2H, Ar-H), 4.66 (br, 1H, N-CH), 3.72 (s, 3H, O-CH_3_), 3.19–3.20 (m, 2H, S-CH_2_), 1.34–3.18 (m, 10H, C_6_H_10_); ^13^C NMR (100 MHz, CDCl_3_): *δ* (ppm) 170.38, 167.47, 147.98, 144.59, 126.85, 124.08, 81.14, 67.34, 53.06, 35.77, 35.18, 30.75, 25.60, 25.36, 24.49; HRMS calcd. for [M + Na^+^] C_17_H_20_N_2_O_5_S: 387.0985, found 387.0990.

*Methyl (R)-4-(2,4-dichlorobenzoyl)-1-thia-4-azaspiro[4.5]decane-3-carboxylate* (**4b**). White solid, m.p. 151–152 °C. Yield 81%. IR (KBr): *ν* (cm^−1^) 2843–2931 (C-H), 1734, 1633 (C=O), 1437–1575 (C=C); ^1^H NMR (400 MHz, CDCl_3_): *δ* (ppm) 7.26–7.40 (m, 3H, Ar-H), 4.50 (dd, *J* = 4.9, 1.7 Hz, 1H, N-CH), 3.71 (s, 3H, O-CH_3_), 3.23–3.24 (m, 2H, S-CH_2_), 1.31–3.23 (m, 10H, C_6_H_10_); ^13^C NMR (100 MHz, CDCl_3_): *δ* (ppm) 170.42, 164.97, 136.42, 135.47, 129.91, 129.29, 129.20, 127.68, 81.09, 67.18, 52.91, 35.99, 34.27, 31.03, 25.51, 25.37, 24.54; HRMS calcd. for [M + Na^+^] C_17_H_19_NO_3_SCl_2_: 410.0355, found 410.0359.

*Methyl (R)-4-(p-chlorobenzoyl)-1-thia-4-azaspiro[4.5]decane-3-carboxylate* (**4c**). White solid, m.p. 96–97 °C. Yield 77%. IR (KBr): *ν* (cm^−1^) 2937 (C-H), 1747, 1639 (C=O), 1442–1550 (C=C); ^1^H NMR (400 MHz, CDCl_3_): *δ* (ppm) 7.26–7.37 (m, 4H, Ar-H), 4.76 (d, *J* = 2.1 Hz, 1H, N-CH), 3.70 (s, 3H, O-CH_3_), 3.13–3.17 (m, 2H, S-CH2), 1.28–3.12 (m, 10H, C_6_H_10_); ^13^C NMR (100 MHz, CDCl_3_): *δ* (ppm) 170.78, 168.66, 137.45, 135.25, 128.90, 127.18, 80.86, 67.59, 52.87, 35.84, 35.21, 30.69, 25.67, 25.38, 24.53; HRMS calcd. for [M + H^+^] C_17_H_20_NO_3_SCl: 354.0925, found 354.0929.

*Methyl (R)-4-(m-methylbenzoyl)-1-thia-4-azaspiro[4.5]decane-3-carboxylate* (**4d**). White solid, m.p. 74–75 °C. Yield 69%. IR (KBr): *ν* (cm^−1^) 2834–2951 (C-H), 1737, 1637 (C=O), 1436 (C=C); ^1^H NMR (400 MHz, CDCl_3_): *δ* (ppm) 7.09–7.28 (m, 4H, Ar-H), 4.81 (d, *J* = 3.9 Hz, 1H, N-CH), 3.70 (s, 3H, O-CH_3_), 3.15–3.20 (m, 2H, S-CH_2_), 2.36 (s, 3H, CH_3_), 3.12–3.13, 1.32–1.86 (m, 10H, C_6_H_10_); ^13^C NMR (100 MHz, CDCl_3_): *δ* (ppm) 171.08, 169.85, 139.15, 138.47, 129.92, 128.51, 126.10, 122.48, 80.71, 67.70, 52.70, 35.91, 35.19, 30.74, 25.71, 25.44, 24.56, 21.38; HRMS calcd. for [M + Na^+^] C_18_H_23_NO_3_S: 356.1291, found 356.1289.

*Methyl (R)-4-(o-methoxybenzoyl)-1-thia-4-azaspiro[4.5]decane-3-carboxylate* (**4e**). White solid, m.p. 95–96 °C. Yield 71%. IR (KBr): *ν* (cm^−1^) 2851–2935 (C-H), 1744, 1642 (C=O), 1489 (C=C); ^1^H NMR (400 MHz, CDCl_3_): *δ* 7.24–7.33, 6.87–6.96 (m, 4H, Ar-H), 4.63 (d, *J* = 4.6 Hz, N-CH), 3.84 (s, 3H, O-CH_3_), 3.65 (s, 3H, O-CH_3_), 3.17–3.17 (m, 2H, S-CH_2_), 3.14–3.16, 1.31–1.85 (m, 10H, C_6_H_10_); ^13^C NMR (100 MHz, CDCl_3_): *δ* (ppm) 171.13, 167.07, 153.93, 130.35, 128.58, 128.16, 121.03, 110.94, 80.78, 67.17, 55.82, 52.56, 36.08, 34.50, 30.98, 25.59, 25.52, 24.63; HRMS calcd. for [M + H^+^] C_18_H_23_NO_4_S: 350.1421, found 350.1417.

*Methyl (R)-4-benzoyl-1-thia-4-azaspiro[4.5]decane-3-carboxylate* (**4f**). White solid, m.p. 98–99 °C. Yield 75%. IR (KBr): *ν* (cm^−1^) 2857–2941 (C-H), 1746, 1638 (C=O), 1442 (C=C); ^1^H NMR (400 MHz, CDCl_3_): *δ* (ppm) 7.31–7.40 (m, 5H, Ar-H), 4.81 (d, *J* = 3.4 Hz, 1H, N-CH), 3.69 (s, 3H, O-CH_3_), 3.15–3.17 (m, 2H, S-CH_2_), 3.13–3.14, 1.32–1.86 (m, 10H, C_6_H_10_); ^13^C NMR (100 MHz, CDCl_3_): *δ* (ppm) 170.00, 169.72, 139.13, 129.24, 128.62, 125.58, 80.75, 67.67, 52.74, 35.92, 35.23, 30.72, 25.71, 25.42, 24.57; HRMS calcd. for [M + Na^+^] C_17_H_21_NO_3_S: 342.1134, found 342.1140.

*Methyl (R)-4-(p-chlorobenzoyl)-1-thia-4-azaspiro[4.4]nonane-3-carboxylate* (**4g**). White solid, m.p. 101 °C. Yield 67%. IR (KBr): *ν* (cm^−1^) 2844–2931 (C-H), 1728,1637 (C=O), 1372 (C=C); ^1^H NMR (400 MHz, CDCl_3_): *δ* (ppm) 7.28–7.38 (m, 4H, Ar-H), 4.73 (dd, *J* = 5.7, 3.2 Hz, 1H, N-CH), 3.67 (s, 3H, O-CH_3_), 3.21–3.25 (m, 2H, S-CH_2_), 1.68–3.06 (m, 8H, C_5_H_8_); ^13^C NMR (100 MHz, CDCl_3_): *δ* (ppm) 170.68, 168.12, 136.88, 135.52, 128.86, 127.49, 82.21, 67.05, 52.85, 39.29, 38.11, 32.29, 25.16, 25.11; HRMS calcd. for [M + Na^+^] C_16_H_18_ClNO_3_S: 362.0588, found 362.0592.

*Methyl (R)-4-(p-nitrobenzoyl)-1-thia-4-azaspiro[4.4]nonane-3-carboxylate* (**4h**). Yellow solid, m.p. 141 °C. Yield 83%. IR (KBr): *ν* (cm^−1^) 2842–3079 (C-H), 1723, 1639 (C=O), 1521 (C=C); ^1^H NMR (400 MHz, CDCl_3_): *δ* (ppm) 8.26 (d, *J* = 8.7 Hz, 2H, Ar-H), 7.54 (d, *J* = 8.7 Hz, 2H, Ar-H), 4.63 (dd, *J* = 4.7, 3.0 Hz, 1H, N-CH), 3.69 (s, 3H, O-CH_3_), 3.25–3.28 (m, 2H, S-CH_2_), 1.61–3.08 (m, 8H, C_5_H_8_); ^13^C NMR (100 MHz, CDCl_3_): *δ* (ppm) 170.28, 166.90, 148.09, 144.15, 127.12, 124.02, 82.31, 66.80, 53.09, 39.56, 37.88, 32.33, 25.19, 25.04; HRMS calcd. for [M + Na^+^] C_16_H_18_N_2_O_5_S:373.0829, found 373.0832.

*Methyl (R)-4-(2,4-dichlorobenzoyl)-1-thia-4-azaspiro[4.4]nonane-3-carboxylate* (**4i**). White solid, m.p. 95 °C. Yield 72%. IR (KBr): *ν* (cm^−1^) 2842–2989 (C-H), 1733, 1632 (C=O), 1437–1574 (C=C); ^1^H NMR (400 MHz, CDCl_3_): *δ* (ppm) 7.26–7.40 (m, 3H, Ar-H), 4.47 (t, *J* = 3.5 Hz, 1H, N-CH), 3.70 (s, 3H, O-CH_3_), 3.27–3.28 (m, 2H, S-CH_2_), 2.93–3.13, 1.66–1.96 (m, 8H, C_5_H_8_); ^13^C NMR (100 MHz, CDCl_3_): *δ* (ppm) 170.27, 164.51, 136.01, 135.63, 130.06, 129.40, 129.29, 127.66, 82.02, 66.52, 52.92, 39.49, 37.42, 32.51, 25.31, 24.94; HRMS calcd. for [M + Na^+^] C_16_H_17_Cl_2_NO_3_S: 396.0198, found 396.0201.

*Methyl (R)-4-(m-methylbenzoyl)-1-thia-4-azaspiro[4.4]nonane-3-carboxylate* (**4j**). White solid, m.p. 79 °C. Yield 65%. IR (KBr): *ν* (cm^−1^) 2846–2984 (C-H), 1727, 1626 (C=O), 1407 (C=C); ^1^H NMR (400 MHz, CDCl_3_): *δ* (ppm) 7.11–7.27 (m, 4H, Ar-H), 4.78 (dd, *J* = 5.7, 2.9 Hz, 1H, N-CH), 3.66 (s, 3H, O-CH_3_), 3.17–3.24 (m, 2H, S-CH_2_), 2.36 (s, 3H, CH_3_), 2.95–3.08, 1.69–1.93 (m, 8H, C_5_H_8_); ^13^C NMR (100 MHz, CDCl_3_): *δ* (ppm) 170.98, 169.33, 138.52, 138.42, 130.17, 128.46, 126.42, 122.80, 82.09, 67.16, 52.69, 39.34, 38.11, 32.30, 25.16, 25.08, 21.36. HRMS calcd. for [M + Na^+^] C_17_H_21_NO_3_S: 342.1134, found 342.1140.

*Methyl (R)-4-(o-chlorobenzoyl)-1-thia-4-azaspiro[4.4]nonane-3-carboxylate* (**4k**). White solid, m.p. 111–112 °C. Yield 70%. IR (KBr): *ν* (cm^−1^) 2844–2936 (C-H), 1727, 1628 (C=O), 1423 (C=C); ^1^H NMR (400 MHz, CDCl_3_): *δ* (ppm) 7.28–7.38 (m, 4H, Ar-H), 4.50 (dd, *J* = 5.0, 1.8 Hz, 1H, N-CH), 3.69 (s, 3H, O-CH_3_), 3.20–3.32 (m, 2H, S-CH_2_), 3.19–3.20, 1.69–2.11 (m, 8H, C_5_H_8_); ^13^C NMR (100 MHz, CDCl_3_): *δ* (ppm) 170.50, 165.39, 137.56, 130.33, 129.39, 129.12, 128.34, 127.19, 82.01, 66.59, 52.78, 39.45, 37.47, 32.59, 25.32, 24.95; HRMS calcd. for [M + Na^+^] C_16_H_18_ClNO_3_S: 362.0588, found 362.0593.

*Methyl (R)-3-(2,4-dichlorobenzoyl)-2,2-dimethylthiazolidine-4-carboxylate* (**4l**). White solid, m.p. 67–68 °C. Yield 78%. IR (KBr): *ν* (cm^−1^) 2906–3066 (C-H), 1723, 1643 (C=O), 1573 (C=C); ^1^H NMR (400 MHz, CDCl_3_): *δ* (ppm) 7.26–7.40 (m, 3H, Ar-H), 4.47 (d, *J* = 5.1 Hz, 1H, N-CH), 3.71 (s, 3H, O-CH_3_), 3.25–3.38 (m, 2H, S-CH_2_), 2.03 (d, *J* = 14.7 Hz, 6H, C-(CH_3_)_2_); ^13^C NMR (100 MHz, CDCl_3_): *δ* (ppm) 170.23, 164.86, 135.92, 135.61, 130.02, 129.36, 129.27, 127.67, 73.54, 67.16, 52.94, 31.61, 29.00, 27.77; HRMS calcd. for [M + Na^+^] C_14_H_15_Cl_2_NO_3_S: 370.0042, found 370.0046.

*Methyl (R)-3-(p-chlorobenzoyl)-2,2-dimethylthiazolidine-4-carboxylate* (**4m**). White solid, m.p. 53 °C. Yield 72%. IR (KBr): *ν* (cm^−1^) 2930–3010 (C-H), 1373 (C=C), 1733, 1644 (C=O); ^1^H NMR (400 MHz, CDCl_3_): *δ* (ppm) 7.29–7.38 (m, 4H, Ar-H), 4.75 (br, 1H, N-CH), 3.70 (s, 3H, O-CH_3_), 3.20–3.30 (m, 2H, S-CH_2_), 2.02 (s, 3H, CH_3_), 1.97 (d, *J* = 18.5 Hz, 6H, C-(CH_3_)_2_); ^13^C NMR (100 MHz, CDCl_3_): *δ* (ppm) 170.63, 168.50, 136.79, 135.44, 128.85, 127.38, 73.31, 67.62, 52.90, 31.29, 29.55, 28.04; HRMS calcd. for [M + Na^+^] C_14_H_16_ClNO_3_S: 336.0432, found 336.0435.

*Methyl (R)-2,2-dimethyl-3-(p-nitrobenzoyl)thiazolidine-4-carboxylate* (**4n**). Yellow solid, m.p. 131 °C. Yield 87%. IR (KBr): *ν* (cm^−1^) 2906–3087 (C-H), 1728, 1630 (C=O), 1428–1589 (C=C); ^1^H NMR (400 MHz, CDCl_3_): *δ* (ppm) 8.25 (d, *J* = 8.7 Hz, 2H, Ar-H), 7.54 (d, *J* = 8.6 Hz, 2H, Ar-H), 4.62 (br, 1H, N-CH), 3.70 (s, 3H, O-CH_3_), 3.23–3.32 (m, 2H, S-CH_2_), 2.00 (d, *J* = 11.0 Hz, 6H, C-(CH_3_)_2_); ^13^C NMR (100 MHz, CDCl_3_): *δ* (ppm) 170.22, 167.26, 148.07, 144.08, 127.03, 123.99, 73.65, 67.37, 53.08, 31.38, 29.56, 27.77; HRMS calcd. for [M + Na^+^] C_14_H_16_N_2_O_5_S: 347.0672, found 347.0676.

*Methyl (R)-2,2-dimethyl-3-(m-methylbenzoyl)thiazolidine-4-carboxylate* (**4o**). White solid, m.p. 62–63 °C. Yield 65%. IR (KBr): *ν* (cm^−1^) 2914–3034. (C-H), 1713, 1628 (C=O), 1421.85 (C=C); ^1^H NMR (400 MHz, CDCl_3_): *δ* (ppm) 7.11–7.26 (m, 4H, Ar-H), 4.80 (br, 1H, N-CH), 3.70 (s, 3H, O-CH_3_), 3.17–3.30 (m, 2H, S-CH_2_), 1.99 (d, *J* = 18.5 Hz, 6H, C-(CH_3_)_2_); ^13^C NMR (100 MHz, CDCl_3_): *δ* (ppm) 170.93, 169.73, 138.43, 130.11, 128.46, 126.31, 122.69, 73.21, 67.74, 52.73, 31.32, 29.64, 28.02, 21.37; HRMS calcd. for [M+Na^+^] C_15_H_19_NO_3_S: 316.0978, found 316.0976.

*Methyl (R)-3-(o-methoxybenzoyl)-2,2-dimethylthiazolidine-4-carboxylate* (**4p**). White solid, m.p. 93 °C. Yield 69%. IR (KBr): *ν* (cm^−1^) 2843–2979 (C-H), 1740, 1644 (C=O), 1437–1599 (C=C); ^1^H NMR (400 MHz, CDCl_3_): *δ* (ppm) 7.25–7.34, 6.87–6.97 (m, 4H, Ar-H), 4.61 (d, *J* = 4.7 Hz, 1H, N-CH), 3.86 (s, 3H, O-CH_3_), 3.64 (s, 3H, COOCH_3_), 3.20–3.32 (m, 2H, S-CH_2_), 2.03 (d, *J* = 18.3 Hz, 6H, C-(CH_3_)_2_); ^13^C NMR (100 MHz, CDCl_3_): *δ* (ppm) 170.96, 166.97, 154.13, 130.48, 128.29, 127.97, 121.01, 110.95, 73.24, 67.11, 55.83, 52.56, 31.57, 29.23, 27.96; HRMS calcd. for [M + H^+^] C_15_H_19_NO_4_S: 310.1108, found 310.1110.

*Methyl (R)-3-(o-chlorobenzoyl)-2,2-dimethylthiazolidine-4-carboxylate* (**4q**). White solid, m.p. 135 °C. Yield 71%. IR (KBr): *ν* (cm^−1^) 2927–3004 (C-H), 1743, 1645 (C=O), 1393–1437 (C=C); ^1^H NMR (400 MHz, CDCl_3_): *δ* (ppm) 7.27–7.38 (m, 4H, Ar-H), 4.51 (d, *J* = 4.9 Hz, 1H, N-CH), 3.68 (s, 3H, O-CH_3_), 3.22–3.36 (m, 2H, S-CH_2_), 2.03 (d, *J* = 14.4 Hz, 6H, C-(CH_3_)_2_); ^13^C NMR (100 MHz, CDCl_3_): *δ* (ppm) 170.45, 165.74, 137.47, 130.31, 129.39, 129.07, 128.30, 127.21, 73.49, 67.24, 52.79, 31.69, 29.02, 27.82. HRMS calcd. for [M + H^+^] C_14_H_16_ClNO_3_S: 314.0612, found 314.0616.

*Methyl (R)-4-(2,2-dichloroacetyl)-1-thia-4-azaspiro[4.5]decane-3-carboxylate* (**4r**). White solid, m.p. 127 °C. Yield 48%. IR (KBr): *ν* (cm^−1^) 2937 (C-H), 1734, 1682 (C=O); ^1^H NMR (400 MHz, CDCl_3_): *δ* (ppm) 5.92 (s, 1H, CHCl_2_), 5.09 (d, *J* = 5.3 Hz, 1H, N-CH), 3.78 (s, 3H, O-CH_3_), 3.13–3.35 (m, 2H, S-CH_2_), 2.76–3.11, 1.16–1.84 (m, 10H, C_6_H_10_); ^13^C NMR (100 MHz, CDCl_3_): *δ* (ppm) 170.00, 161.25, 82.57, 67.33, 64.95, 53.44, 35.38, 33.54, 30.88, 25.53, 25.25, 24.44; HRMS calcd. for [M + H^+^] C_12_H_17_NO_3_SCl_2_: 326.0379, found 326.0376.

*Methyl (R)-3-(2,2-dichloroacetyl)-2,2-dimethylthiazolidine-4-carboxylate* (**4s**). White solid, m.p. 105 °C. Yield 41%. IR (KBr): *ν* (cm^−1^) 2927–3039 (C-H), 1736, 1685 (C=O); ^1^H NMR (400 MHz, CDCl_3_): *δ* (ppm) 5.99 (s, 1H, CHCl_2_), 5.10 (d, *J* = 5.1 Hz, 1H, N-CH), 3.85 (s, 3H, O-CH_3_), 3.30–3.42 (m, 2H, S-CH_2_), 1.89 (d, *J* = 17.7 Hz, 6H, C-(CH_3_)_2_); ^13^C NMR (100 MHz, CDCl_3_): *δ* (ppm) 169.84, 161.10, 74.95, 66.88, 65.07, 53.46, 31.40, 28.49, 27.25; HRMS calcd. for [M + H^+^] C_9_H_13_NO_3_SCl_2_: 286.0066, found 286.0061.

### 3.3. X-ray Diffraction

Single crystals were obtained by dissolving Compound **4q** in ethyl acetate and *n*-hexane and by allowing the solvent to slowly evaporate at 25 °C. X-ray data for **4q** were collected on a BRUKER D8 VENTURE X-diffractometer equipped with graphite monochromatic Mo K*α* radiation (*λ* = 0.71073 Å) at 273(2) K. The structure was solved by direct methods and refined by full-matrix least-squares on F^2^ using the SHELXL-2014/7 program (2014/7, BRUKER Inc.) [22]. Crystallographic data for the structural analysis have been deposited with the Cambridge Crystallographic Data Centre (CCDC No. 1551890). Copies of this information can be obtained free of charge from the Director, CCDC, 12 Union Road, Cambridge CB2 1EZ, UK (Fax: +44-1223-336033; E-mail: deposit@ccdc.cam.ac.uk or http://www.ccdc.cam.ac.uk). Selected bond lengths and angles for compound **4q** are presented in Supplementary Materials (Table S1).

Crystal data for Compound **4q**: C_14_H_16_ClNO_3_S (*M* = 313.79 g/mol): orthorhombic, space group *P*2_1_2_1_2_1_ (no. 19), *a* = 6.1350(2) Å, *b* = 13.1870(3) Å, *c* = 18.5868(5) Å, *V* = 1503.71(7) Å ^3^, *Z* = 4, *T* = 273(2) K, μ(Mo K*α*) = 0.399 mm^−1^, *D*_c_ = 1.386 g/cm^3^, 19190 reflections measured (6.18° ≤ 2*θ* ≤ 56.578°), 3722 unique (*R*_int_ = 0.0228, *R_σ_* = 0.0174), which were used in all calculations. The final *R*_1_ was 0.0299 (*I* > 2*σ* (*I*)) and *ωR*_2_ was 0.0822 (all data).

### 3.4. Biological Activity Texts

Maize seeds (Dongnong 253) were soaked in a solution of methyl (*R*)-*N*-benzoyl/dichloroacetyl-thiazolidine-4-carboxylate, **4** (25 mg/kg) overnight, and the untreated seeds were soaked in water. Afterwards, the seeds were germinated in a growing chamber (26.5 °C, 12 h of light, relative humidity 75%) overnight. Then, the seeds were sown in paper cups in which soil was mixed with a chlorimuron-ethyl solution (24 μg/kg) or water. Finally, the paper cup was incubated in a growing chamber (26.5 °C, 12 h of light, relative humidity 75%) for 7 days. The growth level of maize was determined to investigate the biological activity of methyl (*R*)-*N*-benzoyl/dichloroacetyl-thiazolidine-4-carboxylates. Each treatment was replicated three times in a completely randomized design. The physical data of compound **4** are presented in Supplementary Materials (Figure S78 and Tables S79–S100).

### 3.5. Determination of ALS Activity

The treatment of maize seeds and soil was the same as that in the biological activity tests. At 6 days after treatment, leaves of maize were collected to determine the ALS activity. ALS activity determination was carried out following the procedure of Kobayashi and Sugiyama (1991) with a few modifications [23]. One gram of maize leaves was grated in liquid nitrogen and extraction medium (3.0 mL), and the mixture was centrifuged at 25,000× *g* for 20 min. The supernatant (2.0 mL) was added to the extraction medium (3.0 mL) and (NH_4_)_2_SO_4_ (1.5 g). The mixture was centrifuged at 25,000× *g* for 20 min after staying at 4 °C for 2 h. The enzyme solution was obtained by dissolving the precipitation in dissolution medium (3.0 mL). The ALS activity was assayed in mixed solution, which was composed of 0.1 mL of 0.05 mol/L phosphate buffer (pH 7.0), 0.5 mL of the reaction medium and 0.4 mL of the enzyme solution. After incubation at 35 °C for 1 h, the reaction was terminated by the addition of 0.1 mL of 3 mol/L H_2_SO_4_. Afterwards, the decarboxylation was carried out at 60 °C for 15 min. ALS activity was expressed as the amount of acetylmethylcarbinol per hour per milligram protein and the content of acetylmethylcarbinol was measured at 530 nm. Protein content was measured by the methods described by Bradford (1976) [24].

### 3.6. Molecular Docking Studies

The 3D molecular structures of chlorimuron-ethyl and Compound **4e** were built by the sketch module of the SYBYL-X 2.0 program package (Tripos, St. Louis, MO, USA). In addition, Gasteiger–Huckel charges were calculated after optimizing the molecules. The crystal structure of ALS was provided by the Protein Data Bank (PDB ID 1N0H). Accelrys Discovery Studio 2.5 provided the CDOCKER method for the docking modeling. Some co-crystallized small molecules and water were removed from the protein structure and the CHARMM force field was used to minimize the protein before docking. After the protein preparation, the active site for the docking studies was defined with a range of 13.0 Å from the center of the known ligand. The obtained receptor was used as the “Input Receptor.” Chlorimuron-ethyl and Compound **4e** were chosen as the “Input Ligand.” During the docking process, the top 10 conformations were saved for each ligand based on negative CDOCKER_ENERGT value after energy minimization. Finally, the default values were used in DS 2.5 if not mentioned otherwise.

## 4. Conclusions

In conclusion, complete details of the synthesis of methyl (*R*)-*N*-benzoyl/dichloroacetyl-thiazolidine-4-carboxylates from simple materials are presented in this paper. The bioactivities of these compounds as herbicide safeners were examined in maize. The ALS activity assay confirmed the positive effect of the title compounds on ALS activity. The bioactivity results showed that **4e** exhibited a significant protective effect to maize from injury of chlorimuron-ethyl, which was also confirmed by molecular docking modeling.

## Figures and Tables

**Table 1:** Yields of title compounds.

Compound	R^1^	R^2^	R^3^	Yield/%	Compound	R^1^	R^2^	R^3^	Yield/%
**4a**	(CH_2_)_5_		*p*-NO_2_C_6_H_4_	91	**4k**	(CH_2_)_4_		*o*-ClC_6_H_4_	70
**4b**	(CH_2_)_5_		2,4-Cl_2_C_6_H_3_	81	**4l**	CH_3_	CH_3_	2,4-Cl_2_C_6_H_3_	78
**4c**	(CH_2_)_5_		*p*-ClC_6_H_4_	77	**4m**	CH_3_	CH_3_	*p*-ClC_6_H_4_	72
**4d**	(CH_2_)_5_		*m*-CH_3_C_6_H_4_	69	**4n**	CH_3_	CH_3_	*p*-NO_2_C_6_H_4_	87
**4e**	(CH_2_)_5_		*o*-OCH_3_C_6_H_4_	71	**4o**	CH_3_	CH_3_	*m*-CH_3_C_6_H_4_	65
**4f**	(CH_2_)_5_		C_6_H_5_	75	**4p**	CH_3_	CH_3_	*o*-OCH_3_C_6_H_4_	69
**4g**	(CH_2_)_4_		*p*-ClC_6_H_4_	67	**4q**	CH_3_	CH_3_	*o*-ClC_6_H_4_	71
**4h**	(CH_2_)_4_		*p*-NO_2_C_6_H_4_	83	**4r**	(CH_2_)_5_		CHCl_2_	48
**4i**	(CH_2_)_4_		2,4-Cl_2_C_6_H_3_	72	**4s**	CH_3_	CH_3_	CHCl_2_	41
**4j**	(CH_2_)_4_		*m*-CH_3_C_6_H_4_	65					

**Table 2:** Protective effects of Compound **4** to maize ^a,b,c^.

Compound	Recovery of Plant Height (%)	Recovery of Root Length (%)	Recovery of Plant Weight (%)	Recovery of Root Weight (%)
**R-28725**	86.22 ± 1.37	84.73 ± 0.98	87.47 ± 1.12	83.61 ± 1.98
**4a**	42.90 ± 1.22	60.52 ± 1.37	45.96 ± 1.11	68.11 ± 1.82
**4b**	59.96 ± 1.09	45.53 ± 1.27	47.16 ± 1.24	68.48 ± 0.63
**4c**	80.33 ± 0.82	86.48 ± 0.87	94.61 ± 1.43	85.79 ± 1.62
**4d**	67.97 ± 1.02	41.97 ± 1.31	78.25 ± 0.98	41.98 ± 1.05
**4e**	89.08 ± 0.65	91.20 ± 0.89	98.92 ± 1.09	93.70 ± 1.22
**4f**	62.05 ± 0.21	64.07 ± 0.33	73.75 ± 1.39	51.56 ± 0.52
**4g**	85.86 ± 0.97	87.53 ± 1.43	85.62 ± 1.26	83.39 ± 0.92
**4h**	57.08 ± 0.69	46.83 ± 1.07	41.26 ± 0.73	59.74 ± 0.62
**4i**	45.64 ± 1.62	55.60 ± 0.55	53.93 ± 1.29	57.56 ± 0.54
**4j**	57.41 ± 1.26	66.61 ± 1.92	71.15 ± 1.42	67.59 ± 1.65
**4k**	41.82 ± 0.68	46.24 ± 0.23	55.83 ± 1.05	55.13 ± 0.13
**4l**	20.60 ± 0.69	15.09 ± 0.32	15.04 ± 1.22	25.27 ± 1.24
**4m**	48.99 ± 0.57	66.04 ± 1.13	48.27 ± 1.06	47.83 ± 0.36
**4n**	25.02 ± 0.92	29.16 ± 0.91	17.01 ± 0.61	24.94 ± 0.85
**4o**	34.12 ± 1.32	38.30 ± 0.45	39.58 ± 1.02	56.35 ± 1.13
**4p**	76.00 ± 1.16	61.09 ± 1.75	68.93 ± 1.26	55.57 ± 1.44
**4q**	23.20 ± 0.53	14.56 ± 0.32	19.04 ± 1.42	25.25 ± 0.54
**4r**	57.74 ± 1.31	39.46 ± 1.44	31.46 ± 0.88	46.34 ± 1.32
**4s**	33.22 ± 1.65	36.30 ± 1.44	45.75 ± 1.54	37.89 ± 0.74

^a^ Data are means of three replicates, mean ± standard deviation; ^b^ $\mathrm{Recovery}\;\mathrm{Rate}\;(\%)\;=\frac{\mathrm{Treatment}\;\mathrm{with}\;\mathrm{compounds}\;—\;\mathrm{Treatment}\;\mathrm{with}\;\mathrm{Chlorimur}\;\mathrm{on}-\mathrm{ethyl}}{\mathrm{Contrast}\;—\;\mathrm{Treatment}\;\mathrm{with}\;\mathrm{Chlorimuron}-\mathrm{ethyl}}$; ^c^ Contrast was treated by water.

**Table 3:** Effect of Compounds **4a**–**s** and chlorimuron-ethyl on the acetolactate synthase (ALS) activity of maize.

Treatment	ALS Activity (nmol h^−1^ mg^−1^ Protein) ^a^	Treatment	ALS Activity (nmol h^−1^ mg^−1^ Protein) ^a^	Treatment	ALS Activity (nmol h^−1^ mg^−1^ Protein) ^a^
**Control ^b^**	0.091 ± 0.002	chlorimuron-ethyl + 4f	0.075 ± 0.003	**chlorimuron-ethyl + 4n**	0.059 ± 0.003
**chlorimuron-ethyl**	0.046 ± 0.003	**chlorimuron-ethyl + 4g**	0.079 ± 0.003	**chlorimuron-ethyl + 4o**	0.064 ± 0.001
**chlorimuron-ethyl + R-28725**	0.084 ± 0.002	**chlorimuron-ethyl + 4h**	0.071 ± 0.002	**chlorimuron-ethyl + 4p**	0.071 ± 0.001
**chlorimuron-ethyl + 4a**	0.064 ± 0.001	**chlorimuron-ethyl + 4i**	0.068 ± 0.002	**chlorimuron-ethyl + 4q**	0.055 ± 0.002
**chlorimuron-ethyl + 4b**	0.062 ± 0.004	**chlorimuron-ethyl + 4j**	0.077 ± 0.002	**chlorimuron-ethyl + 4r**	0.064 ± 0.002
**chlorimuron-ethyl + 4c**	0.081 ± 0.002	**chlorimuron-ethyl + 4k**	0.068 ± 0.001	**chlorimuron-ethyl + 4s**	0.062 ± 0.001
**chlorimuron-ethyl + 4d**	0.065 ± 0.002	**chlorimuron-ethyl + 4l**	0.058 ± 0.001		
**chlorimuron-ethyl + 4e**	0.085 ± 0.002	**chlorimuron-ethyl + 4m**	0.068 ± 0.003		

^a^ Data are means of three replicates, mean ± standard deviation; ^b^ Control was treated by water.

## Supplementary Materials

Supplementary Materials are available online. Table S1; Figures S2–S78 and Tables S79–S100.
