# Supplementary material for: Design, Synthesis, and Safener Activity of Novel Methyl (R)-N-Benzoyl/Dichloroacetyl-Thiazolidine-4-Carboxylates
Source: Molecules. 2018 Jan 12;23(1):155. doi: 10.3390/molecules23010155 (PMC6017158; doi:10.3390/molecules23010155)
Supplement: Supplementary file 1 [file molecules-23-00155-s001.pdf]

Design, Synthesis and Safener Activity of Novel Methyl (R)-*N*-benzoyl/dichloroacetyl-thiazolidine-4-carboxylates

Li-Xia Zhao, Hao Wu, Yue-Li Zou, Qing-Rui Wang, Ying Fu, Chun-Yan Li, and Fei Ye *

Selected bond lengths and angles for compound **4q** were listed below:

Table S1. Bond lengths (Å) and bond angles (°) in compound **4q**.

C(1)-C(2)	1.390(4)	C(2)-C(1)-C(6)	121.4(3)	H(10A)-C(10)-H(10C)	109.5
C(1)-C(6)	1.393(3)	C(2)-C(1)-Cl(1)	119.6(2)	H(10B)-C(10)-H(10C)	109.5
C(1)-Cl(1)	1.743(3)	C(6)-C(1)-Cl(1)	119.00(18)	C(12)-C(11)-S(1)	102.82(13)
C(2)-C(3)	1.371(5)	C(3)-C(2)-C(1)	118.9(3)	C(12)-C(11)-H(11A)	111.2
C(2)-H(2)	0.9300	C(3)-C(2)-H(2)	120.6	S(1)-C(11)-H(11A)	111.2
C(3)-C(4)	1.376(5)	C(1)-C(2)-H(2)	120.6	C(12)-C(11)-H(11B)	111.2
C(3)-H(3)	0.9300	C(2)-C(3)-C(4)	120.9(2)	S(1)-C(11)-H(11B)	111.2
C(4)-C(5)	1.388(3)	C(2)-C(3)-H(3)	119.5	H(11A)-C(11)-H(11B)	109.1
C(4)-H(4)	0.9300	C(4)-C(3)-H(3)	119.5	N(1)-C(12)-C(11)	105.67(15)
C(5)-C(6)	1.384(3)	C(3)-C(4)-C(5)	120.0(3)	N(1)-C(12)-C(13)	110.50(14)
C(5)-H(5)	0.9300	C(3)-C(4)-H(4)	120.0	C(11)-C(12)-C(13)	113.13(16)
C(6)-C(7)	1.503(3)	C(5)-C(4)-H(4)	120.0	N(1)-C(12)-H(12)	109.1
C(7)-O(1)	1.220(2)	C(6)-C(5)-C(4)	120.4(3)	C(11)-C(12)-H(12)	109.1
C(7)-N(1)	1.355(2)	C(6)-C(5)-H(5)	119.8	C(13)-C(12)-H(12)	109.1
C(8)-N(1)	1.500(2)	C(4)-C(5)-H(5)	119.8	O(2)-C(13)-O(3)	124.53(19)
C(8)-C(10)	1.520(3)	C(5)-C(6)-C(1)	118.4(2)	O(2)-C(13)-C(12)	124.53(18)
C(8)-C(9)	1.530(3)	C(5)-C(6)-C(7)	120.3(2)	O(3)-C(13)-C(12)	110.91(16)
C(8)-S(1)	1.833(2)	C(1)-C(6)-C(7)	120.98(19)	O(3)-C(14)-H(14A)	109.5
C(9)-H(9A)	0.9600	O(1)-C(7)-N(1)	123.45(18)	O(3)-C(14)-H(14B)	109.5
C(9)-H(9B)	0.9600	O(1)-C(7)-C(6)	118.79(17)	H(14A)-C(14)-H(14B)	109.5
C(9)-H(9C)	0.9600	N(1)-C(7)-C(6)	117.74(16)	O(3)-C(14)-H(14C)	109.5
C(10)-H(10A)	0.9600	N(1)-C(8)-C(10)	113.21(17)	H(14A)-C(14)-H(14C)	109.5
C(10)-H(10B)	0.9600	N(1)-C(8)-C(9)	108.31(17)	H(14B)-C(14)-H(14C)	109.5
C(10)-H(10C)	0.9600	C(10)-C(8)-C(9)	112.5(2)	C(7)-N(1)-C(12)	122.97(15)
C(11)-C(12)	1.525(3)	N(1)-C(8)-S(1)	103.36(12)	C(7)-N(1)-C(8)	121.13(15)
C(11)-S(1)	1.801(2)	C(10)-C(8)-S(1)	107.98(16)	C(12)-N(1)-C(8)	115.72(15)

C(11)-H(11A)	0.9700	C(9)-C(8)-S(1)	111.15(16)	C(13)-O(3)-C(14)	115.13(19)
C(11)-H(11B)	0.9700	C(8)-C(9)-H(9A)	109.5	C(11)-S(1)-C(8)	91.09(9)
C(12)-N(1)	1.460(2)	C(8)-C(9)-H(9B)	109.5		
C(12)-C(13)	1.527(2)	H(9A)-C(9)-H(9))	109.5		
C(12)-H(12)	0.9800	C(8)-C(9)-H(9C)	109.5		
C(13)-O(2)	1.197(3)	H(9A)-C(9)-H(9C)	109.5		
C(13)-O(3)	1.326(2)	H(9B)-C(9)-H(9C)	109.5		
C(14)-O(3)	1.452(3)	C(8)-C(10)-H(10A)	109.5		
C(14)-H(14A)	0.9600	C(8)-C(10)-H(10B)	109.5		
C(14)-H(14B)	0.9600	H(10A)-C(10)-H(10	109.5		
C(14)-H(14C)	0.9600	C(8)-C(10)-H(10C)	109.5		
Symmetry transformations used to generate equivalent atoms.					

The IR, ^{1}H NMR, ^{13}C-NMR and HMRS spectra of compounds **4a-s** were listed below:

Methyl (R)-4-(*p*-nitrobenzoyl)-1-thia-4-azaspiro[4.5]decane-3-carboxylate (4a).

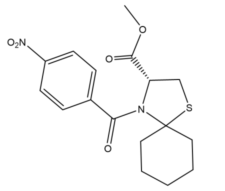

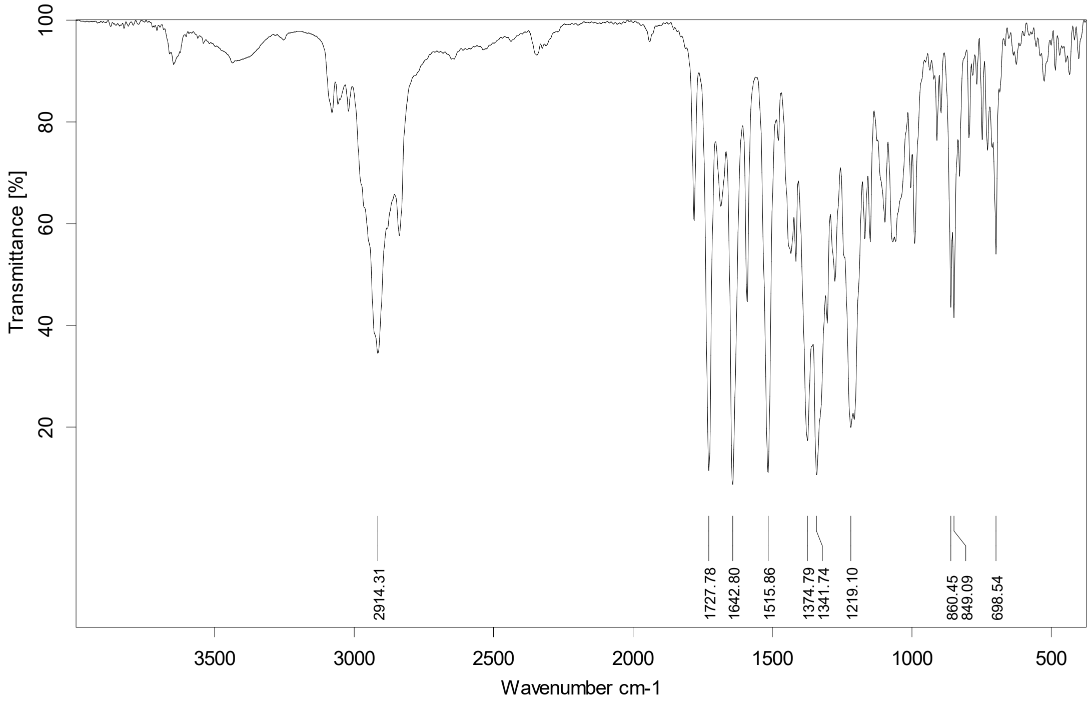

Figure S2. IR spectrum of compound **4a**.

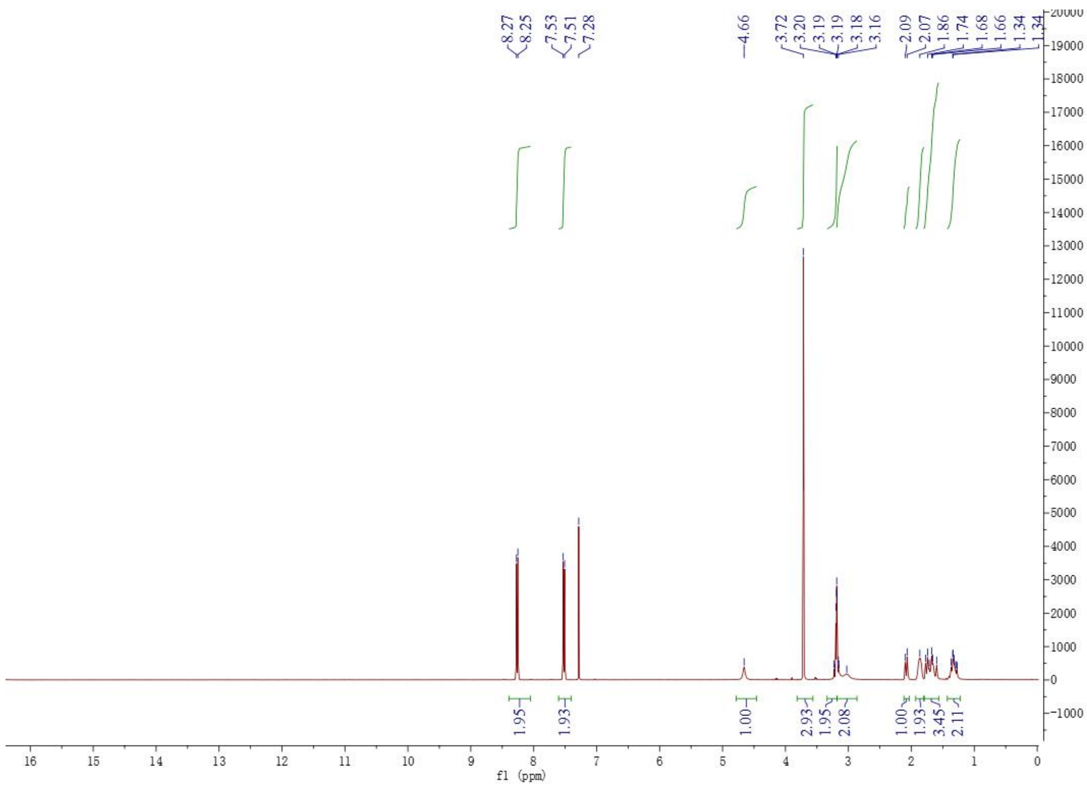

Figure S3. ^{1}H NMR spectrum of compound **4a**.

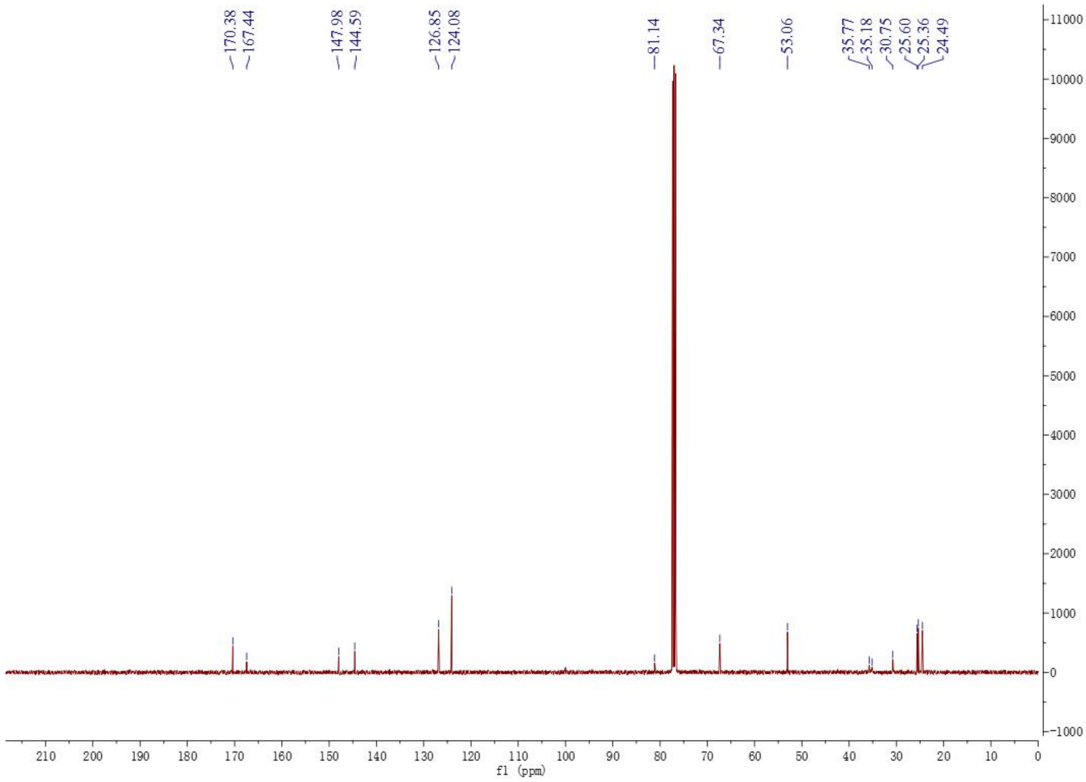

Figure S4. ^{13}C NMR spectrum of compound **4a** in $CDCl_3$.

Error=1.3 ppm

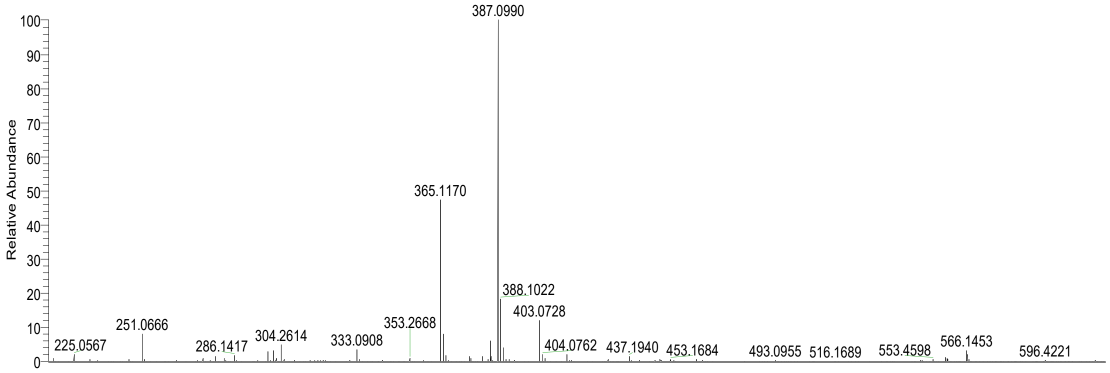

Figure S5. HMRS spectrum of compound **4a**.

Methyl (R)-4-(2,4-dichlorobenzoyl)-1-thia-4-azaspiro[4.5]decane-3-carboxylate (4b).

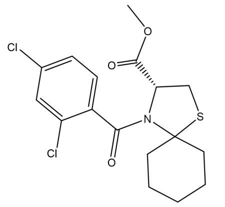

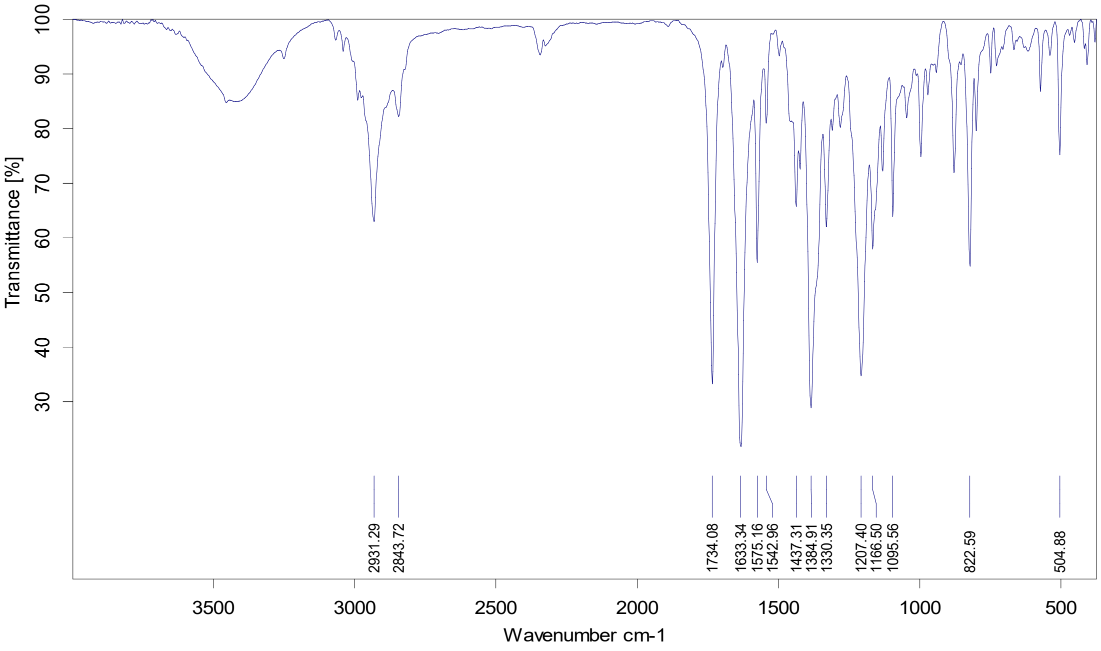

Figure S6. IR spectrum of compound **4b**.

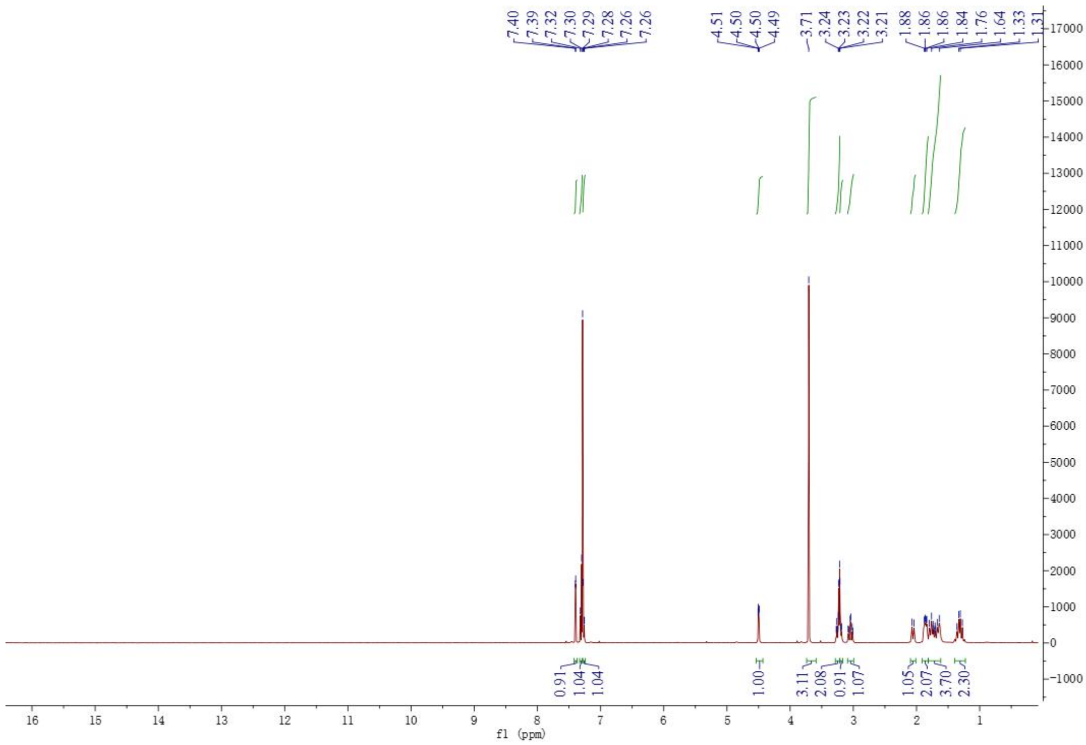

Figure S7. ^{1}H NMR spectrum of compound **4b**.

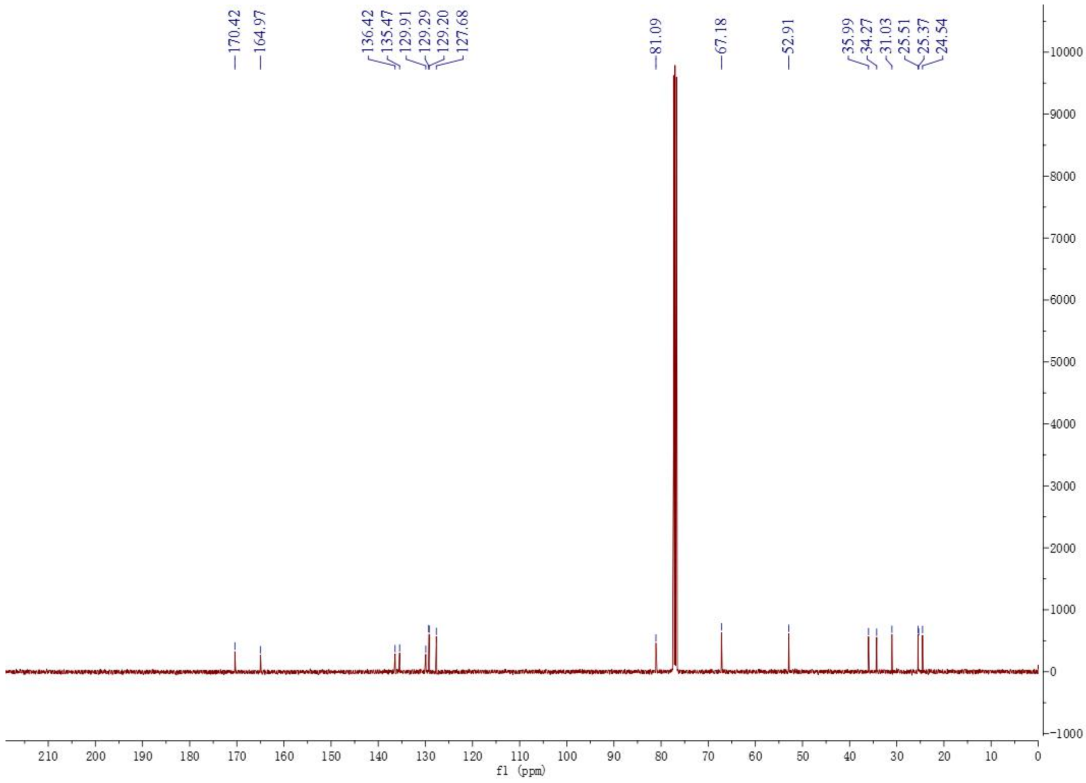

Figure S8. ^{13}C NMR spectrum of compound **4b** in $CDCl_3$.

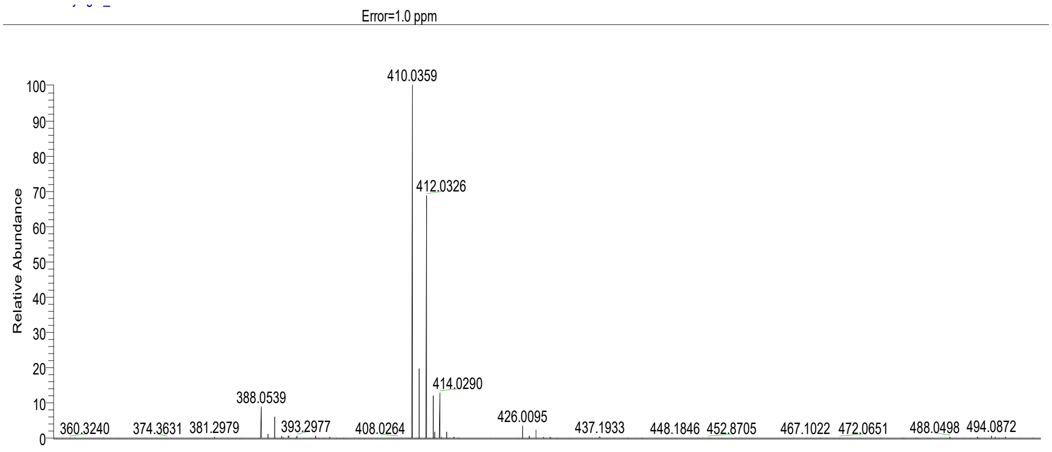

Figure S9. HMRS spectrum of compound **4b**.

Methyl (R)-4-(*p*-chlorobenzoyl)-1-thia-4-azaspiro[4.5]decane-3-carboxylate (4c).

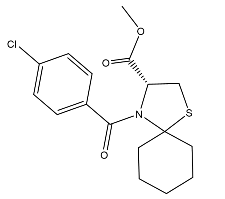

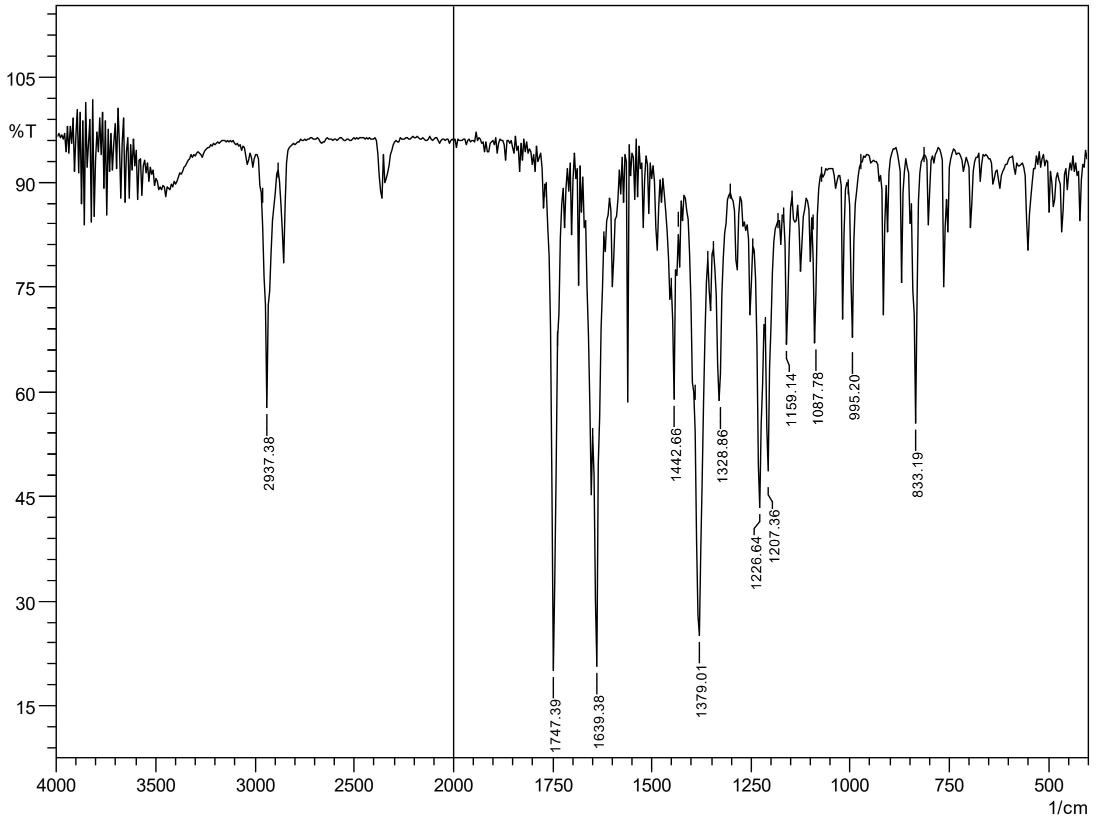

Figure S10. IR spectrum of compound **4c**.

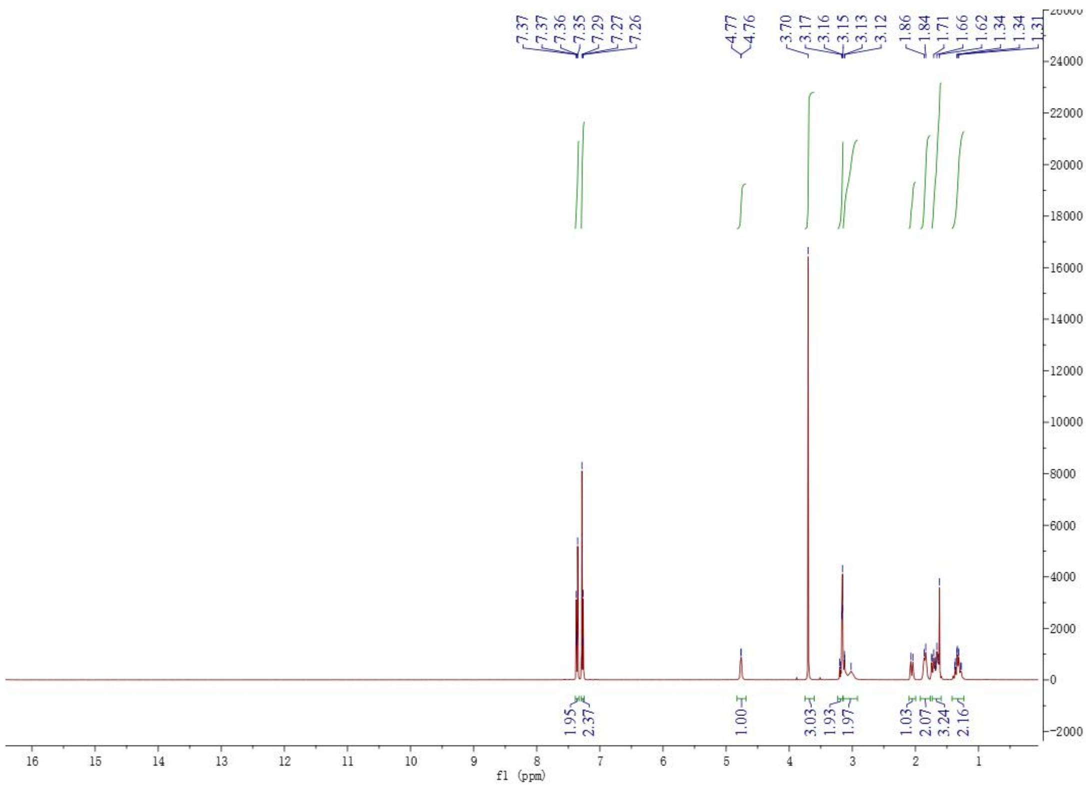

Figure S11. ^{1}H NMR spectrum of compound **4c**.

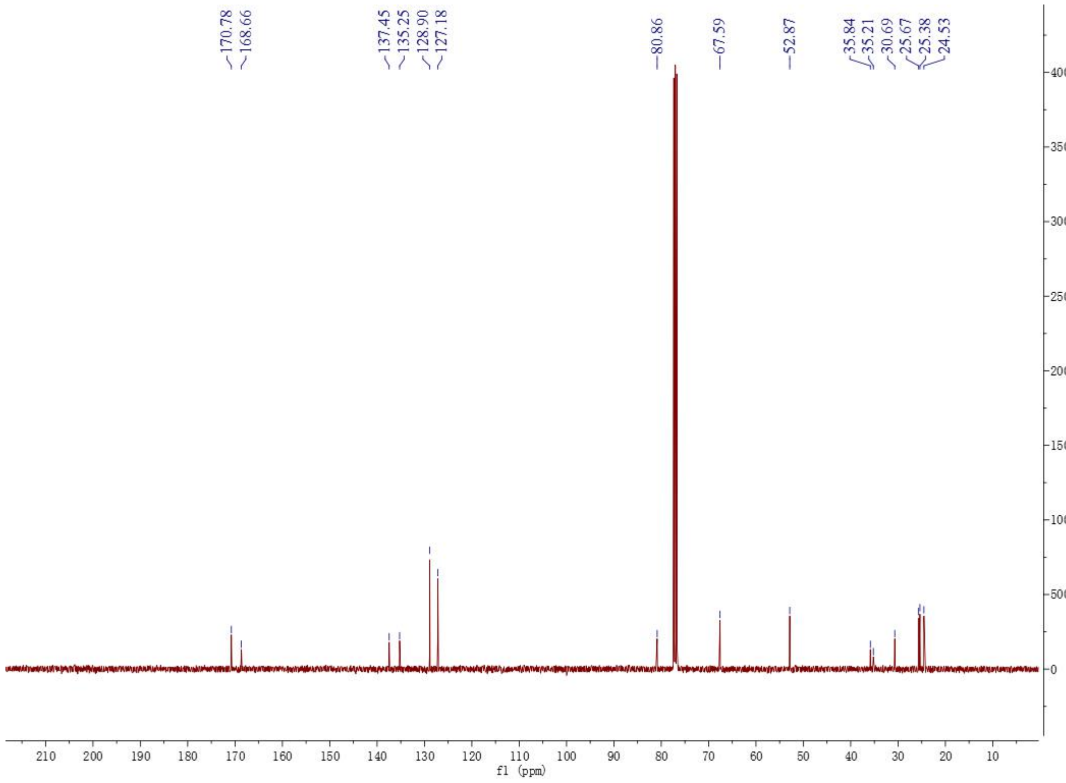

Figure S12. ^{13}C NMR spectrum of compound **4c** in $CDCl_3$.

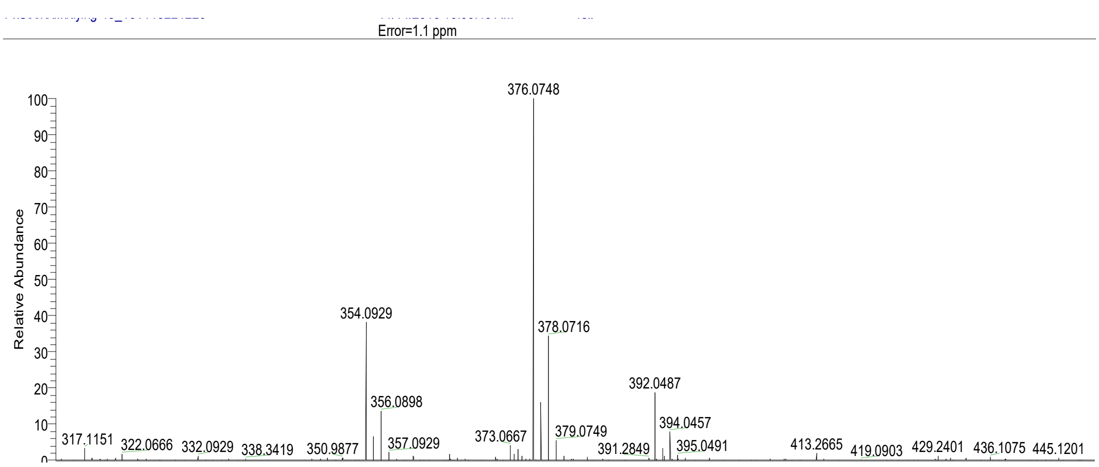

Figure S13. HMRS spectrum of compound **4c**.

Methyl (R)-4-(*m*-methylbenzoyl)-1-thia-4-azaspiro[4.5]decane-3-carboxylate (4d).

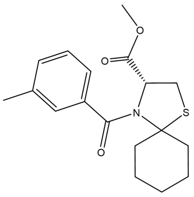

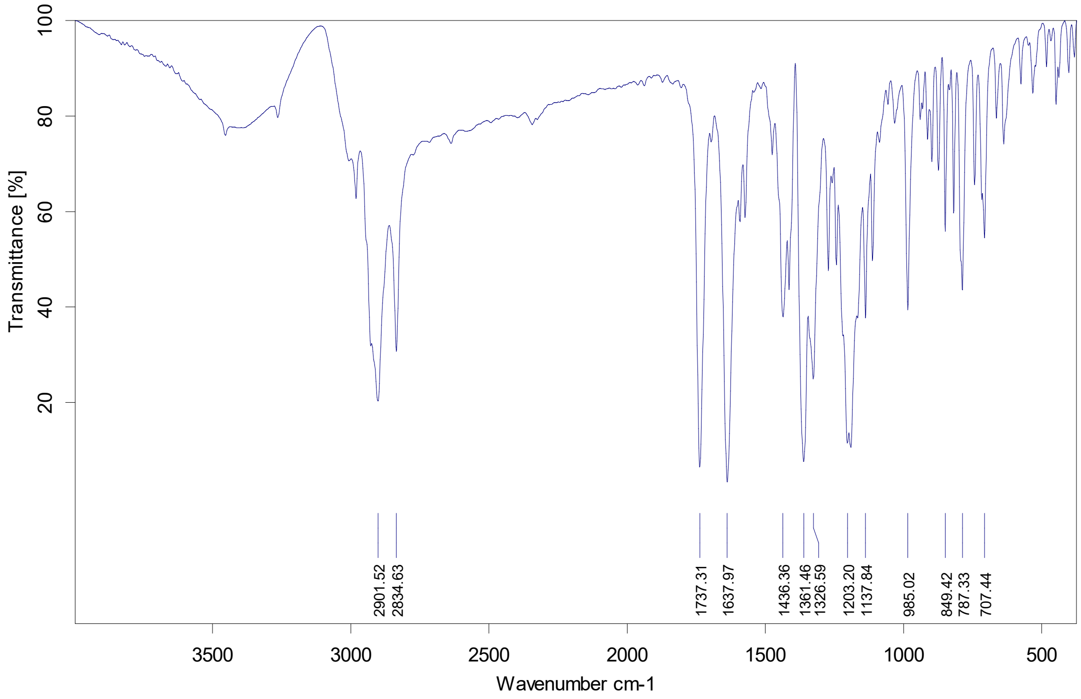

Figure S14. IR spectrum of compound **4d**.

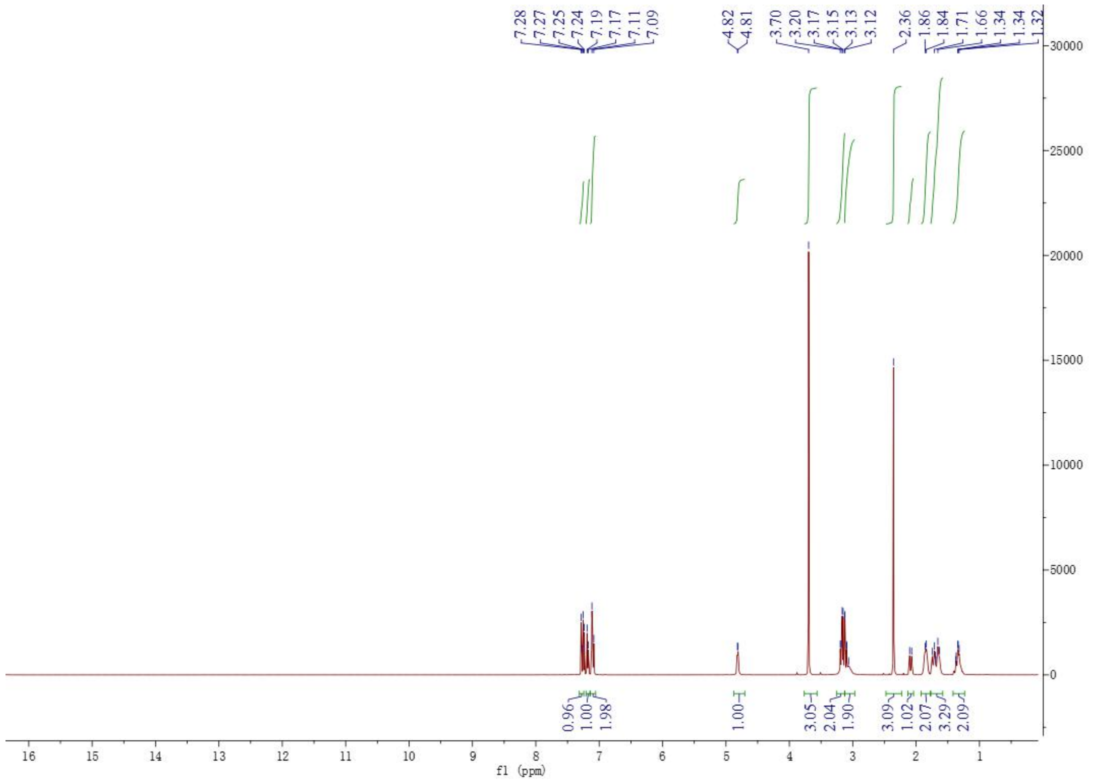

Figure S15. ^{1}H NMR spectrum of compound **4d**.

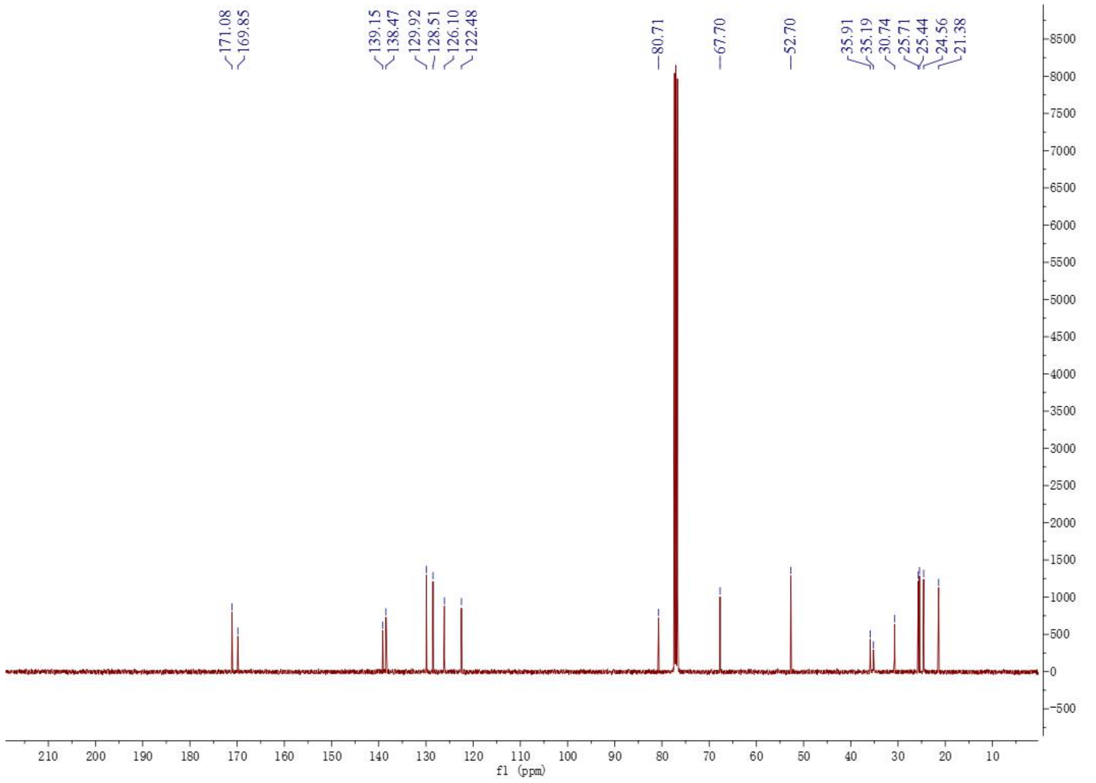

Figure S16. ^{13}C NMR spectrum of compound **4d** in $CDCl_3$.

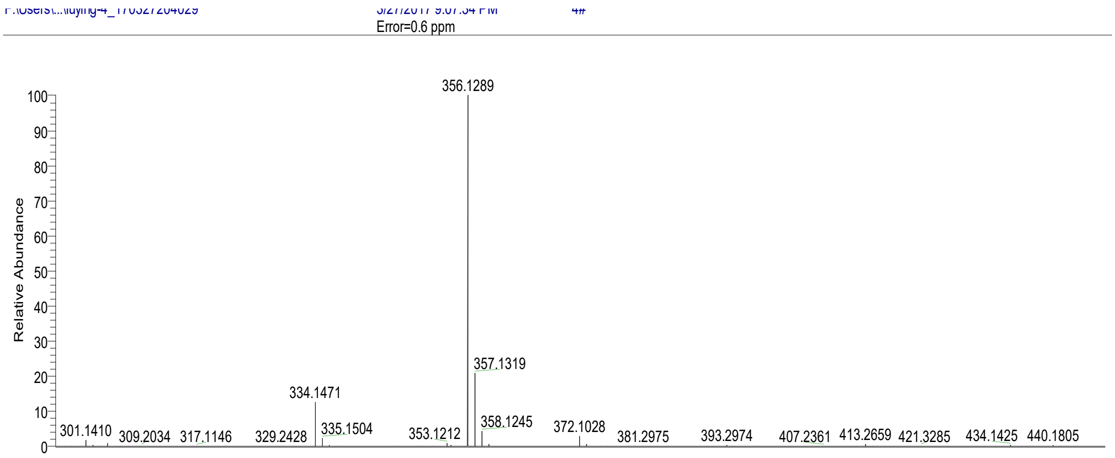

Figure S17. HMRS spectrum of compound **4d**.

Methyl (R)-4-(*o*-methoxybenzoyl)-1-thia-4-azaspiro[4.5]decane-3-carboxylate (4e).

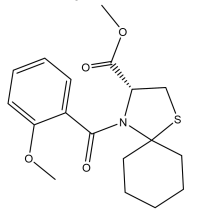

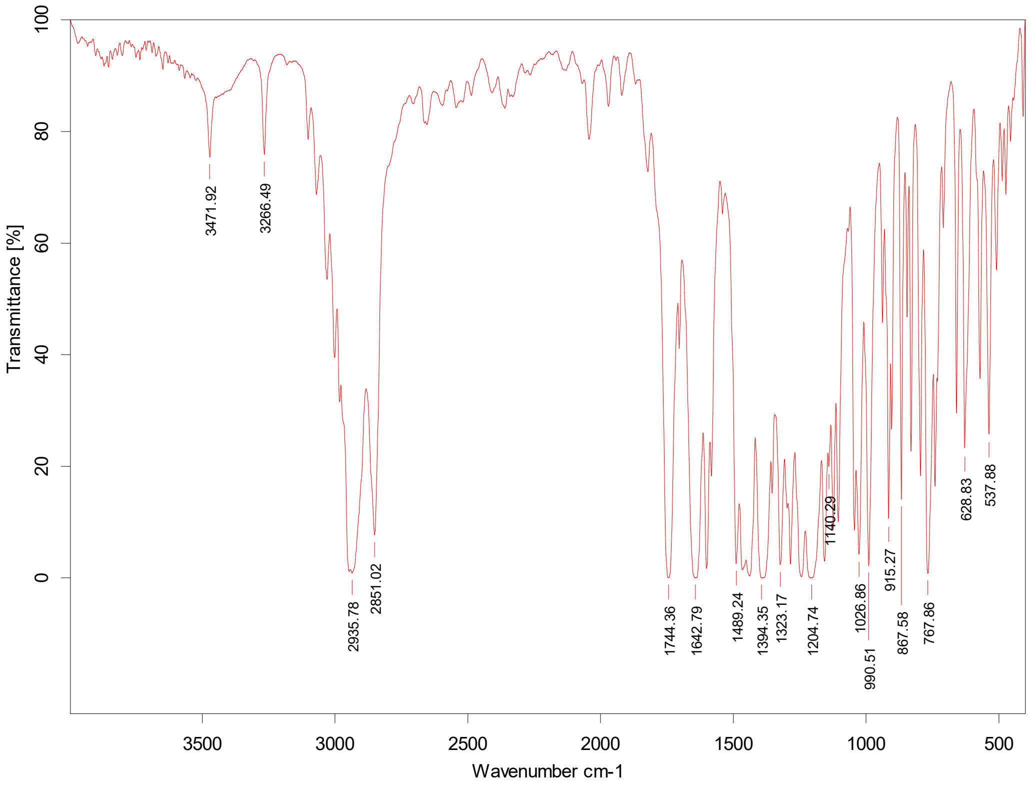

Figure S18. IR spectrum of compound **4e**.

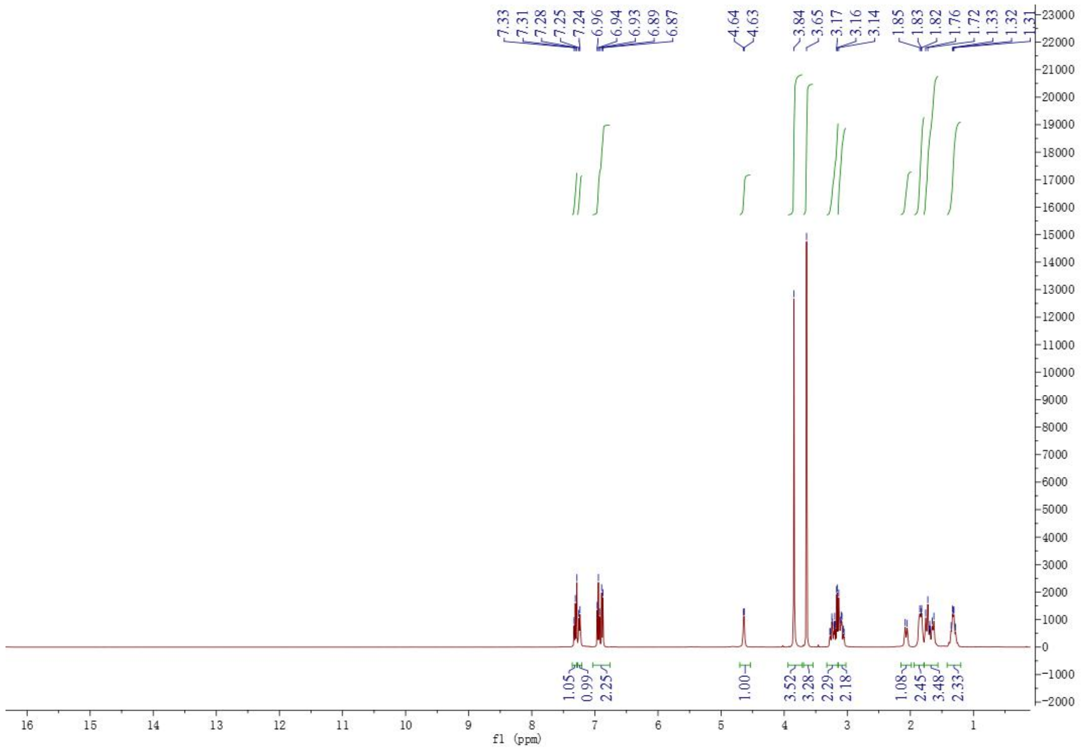

Figure S19. ^{1}H NMR spectrum of compound **4e**.

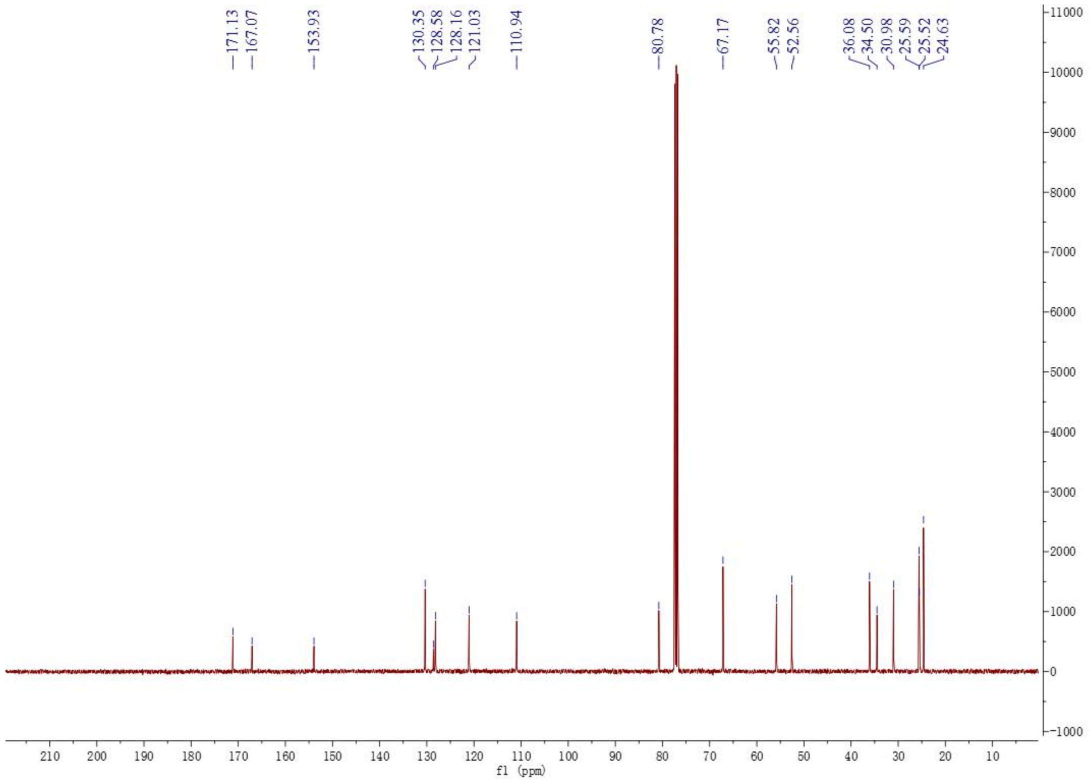

Figure S20. ^{13}C NMR spectrum of compound **4e** in $CDCl_3$.

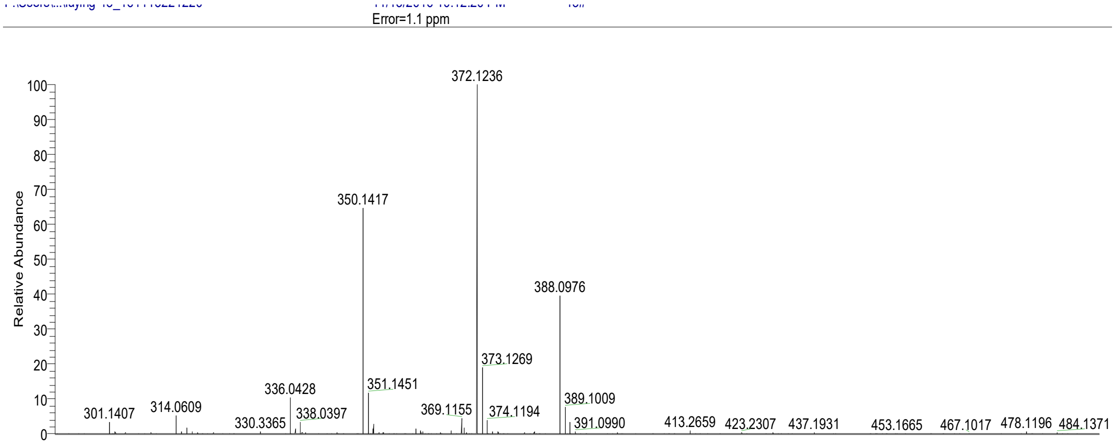

Figure S21. HMRS spectrum of compound **4e**.

Methyl (R)-4-benzoyl-1-thia-4-azaspiro[4.5]decane-3-carboxylate (4f).

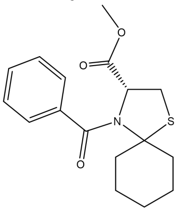

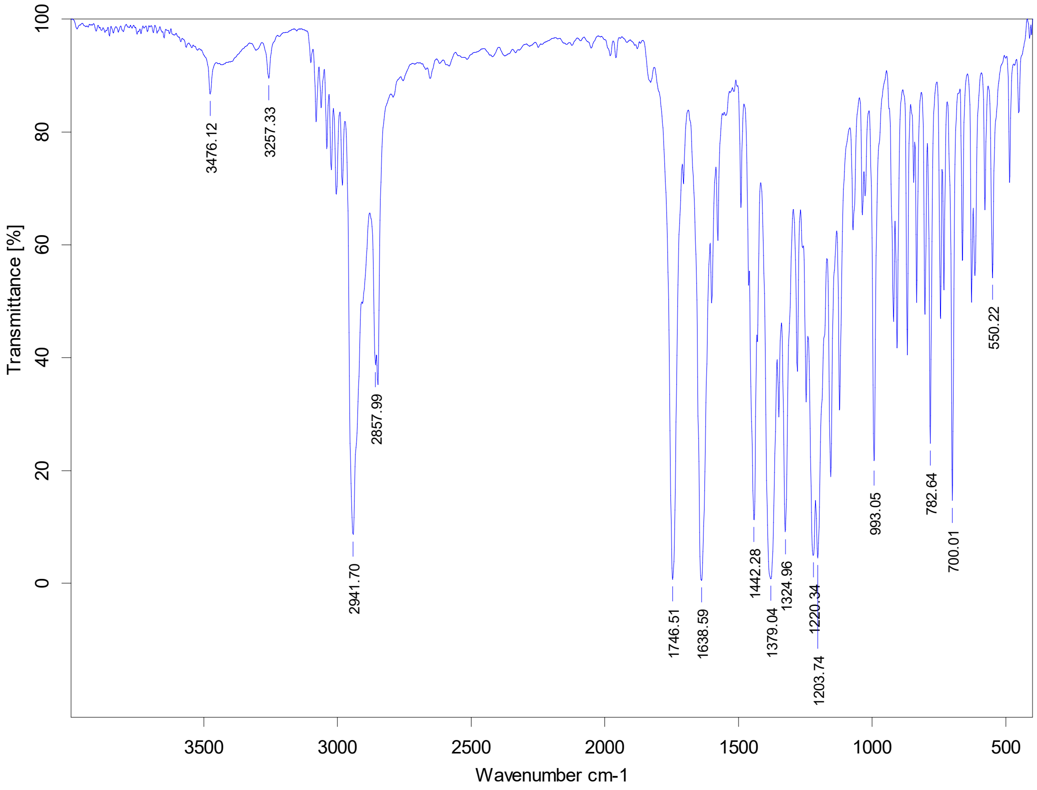

Figure S22. IR spectrum of compound **4f**.

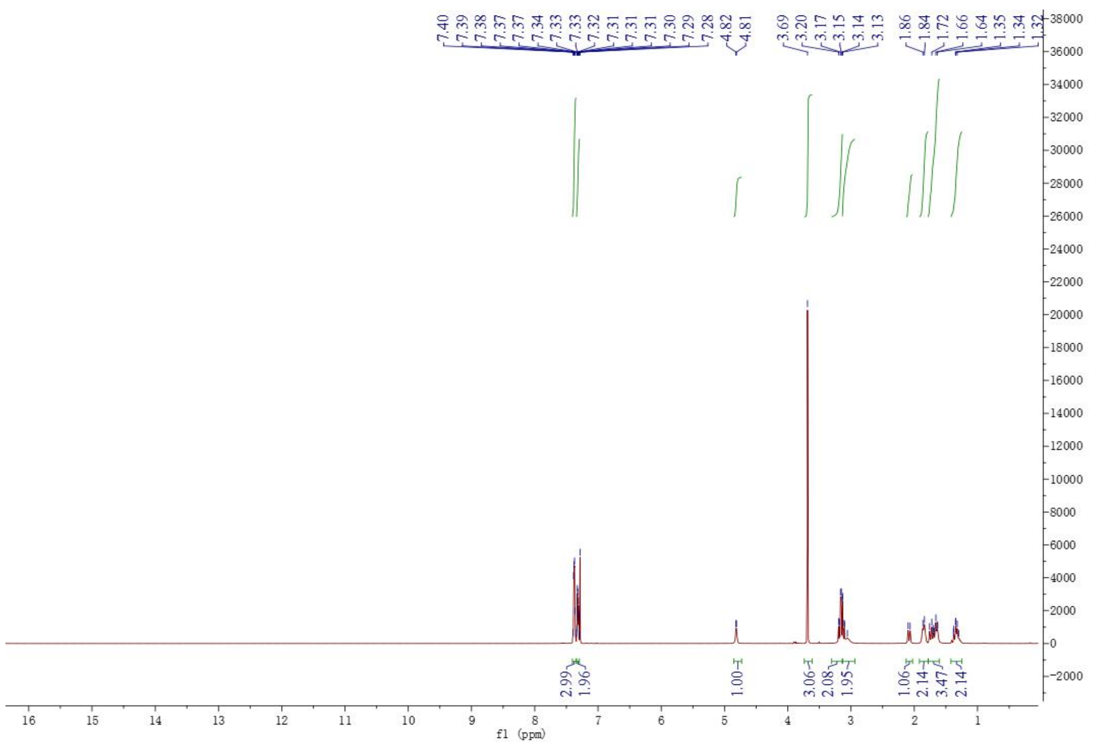

Figure S23. ^{1}H NMR spectrum of compound **4f**.

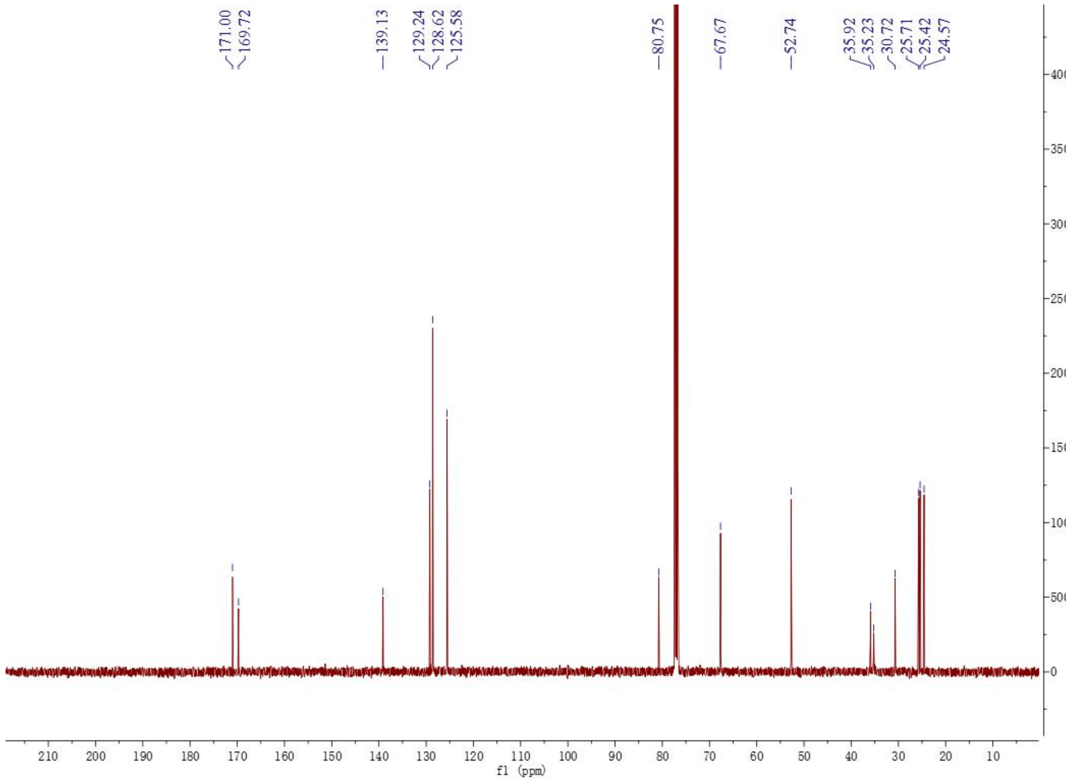

Figure S24. ^{13}C NMR spectrum of compound **4f** in $CDCl_3$.

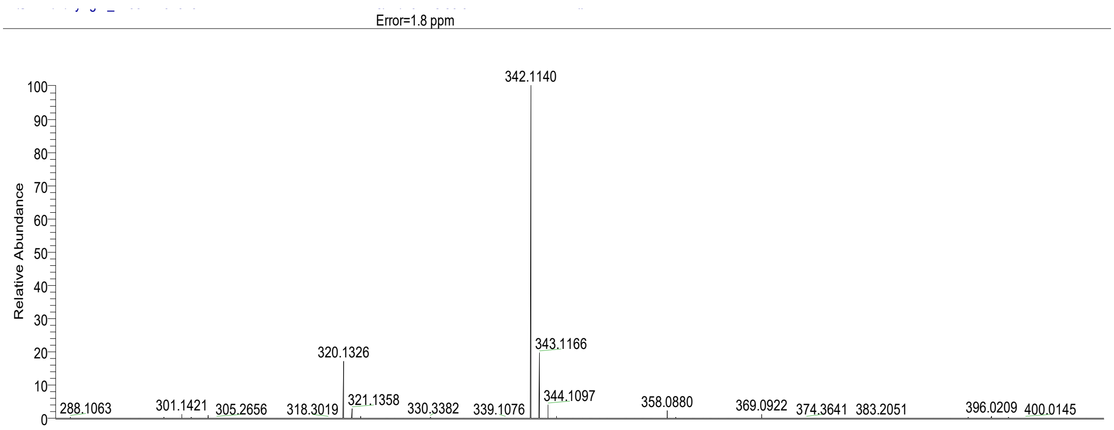

Figure S25. HMRS spectrum of compound **4f**.

Methyl (R)-4-(*p*-chlorobenzoyl)-1-thia-4-azaspiro[4.4]nonane-3-carboxylate (4g).

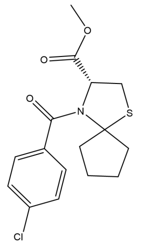

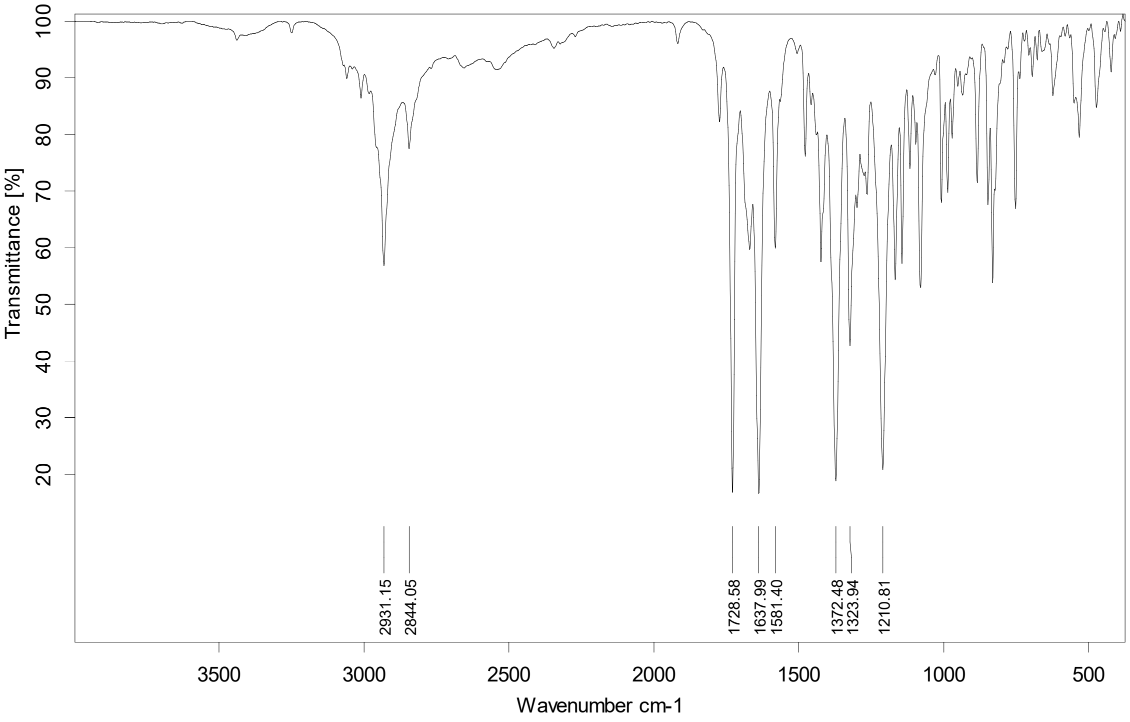

Figure S26. IR spectrum of compound **4g**.

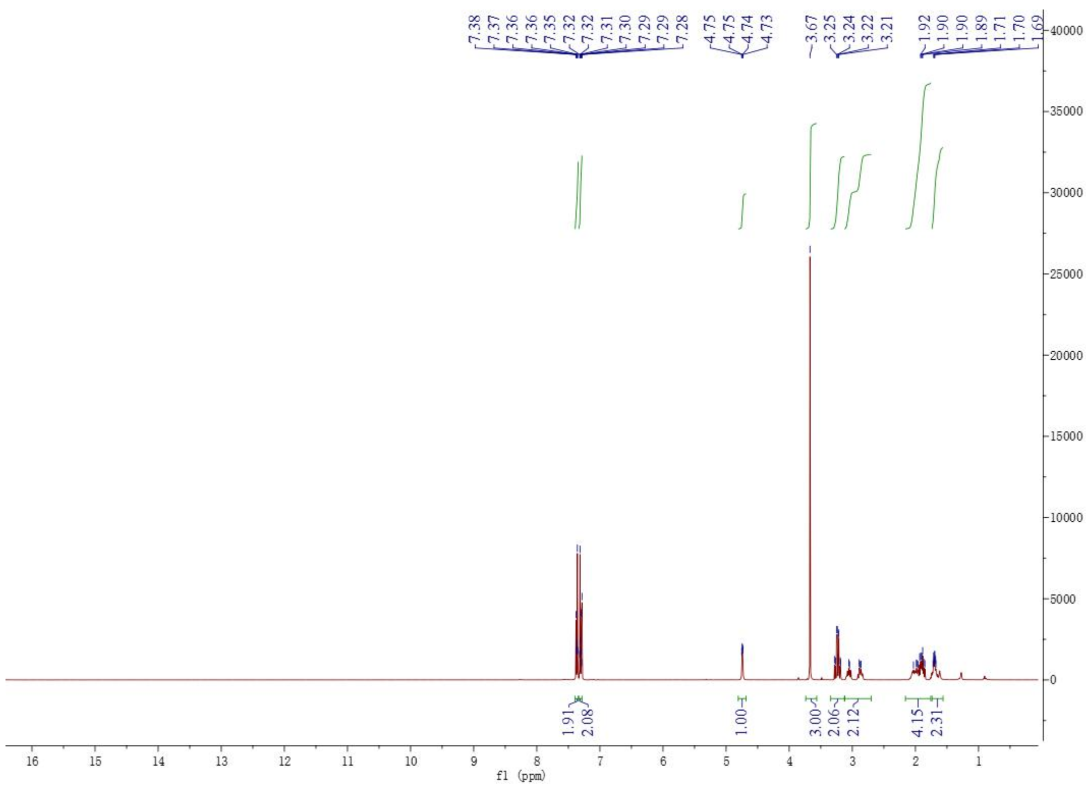

Figure S27. ^{1}H NMR spectrum of compound **4g**.

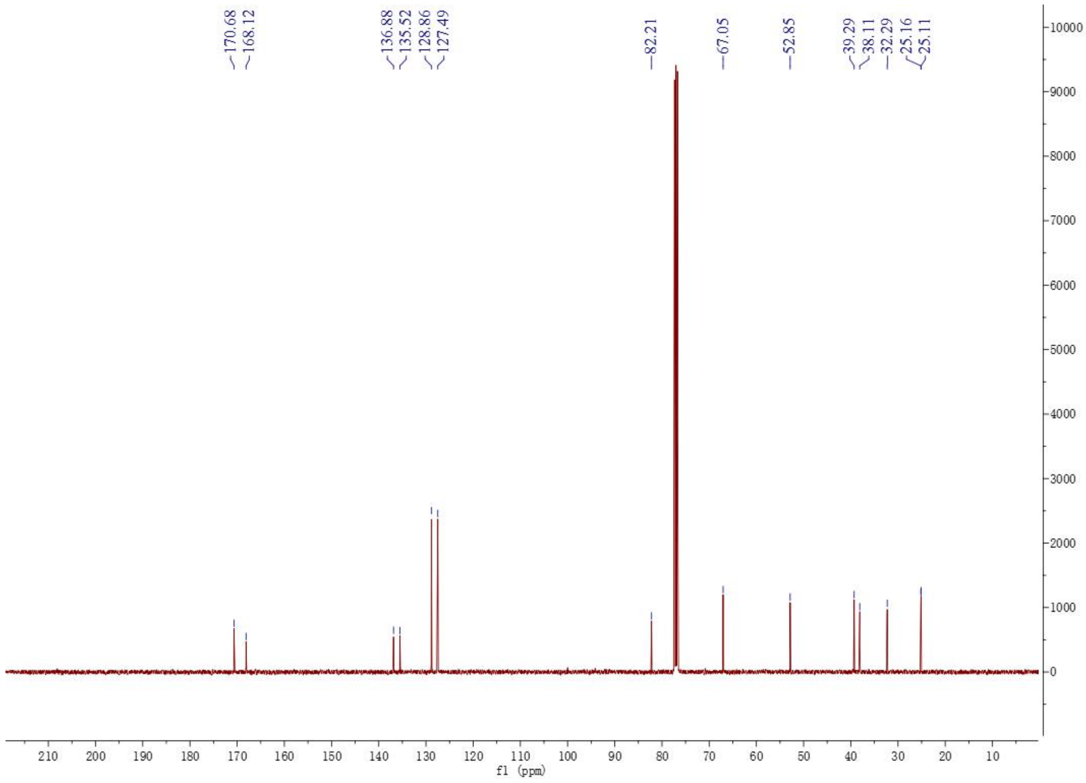

Figure S28. ^{13}C NMR spectrum of compound **4g** in $CDCl_3$.

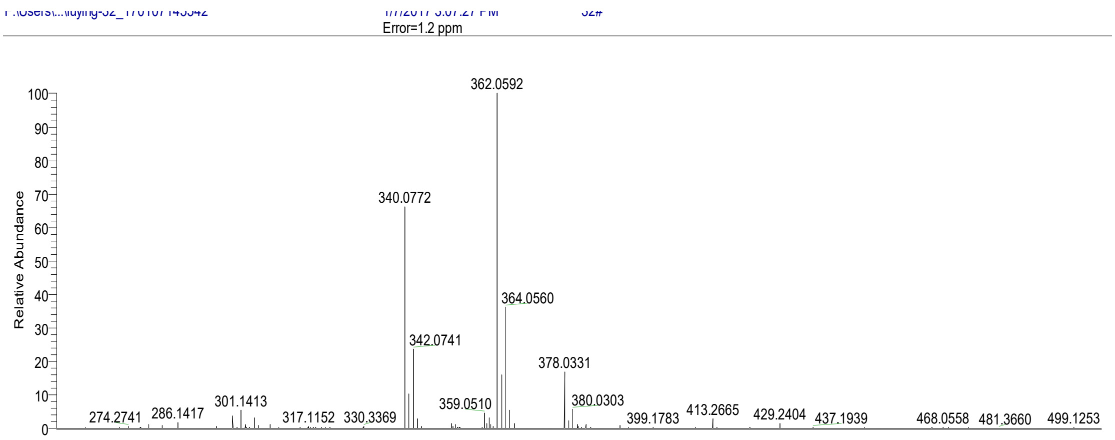

Figure S29. HMRS spectrum of compound **4g**.

Methyl (R)-4-(*p*-nitrobenzoyl)-1-thia-4-azaspiro[4.4]nonane-3-carboxylate (4h).

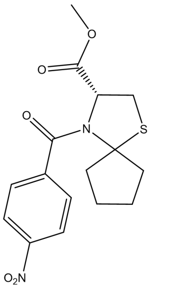

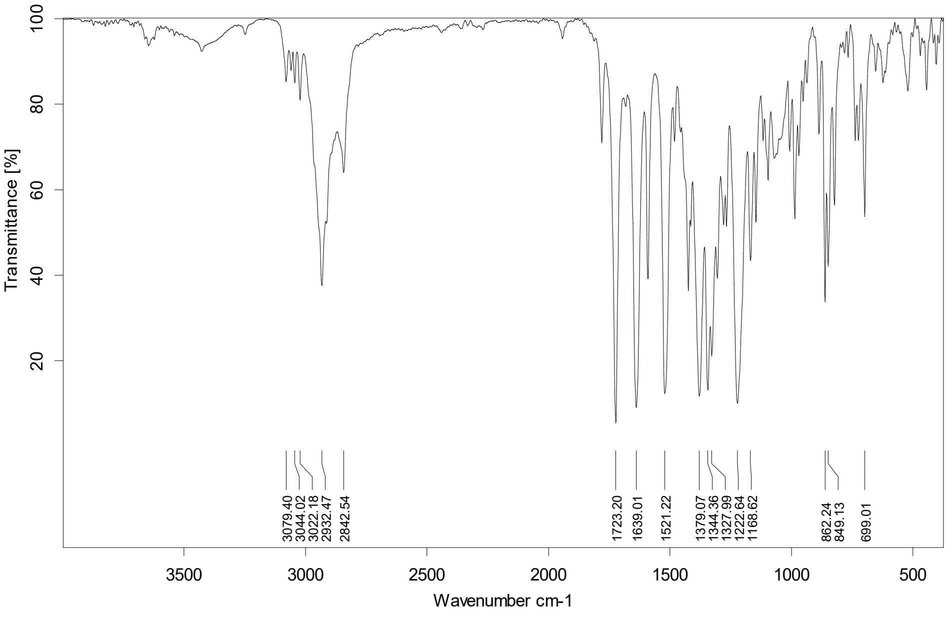

Figure S30. IR spectrum of compound **4h**.

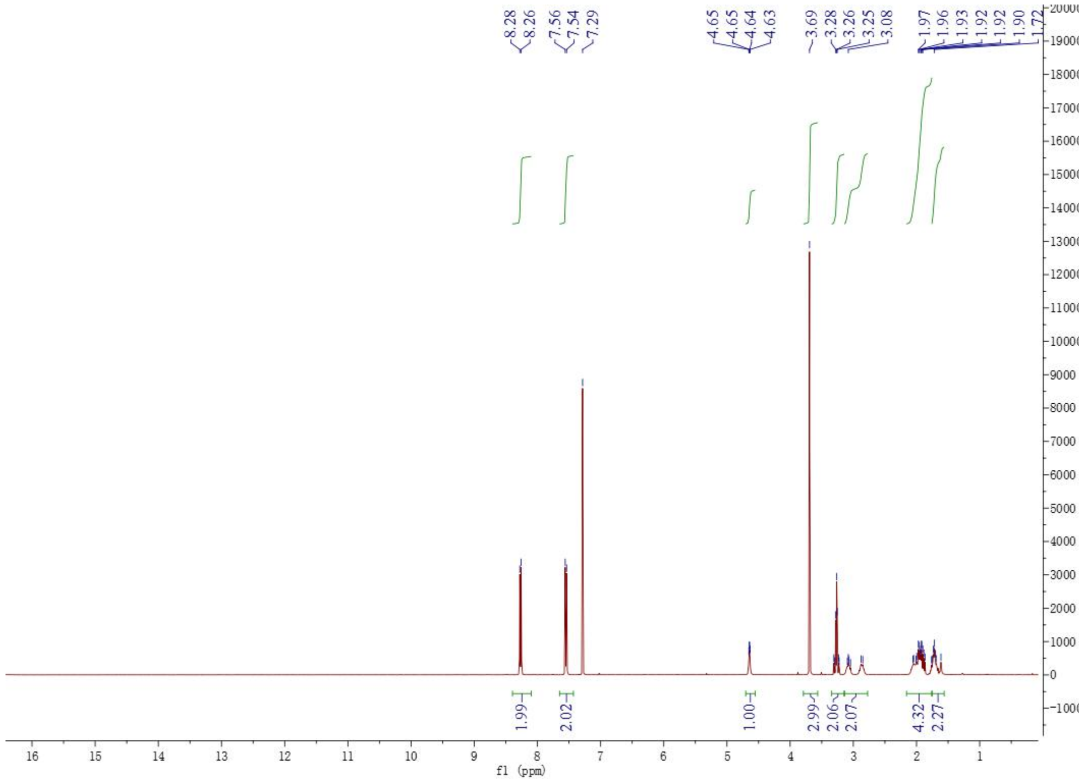

Figure S31. ^{1}H NMR spectrum of compound **4h**.

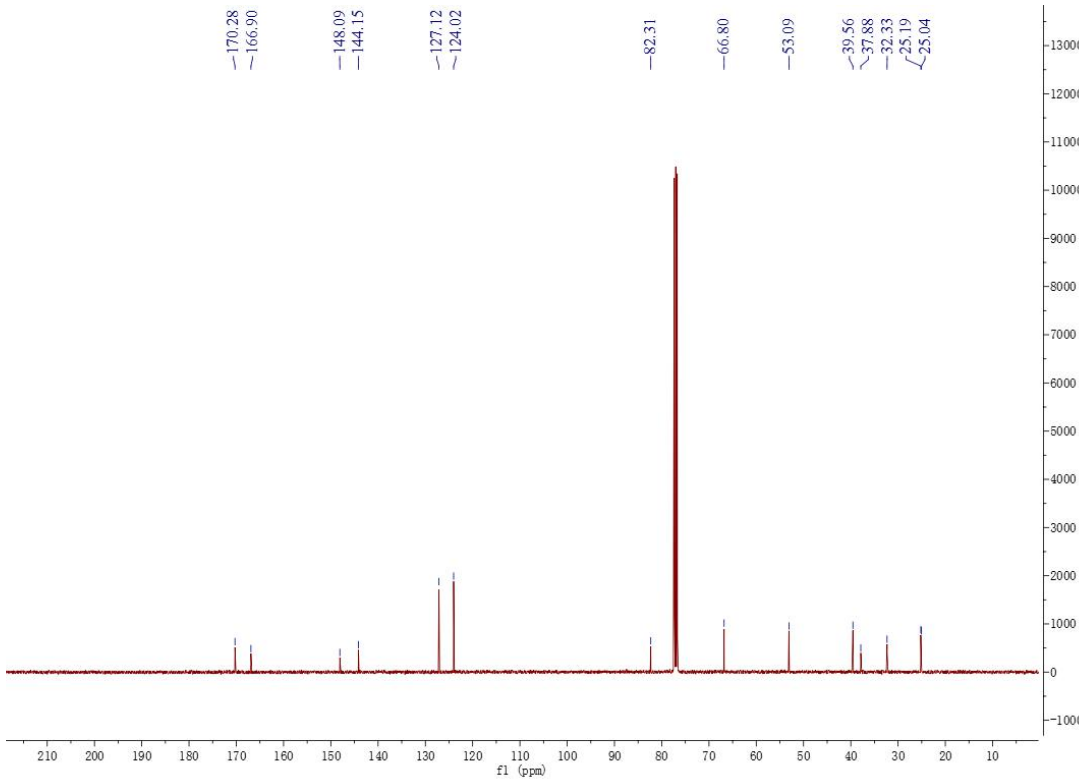

Figure S32. ^{13}C NMR spectrum of compound **4h** in $CDCl_3$.

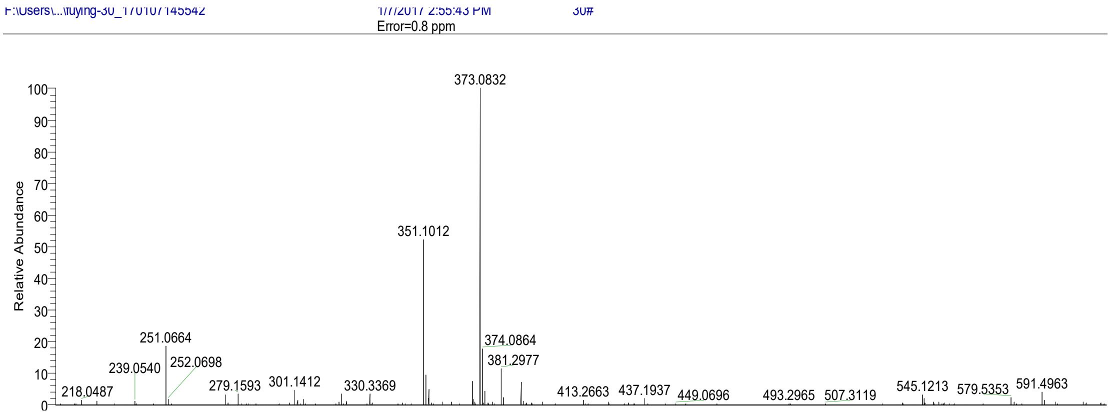

Figure S33. HMRS spectrum of compound **4h**.

Methyl (R)-4-(2,4-dichlorobenzoyl)-1-thia-4-azaspiro[4.4]nonane-3-carboxylate (4i).

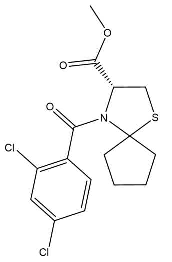

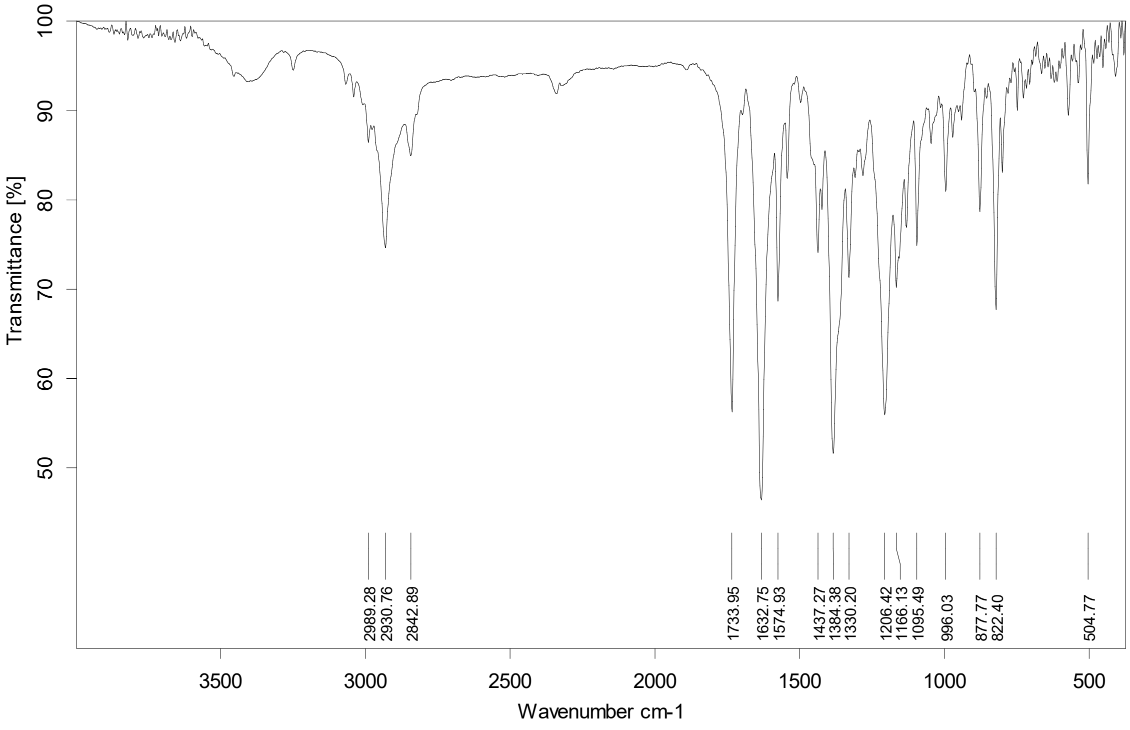

Figure S34. IR spectrum of compound **4i**.

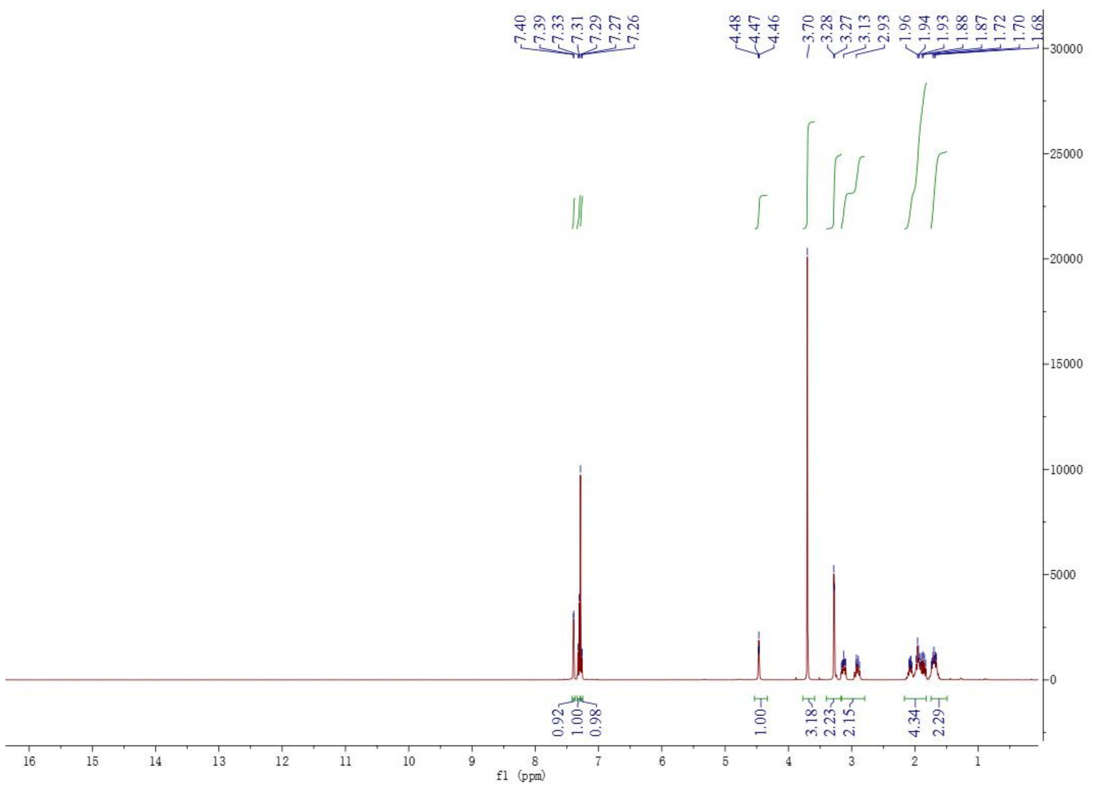

Figure S35. ^{1}H NMR spectrum of compound **4i**.

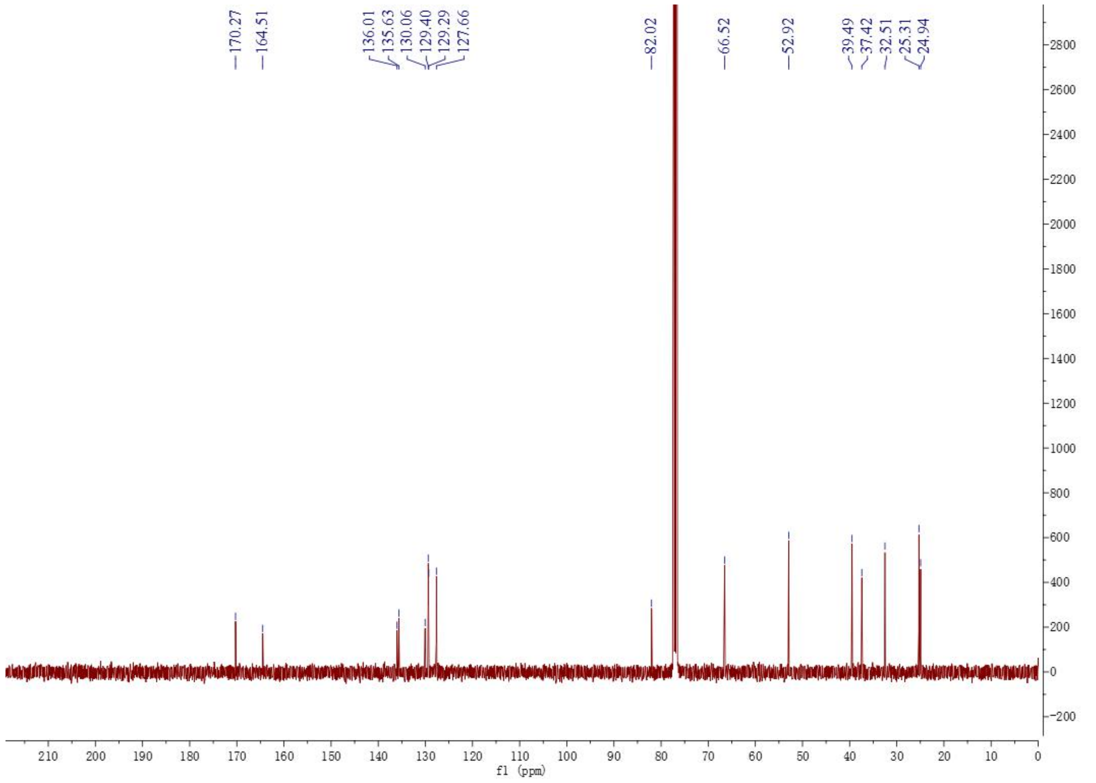

Figure S36. ^{13}C NMR spectrum of compound **4i** in $CDCl_3$.

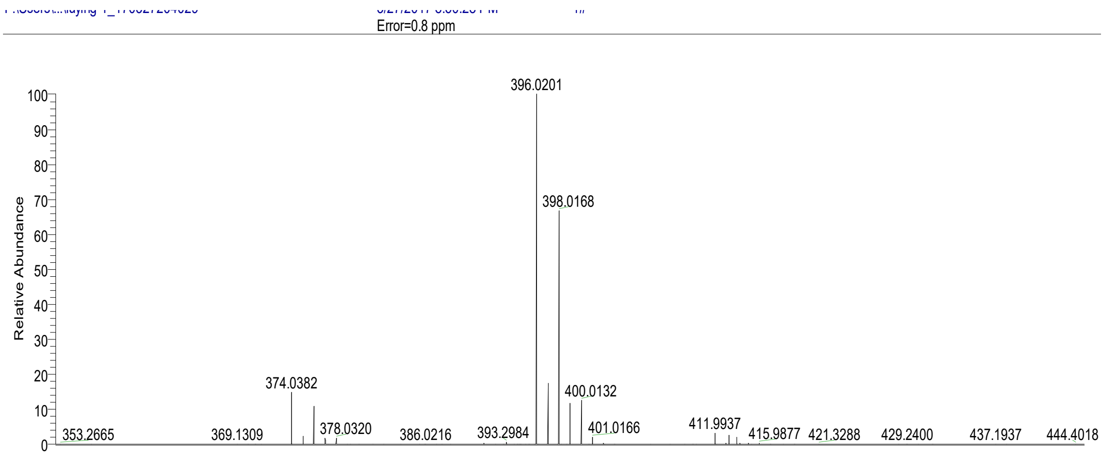

Figure S37. HMRS spectrum of compound **4i**.

Methyl (R)-4-(*m*-methylbenzoyl)-1-thia-4-azaspiro[4.4]nonane-3-carboxylate (4j).

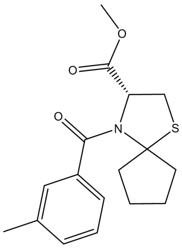

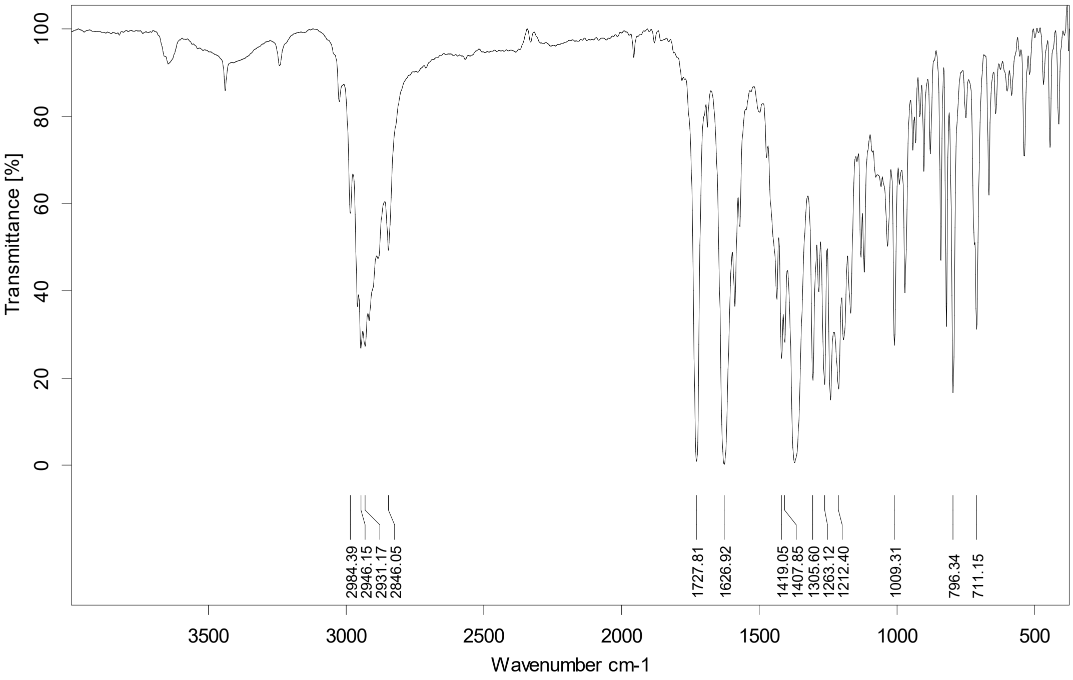

Figure S38. IR spectrum of compound **4j**.

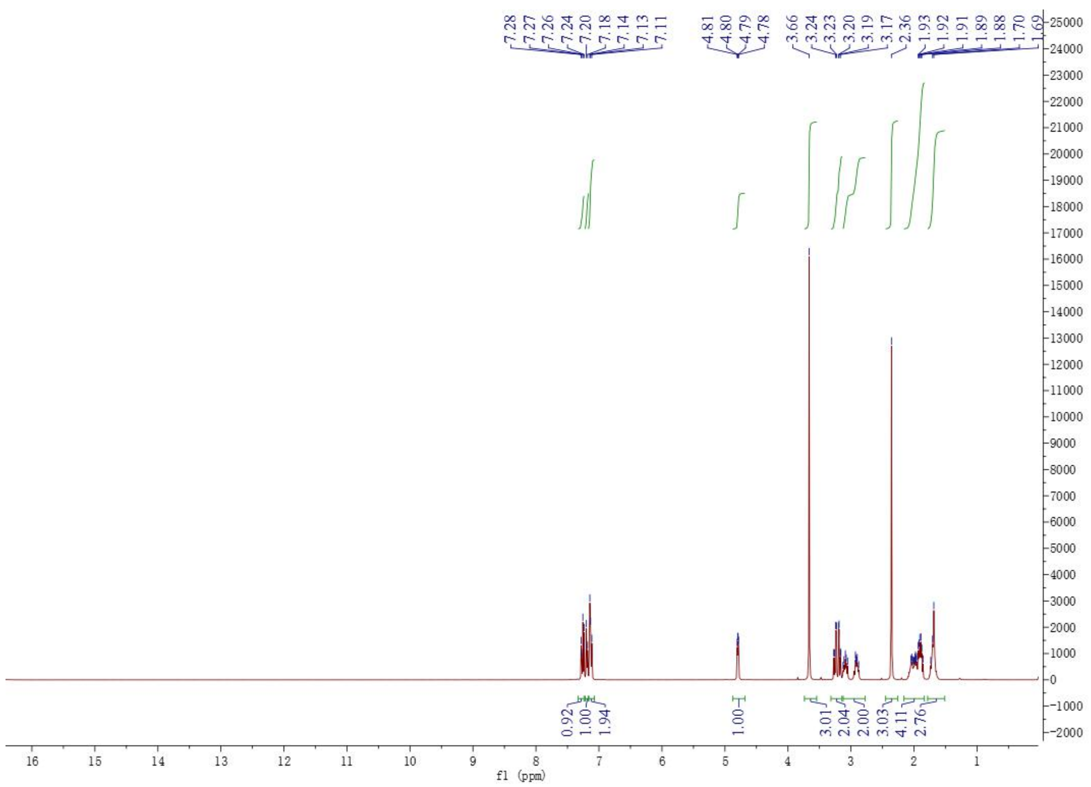

Figure S39. ^{1}H NMR spectrum of compound **4j**.

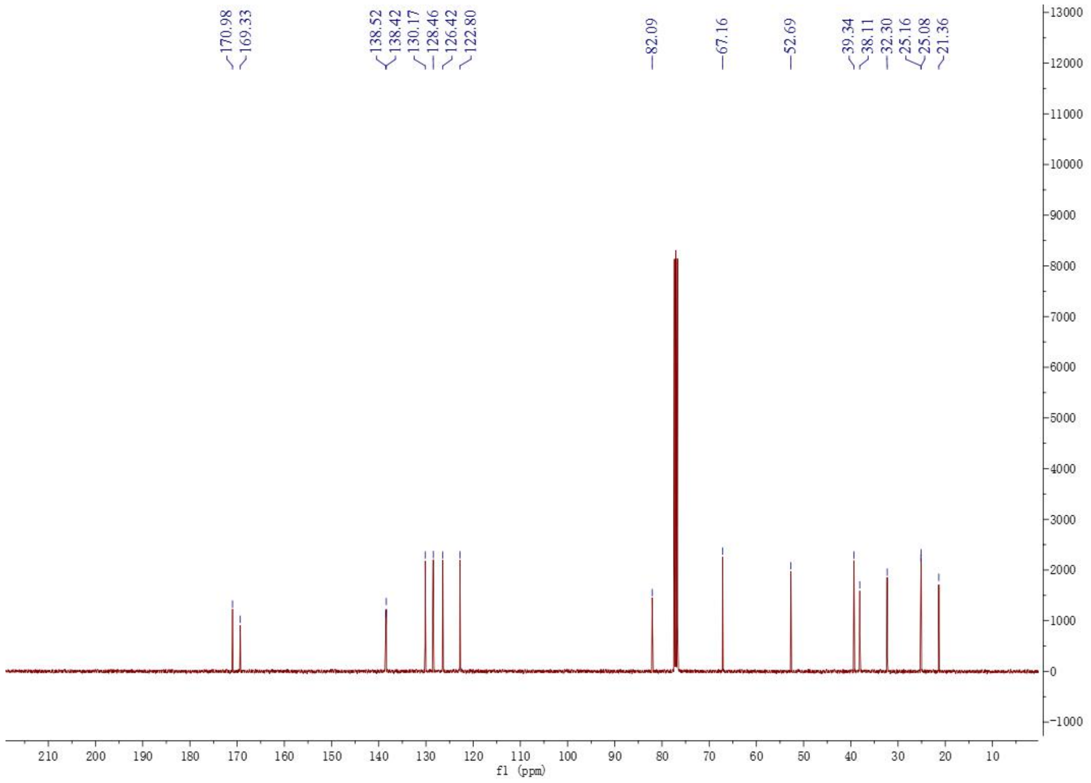

Figure S40. ^{13}C NMR spectrum of compound **4j** in $CDCl_3$.

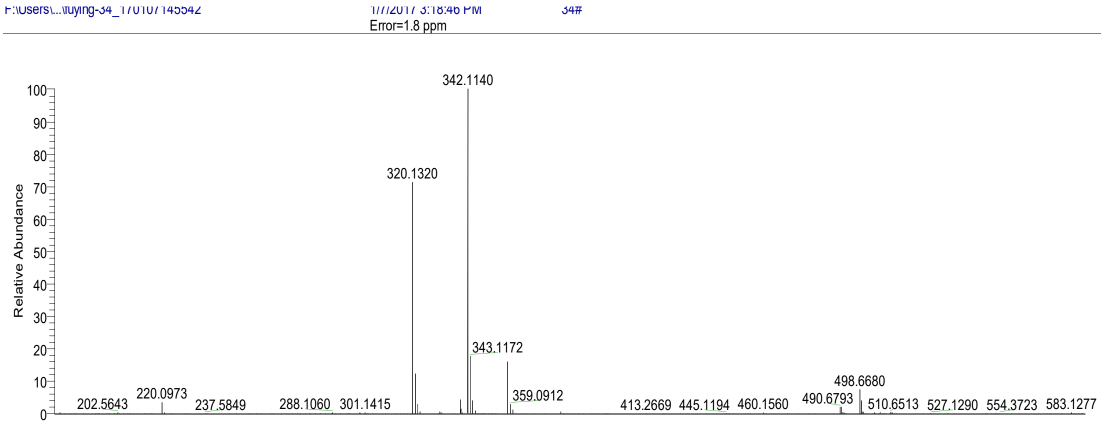

Figure S41. HMRS spectrum of compound **4j**.

Methyl (R)-4-(*o*-chlorobenzoyl)-1-thia-4-azaspiro[4.4]nonane-3-carboxylate (4k).

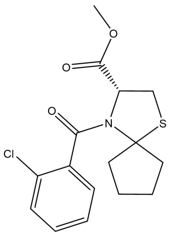

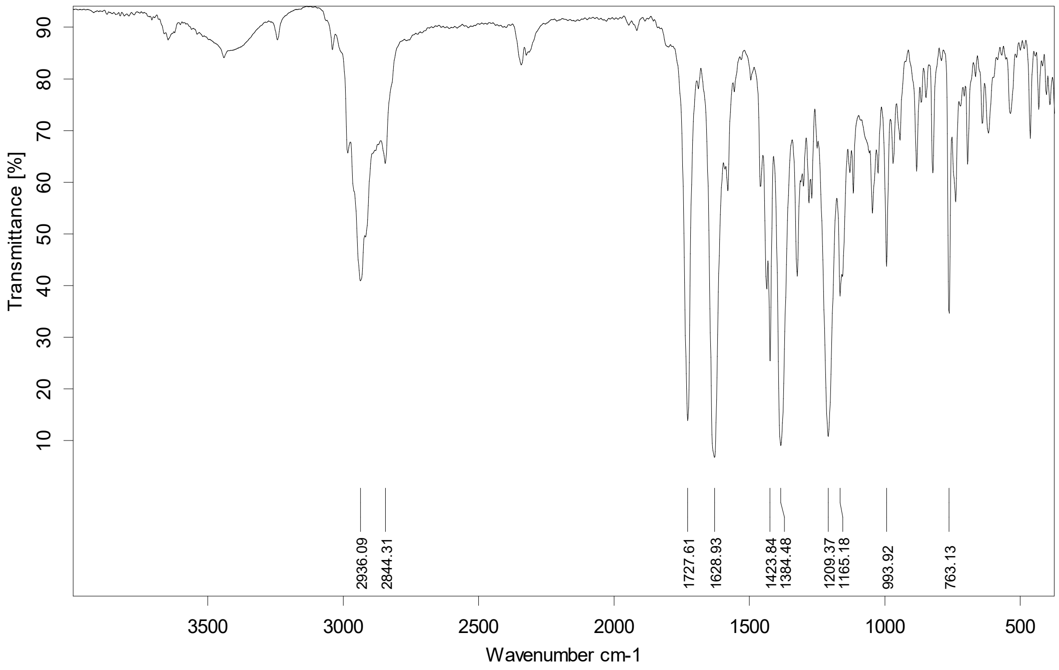

Figure S42. IR spectrum of compound **4k**.

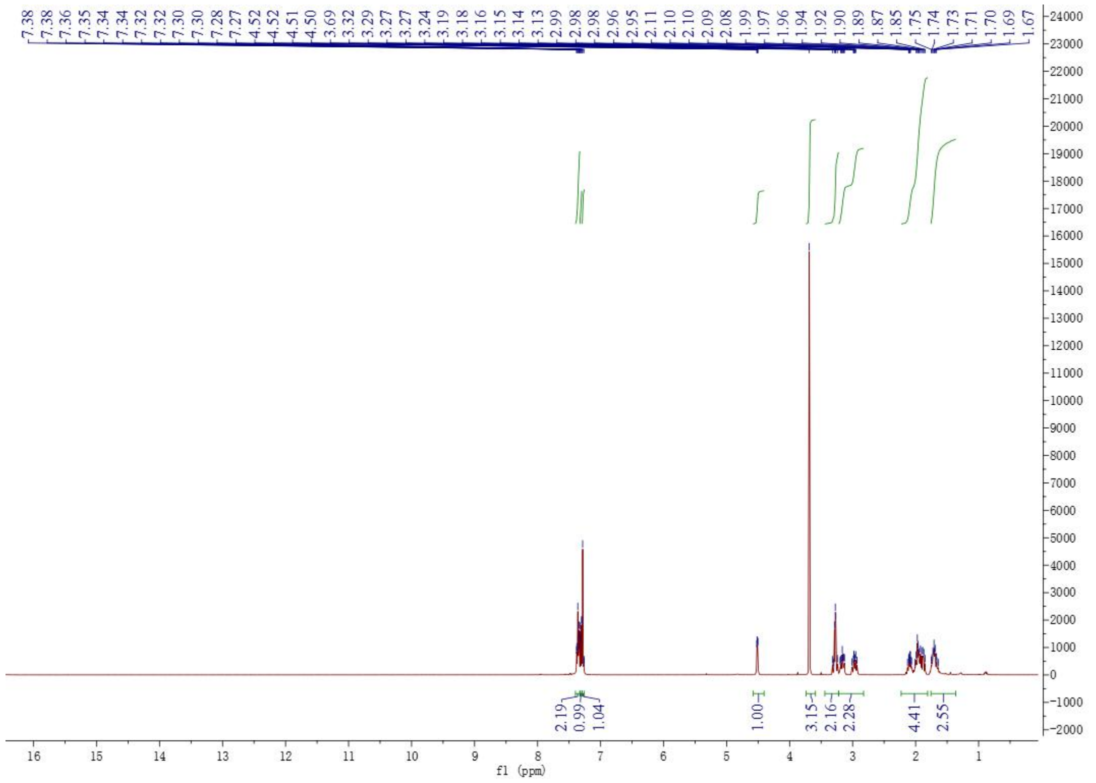

Figure S43. ^{1}H NMR spectrum of compound **4k**.

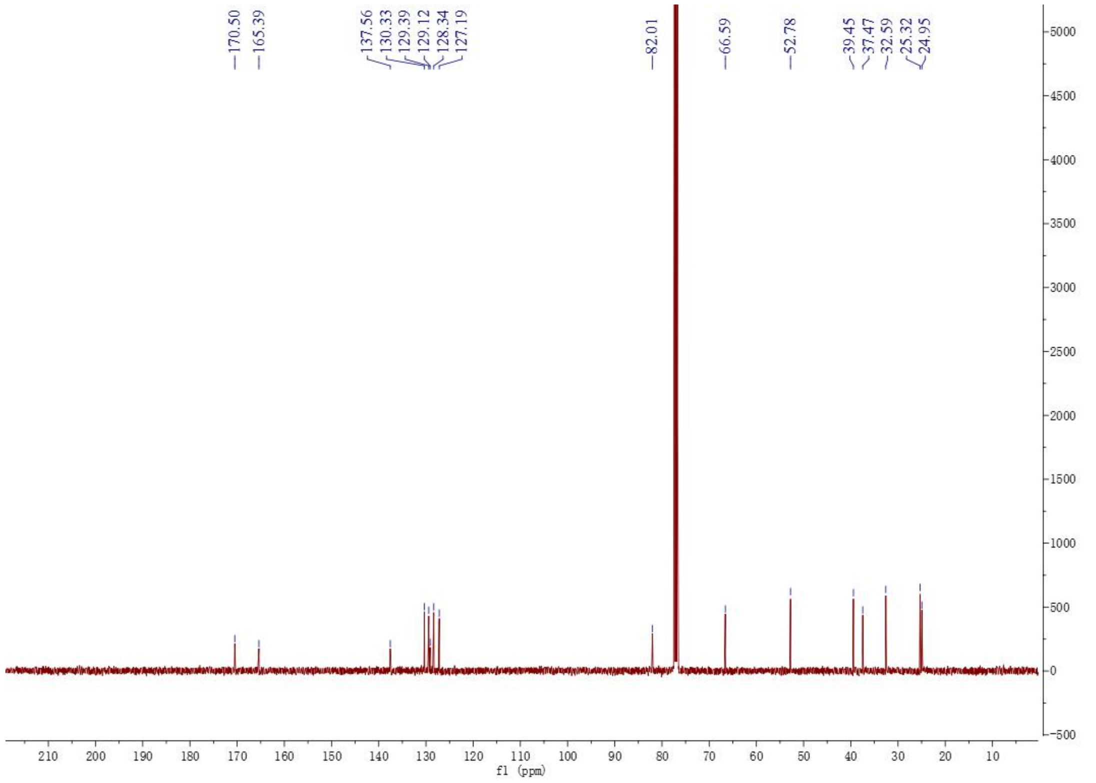

Figure S44. ^{13}C NMR spectrum of compound **4k** in $CDCl_3$.

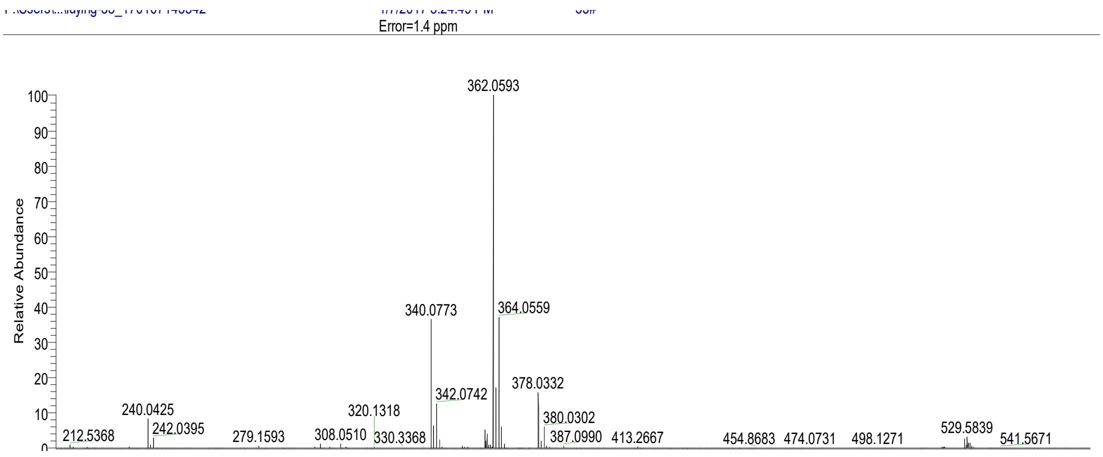

Figure S45. HMRS spectrum of compound **4k**.

Methyl (R)-3-(2,4-dichlorobenzoyl)-2,2-dimethylthiazolidine-4-carboxylate (4l).

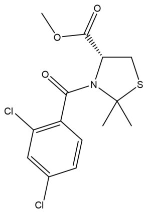

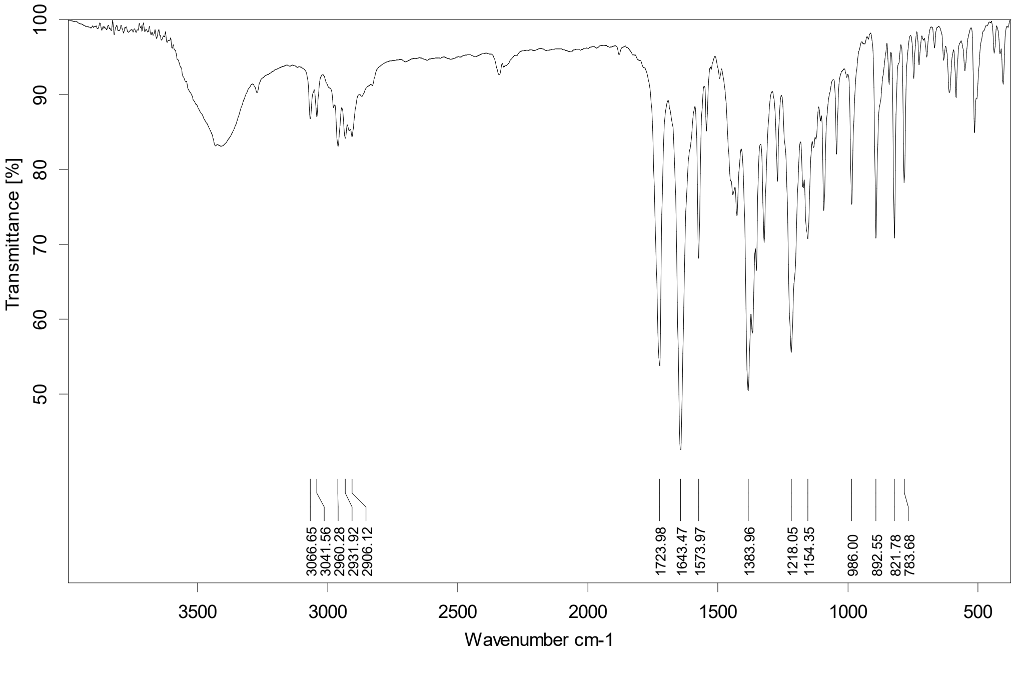

Figure S46. IR spectrum of compound **4l**.

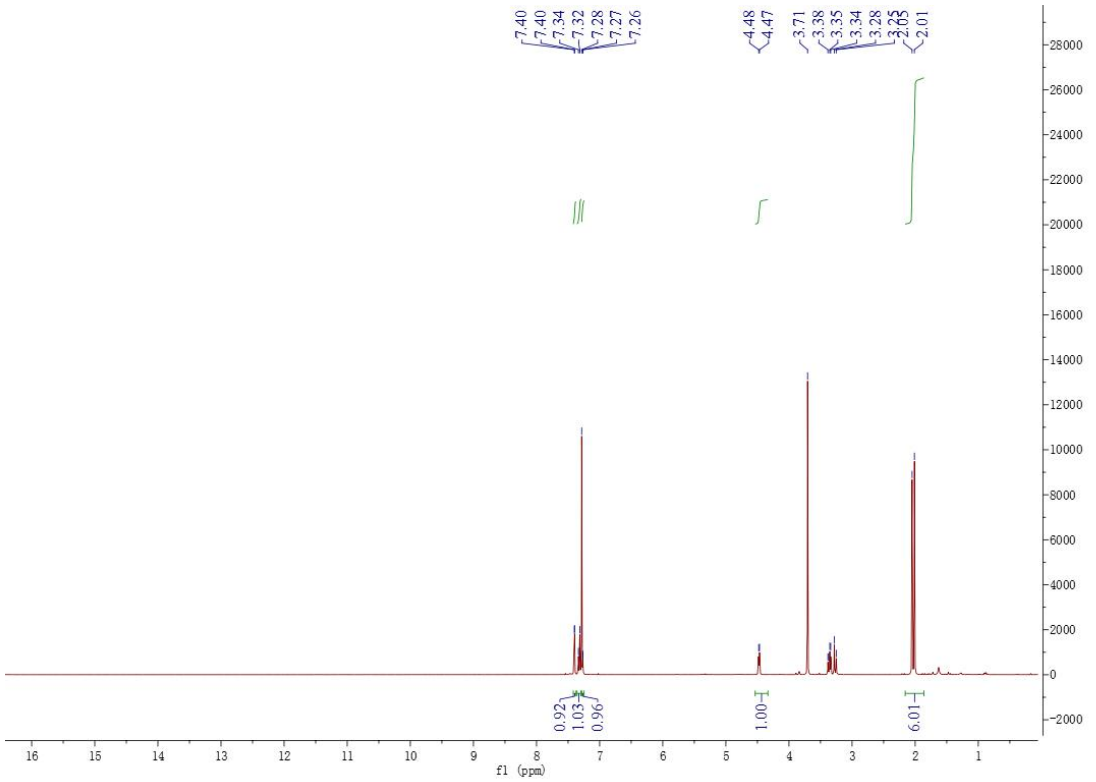

Figure S47. ^{1}H NMR spectrum of compound **4l**.

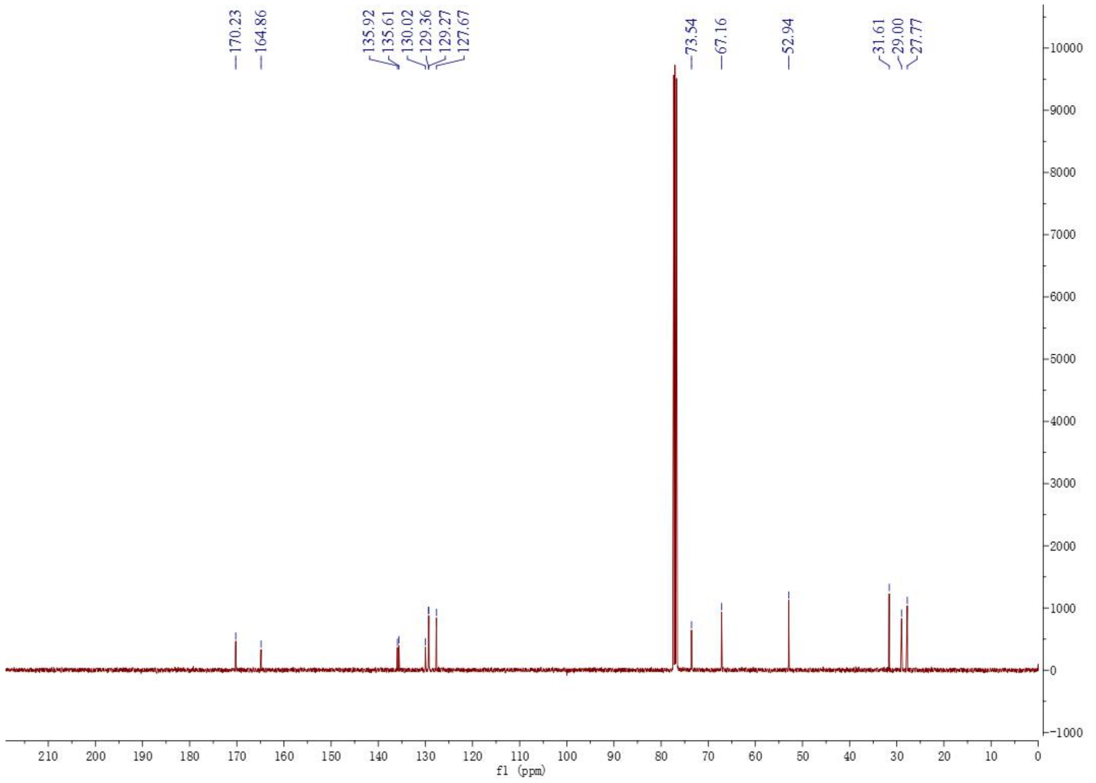

Figure S48. ^{13}C NMR spectrum of compound **4l** in $CDCl_3$.

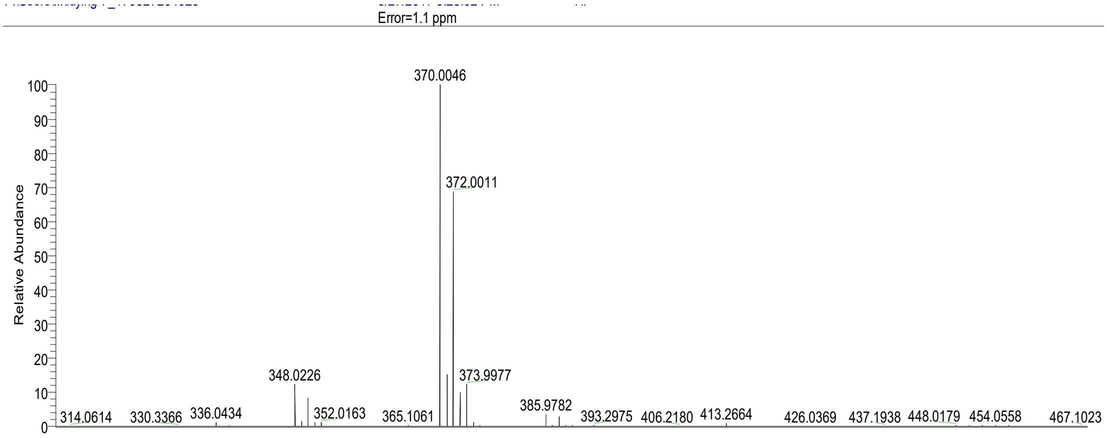

Figure S49. HMRS spectrum of compound **4l**.

Methyl (R)-3-(*p*-chlorobenzoyl)-2,2-dimethylthiazolidine-4-carboxylate (4m).

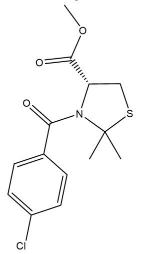

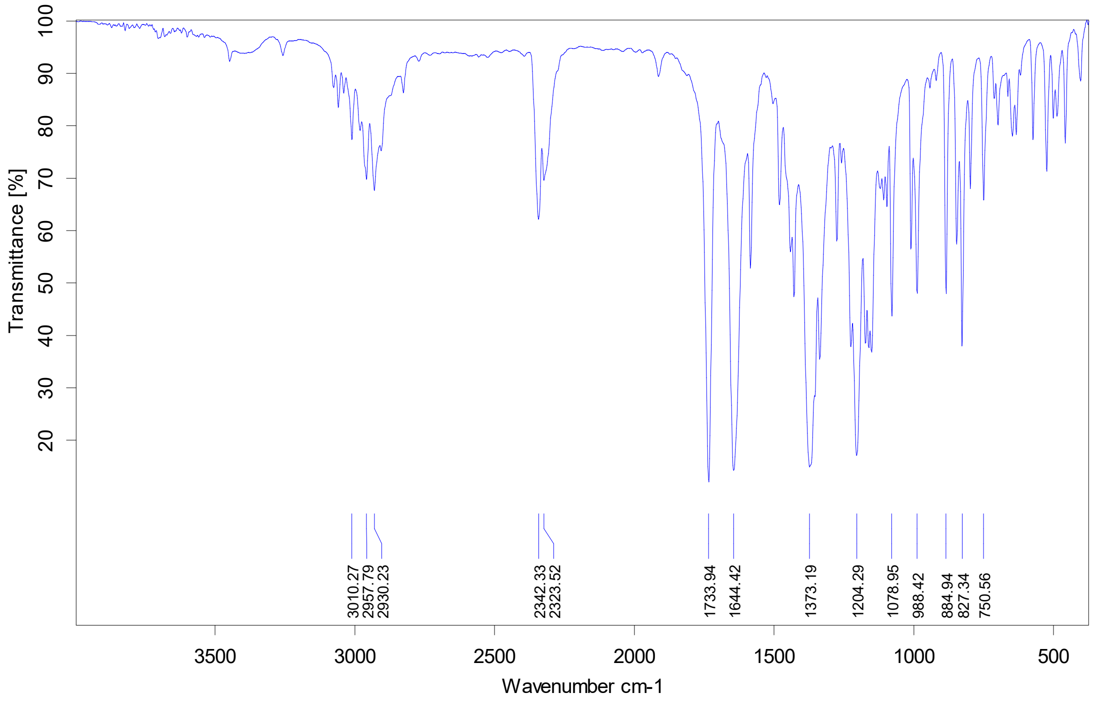

Figure S50. IR spectrum of compound **4m**.

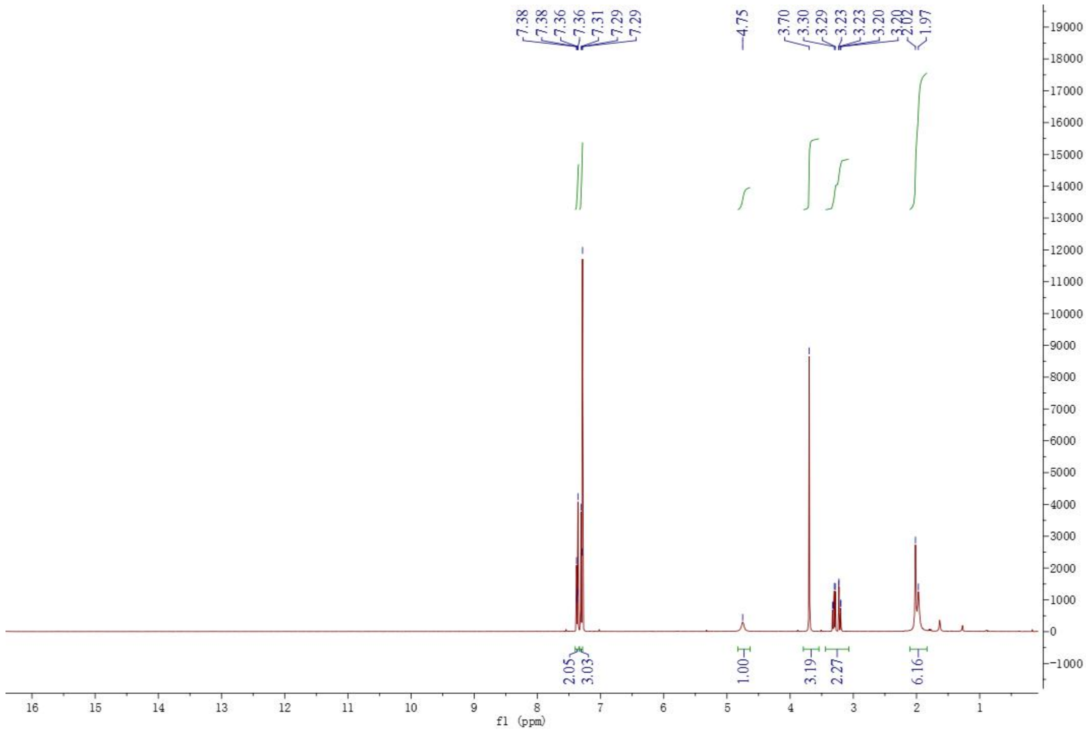

Figure S51. ^{1}H NMR spectrum of compound **4m**.

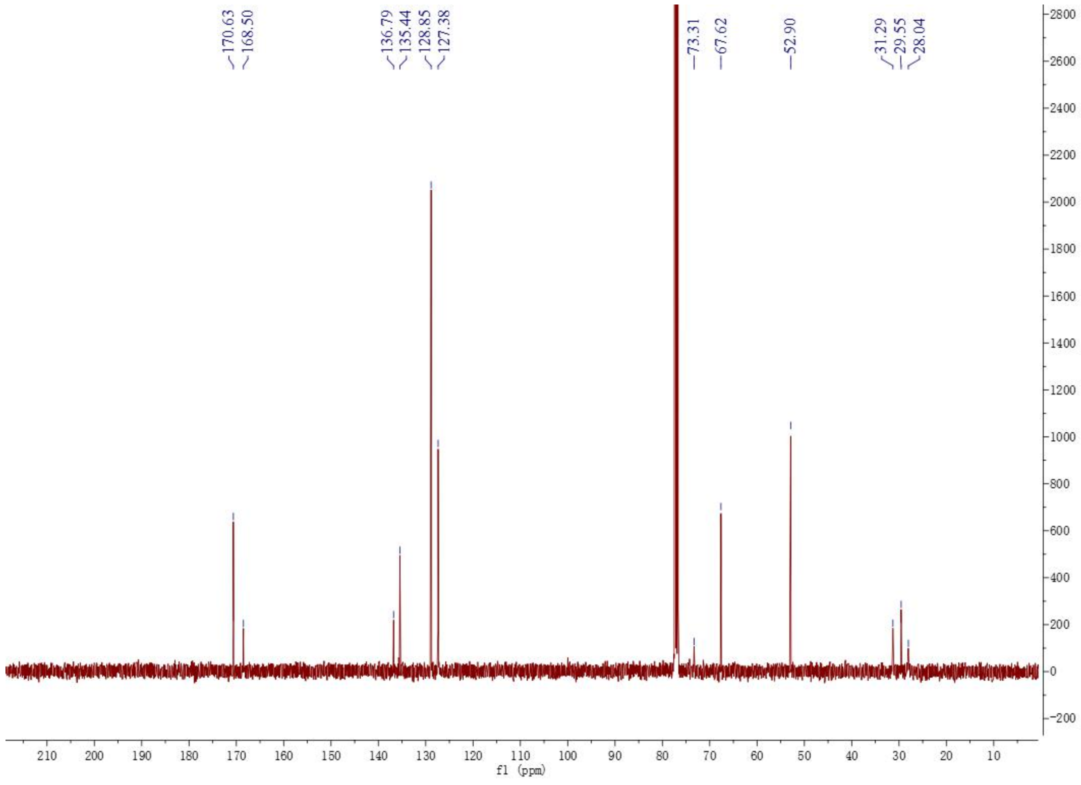

Figure S52. ^{13}C NMR spectrum of compound **4m** in $CDCl_3$.

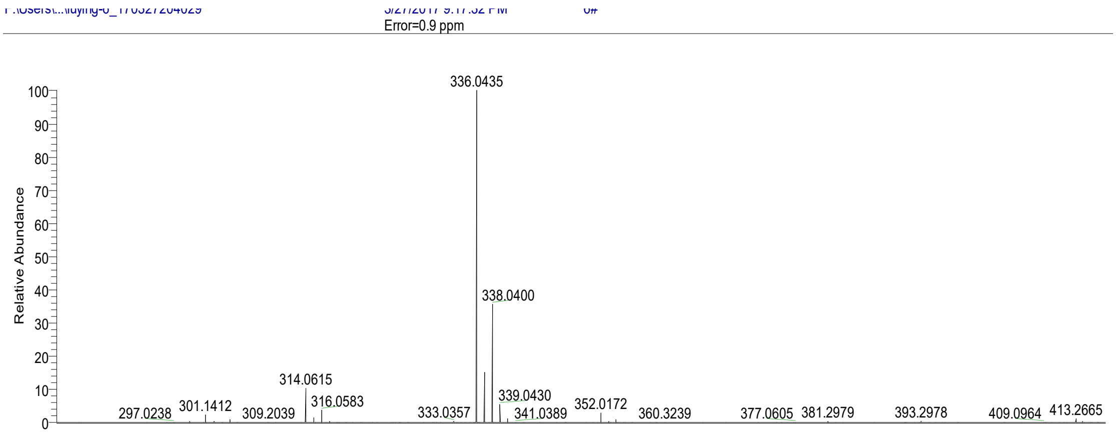

Figure S53. HMRS spectrum of compound **4m**.

Methyl (R)-2,2-dimethyl-3-(*p*-nitrobenzoyl)thiazolidine-4-carboxylate (4n).

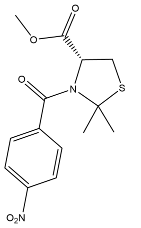

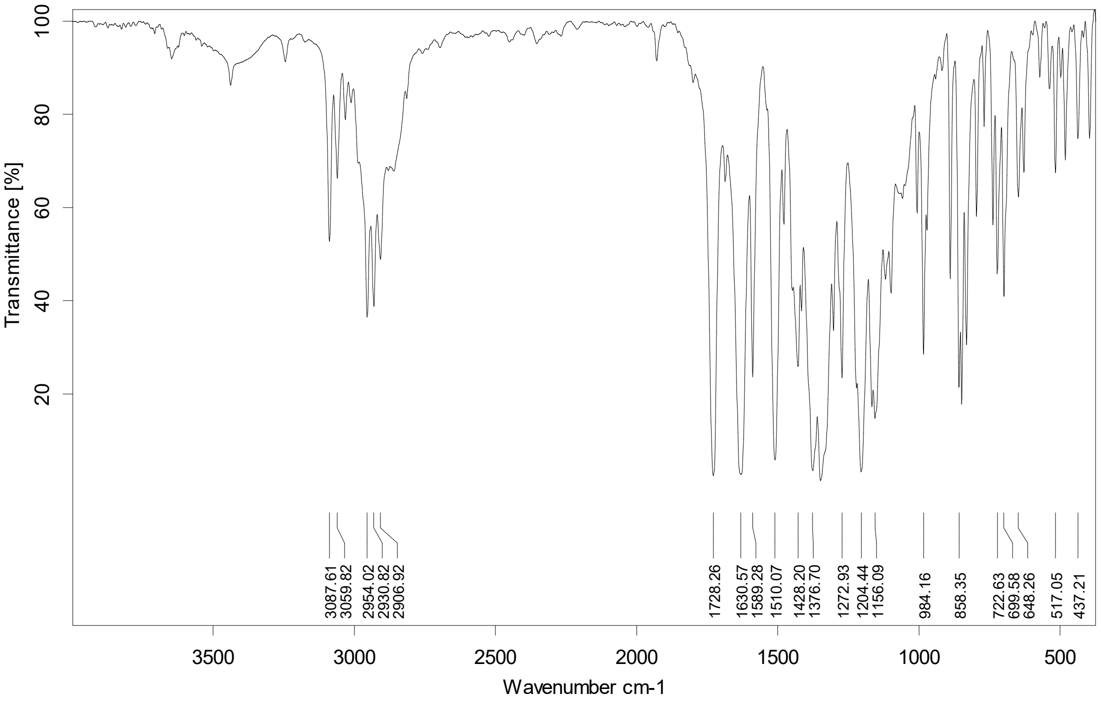

Figure S54. IR spectrum of compound **4n**.

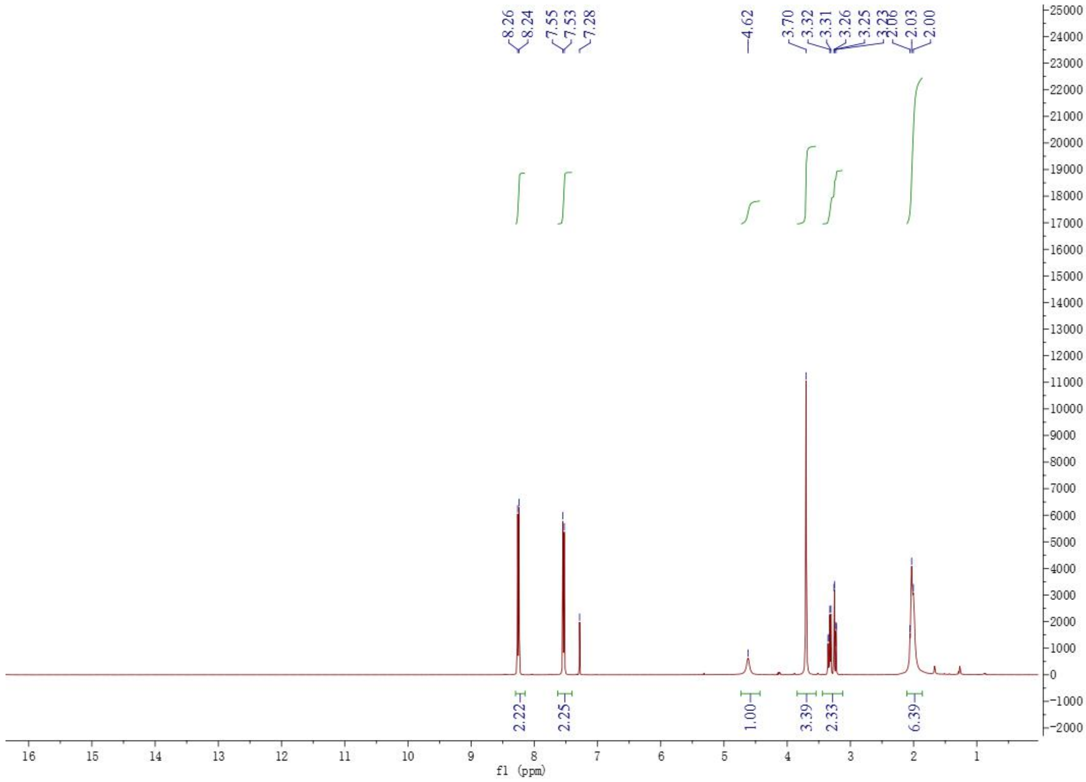

Figure S55. ^{1}H NMR spectrum of compound **4n**.

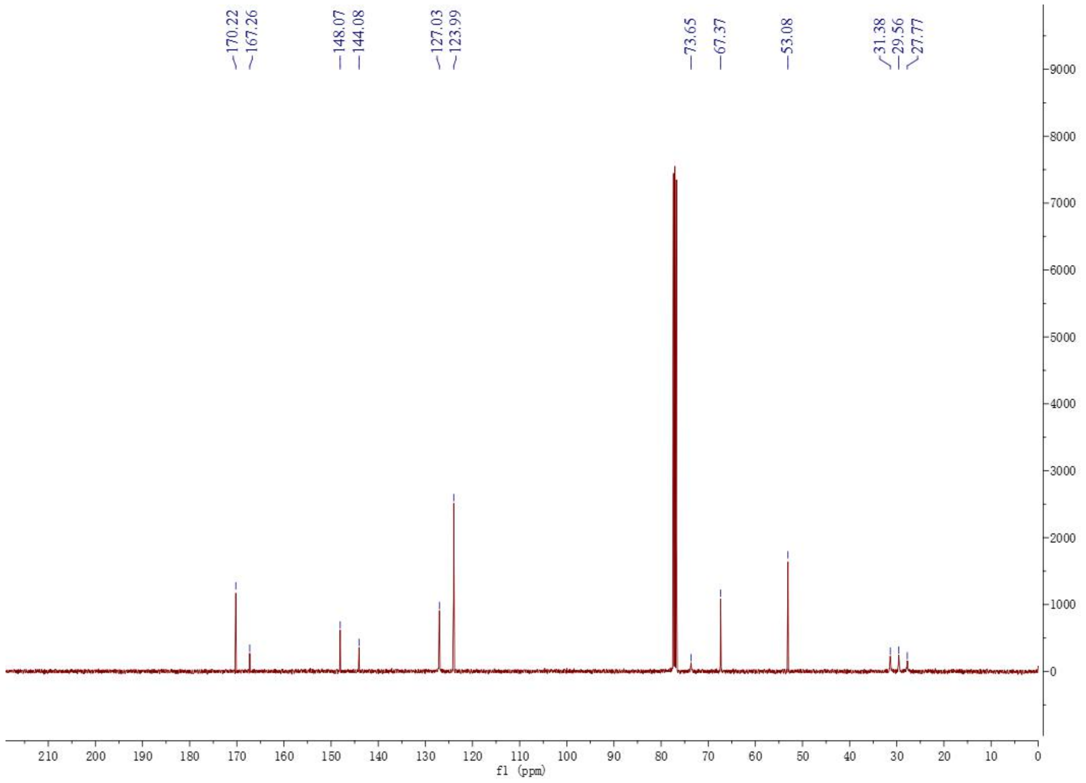

Figure S56. ^{13}C NMR spectrum of compound **4n** in $CDCl_3$.

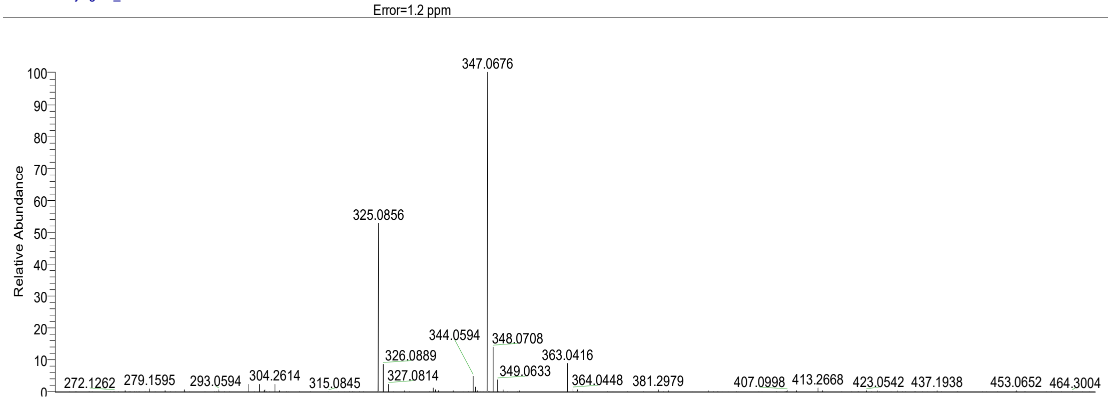

Figure S57. HMRS spectrum of compound **4n**.

Methyl (R)-2,2-dimethyl-3-(*m*-methylbenzoyl)thiazolidine-4-carboxylate (4o).

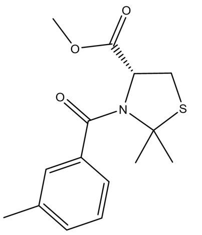

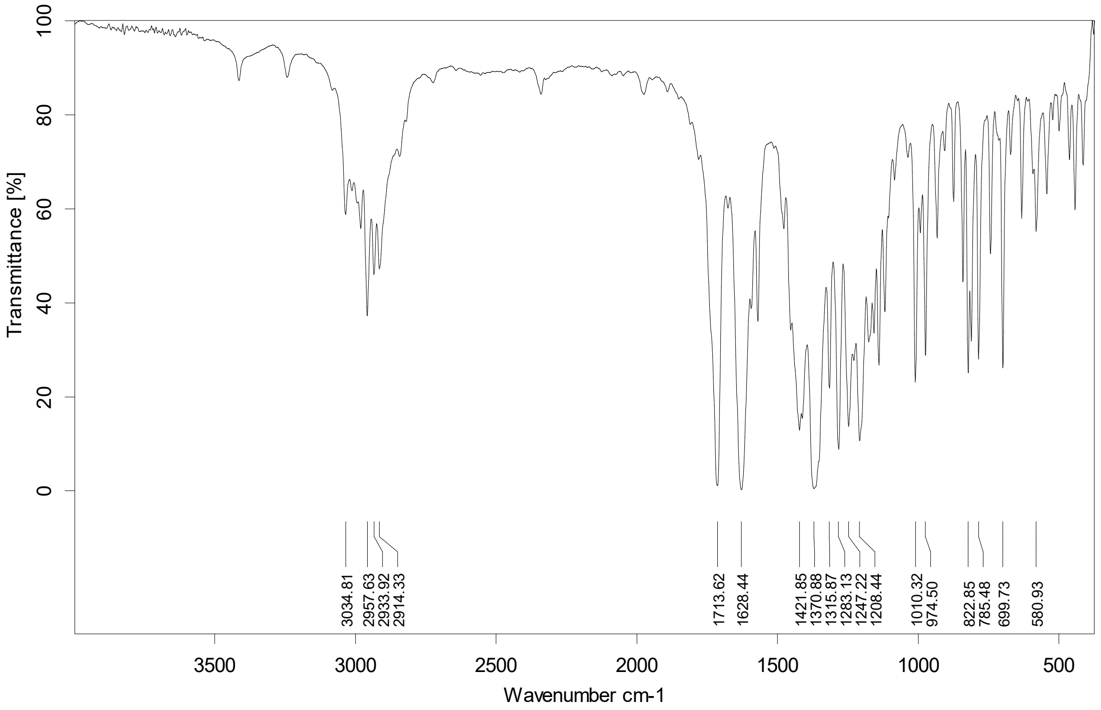

Figure S58. IR spectrum of compound **4o**.

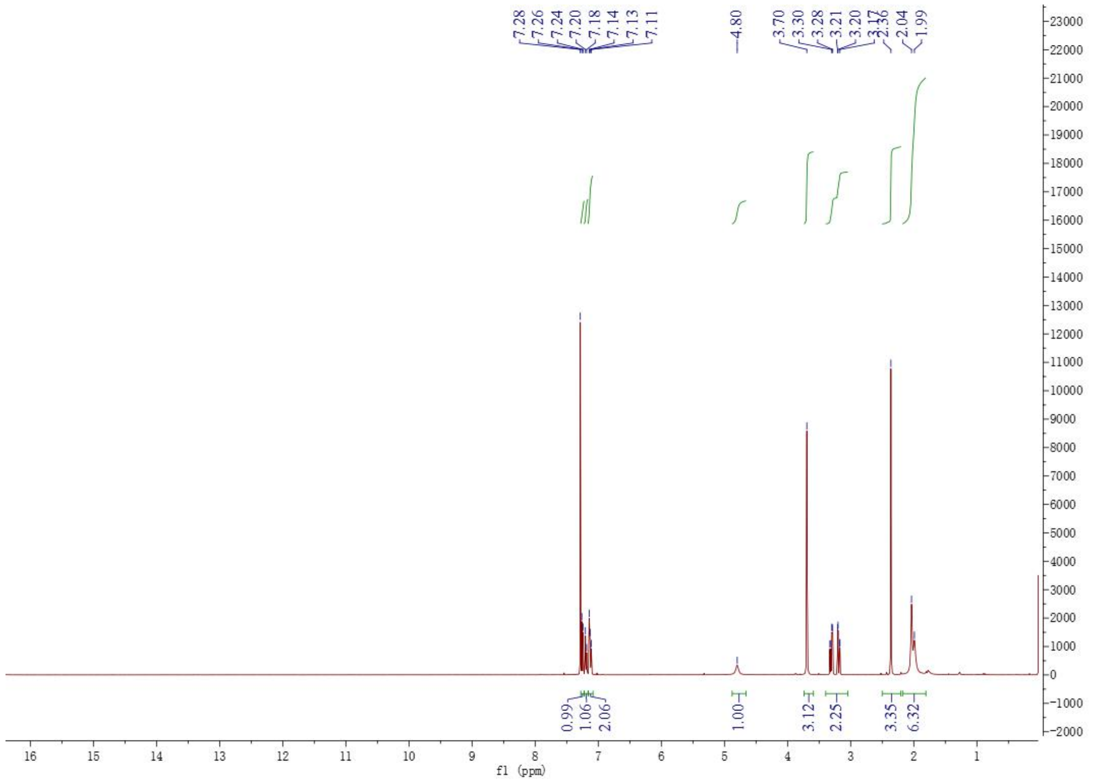

Figure S59. ^{1}H NMR spectrum of compound **4o**.

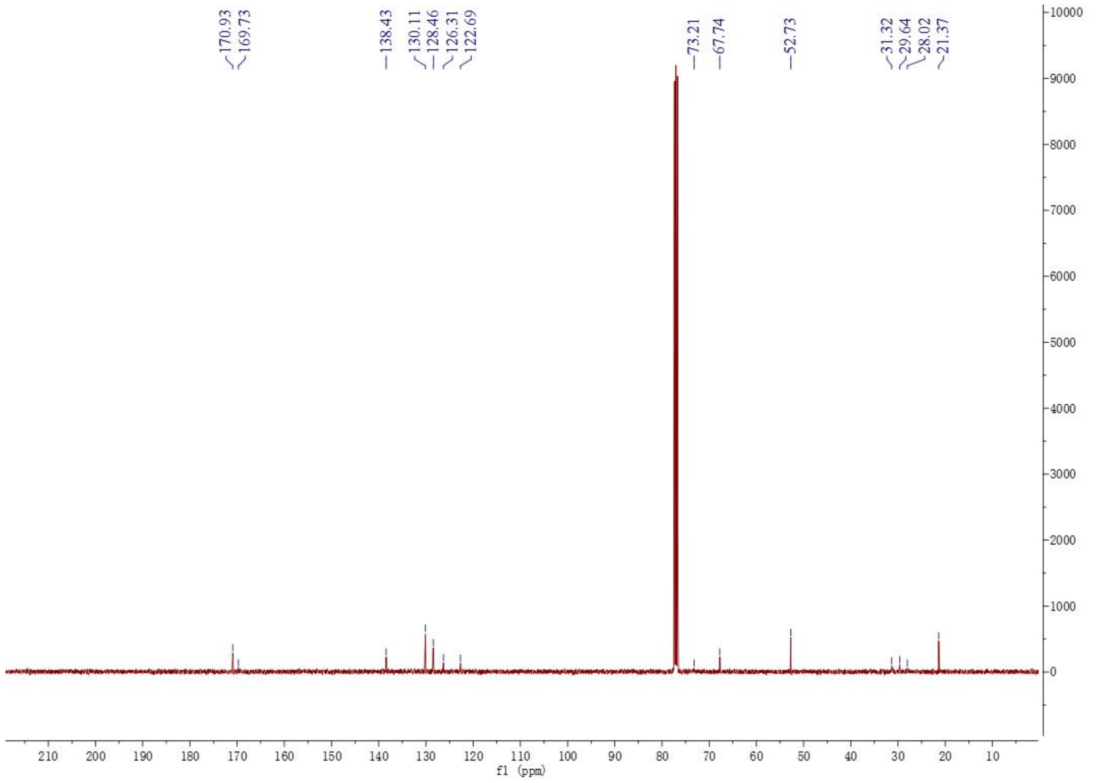

Figure S60. ^{13}C NMR spectrum of compound **4o** in $CDCl_3$.

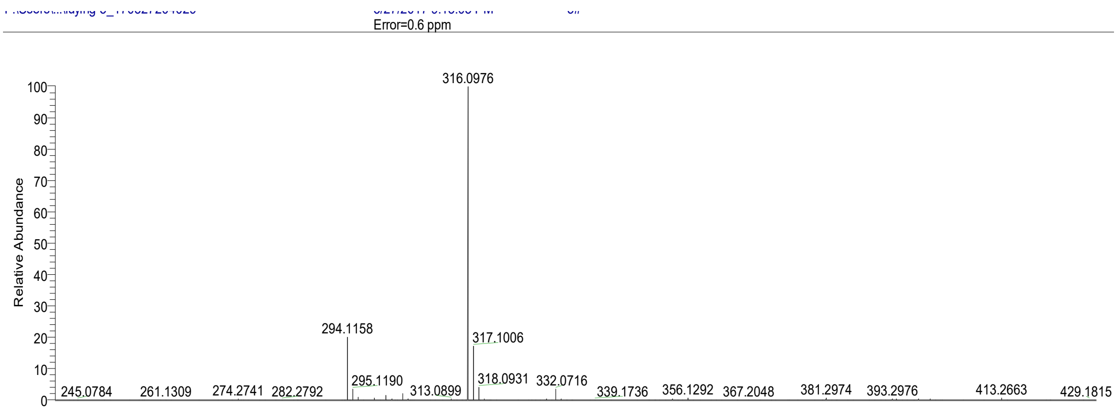

Figure S61. HMRS spectrum of compound **4o**.

Methyl (R)-3-(*o*-methoxybenzoyl)-2,2-dimethylthiazolidine-4-carboxylate (4p).

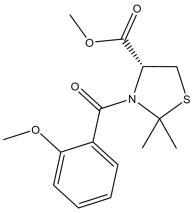

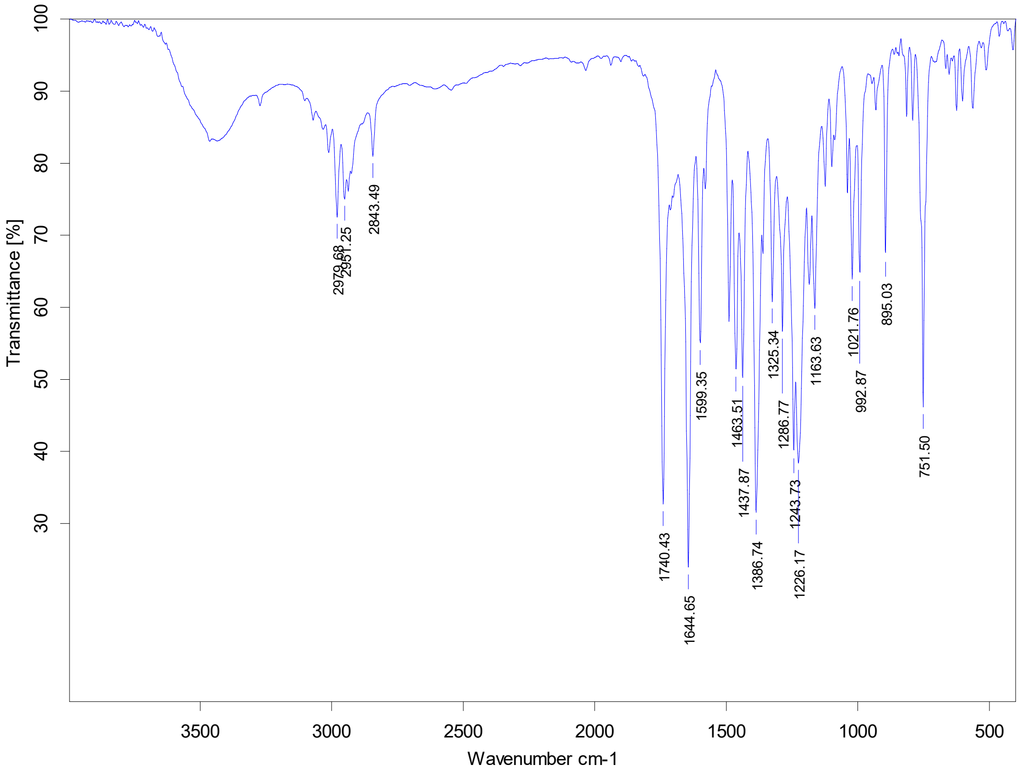

Figure S62. IR spectrum of compound **4p**.

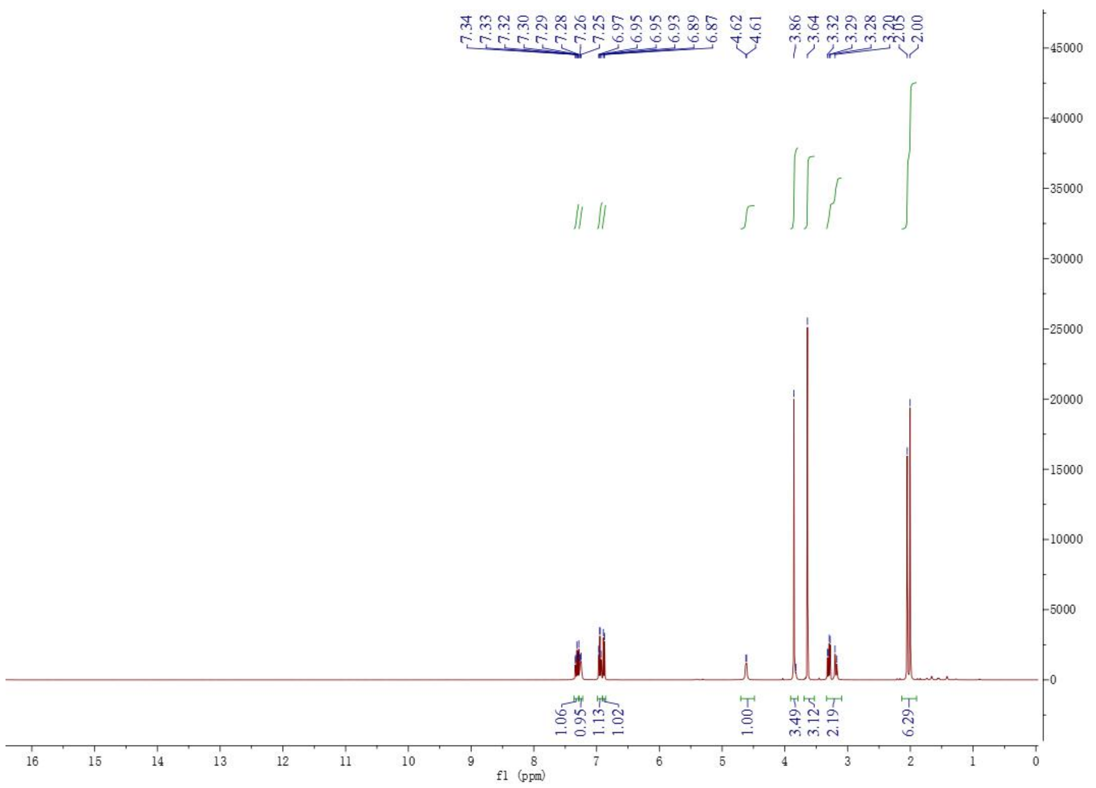

Figure S63. ^{1}H NMR spectrum of compound **4p**.

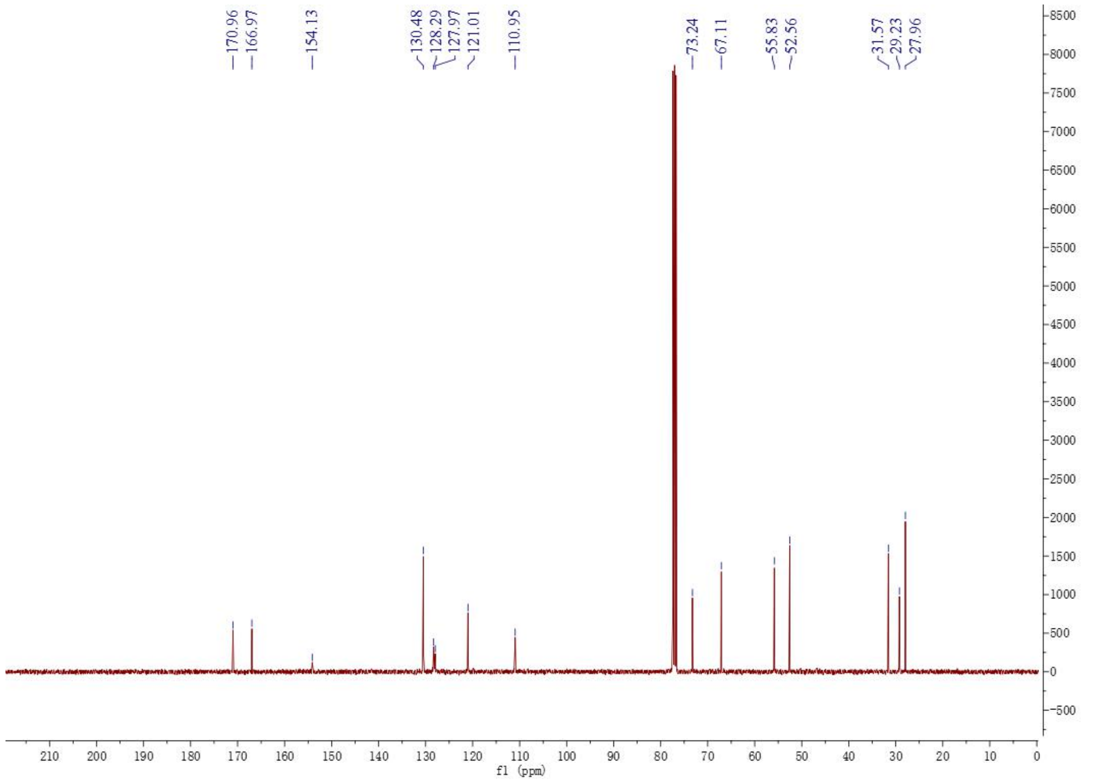

Figure S64. ^{13}C NMR spectrum of compound **4p** in $CDCl_3$.

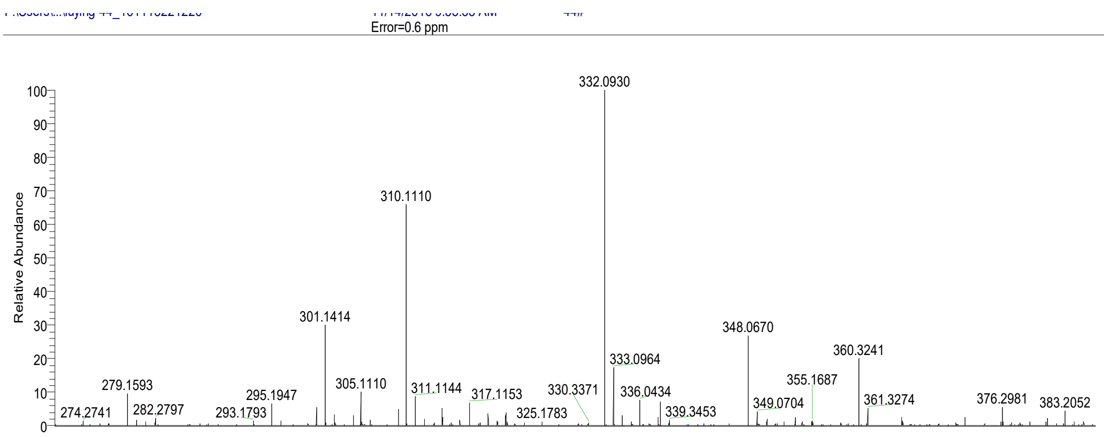

Figure S65. HMRS spectrum of compound **4p**.

Methyl (R)-3-(*o*-chlorobenzoyl)-2,2-dimethylthiazolidine-4-carboxylate (4q).

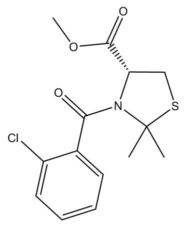

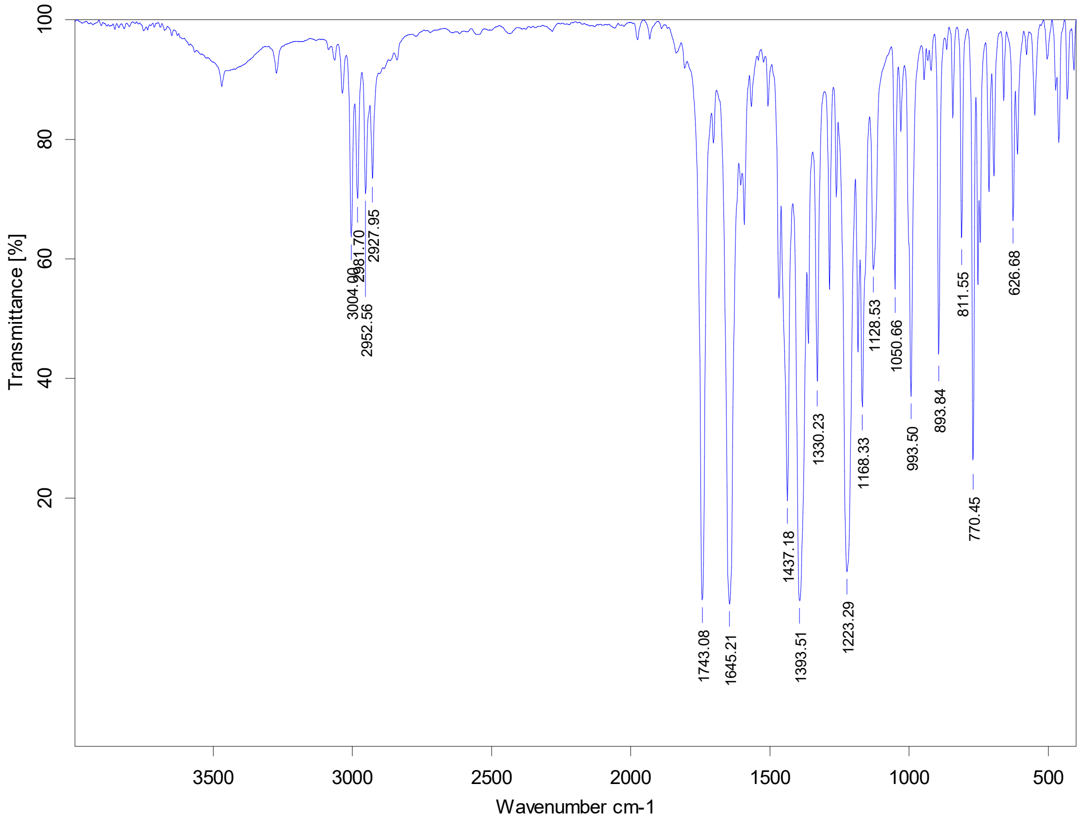

Figure S66. IR spectrum of compound **4q**.

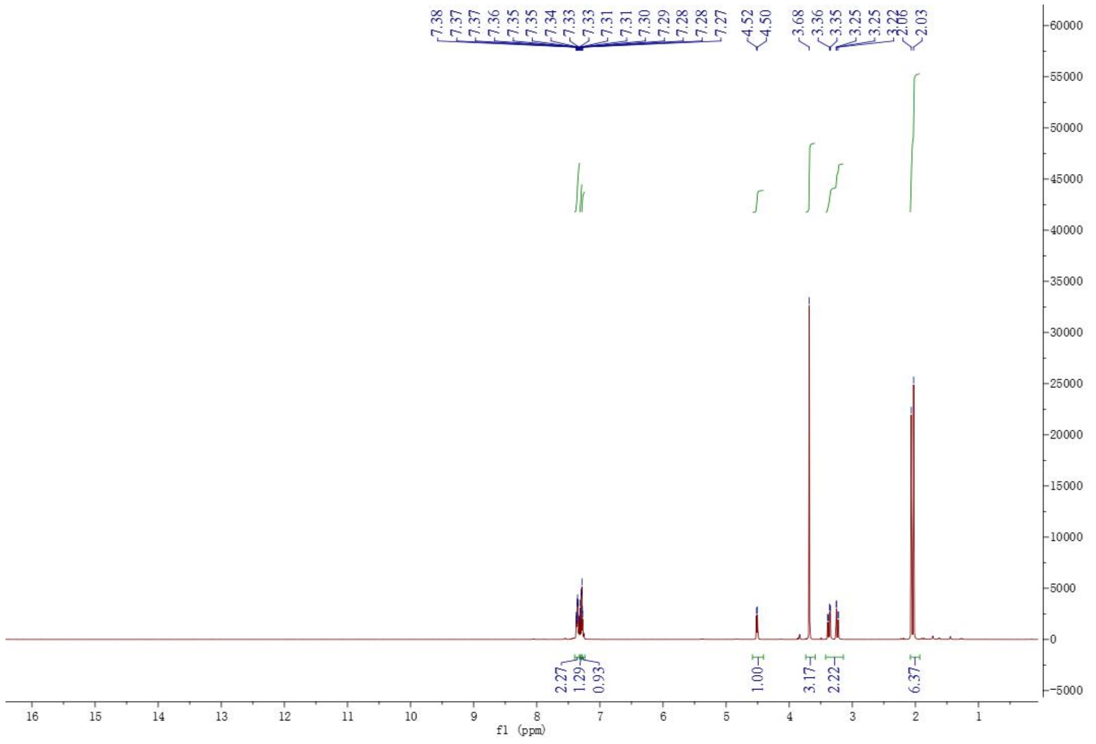

Figure S67. ^{1}H NMR spectrum of compound **4q**.

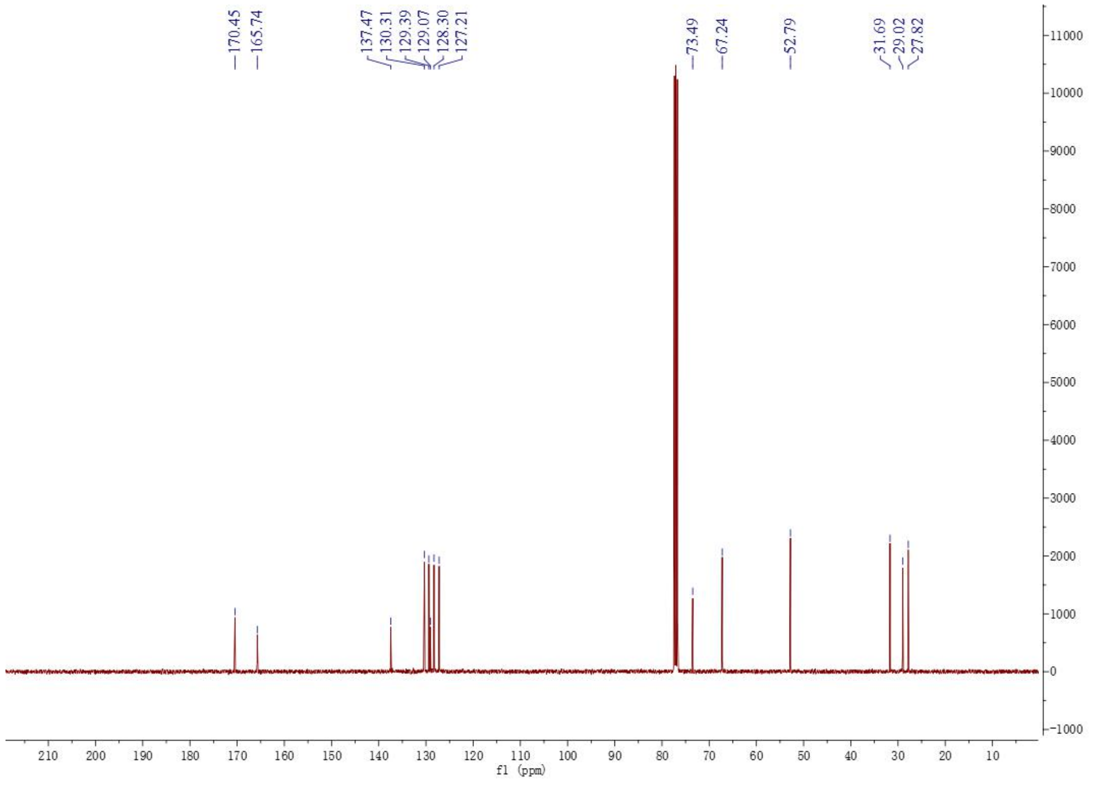

Figure S68. ^{13}C NMR spectrum of compound **4q** in $CDCl_3$.

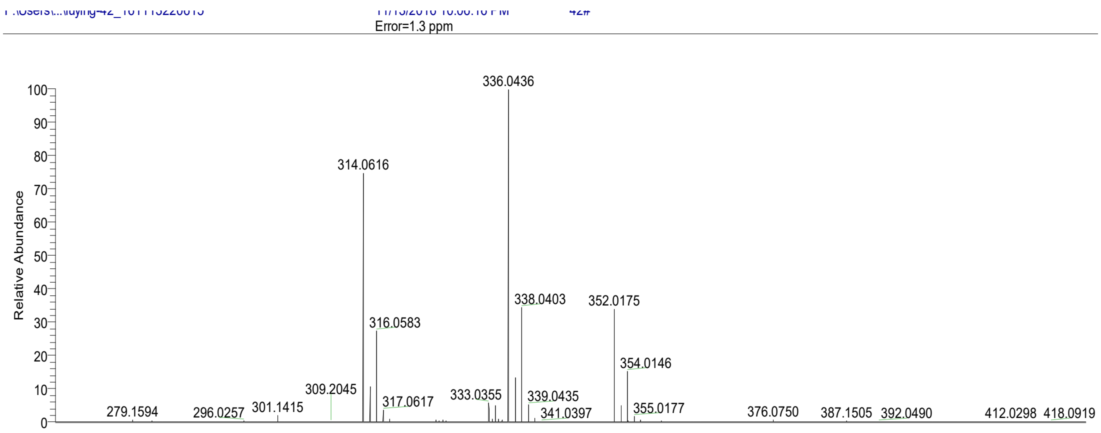

Figure S69. HMRS spectrum of compound **4q**.

Methyl (R)-4-(2,2-dichloroacetyl)-1-thia-4-azaspiro[4.5]decane-3-carboxylate (4r).

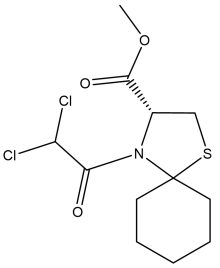

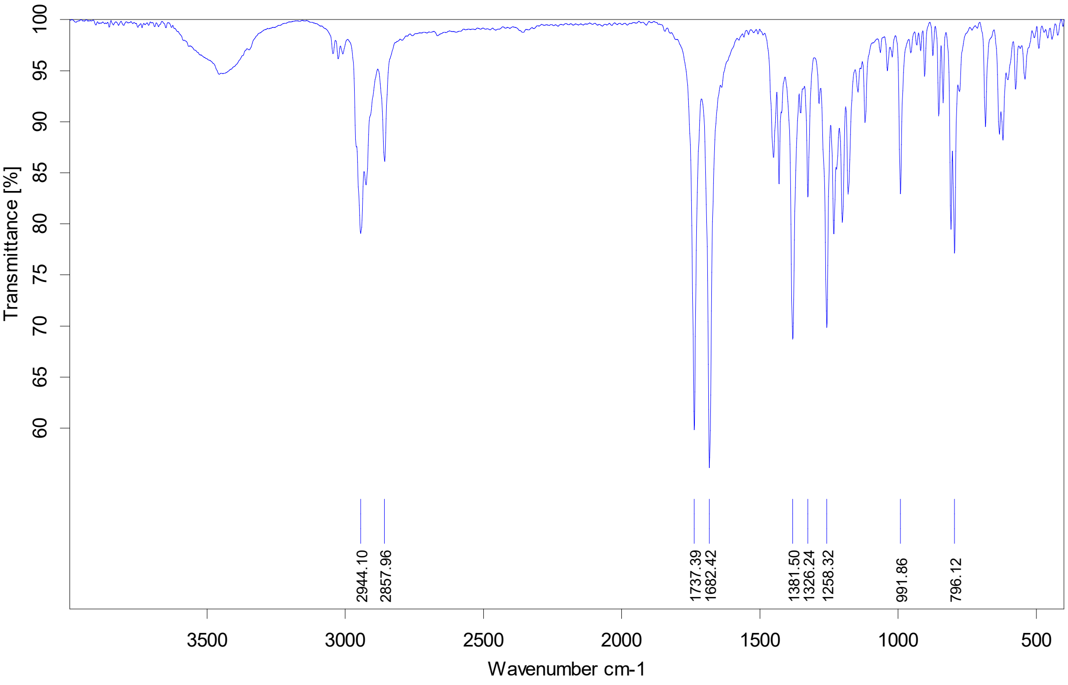

Figure S70. IR spectrum of compound **4r**.

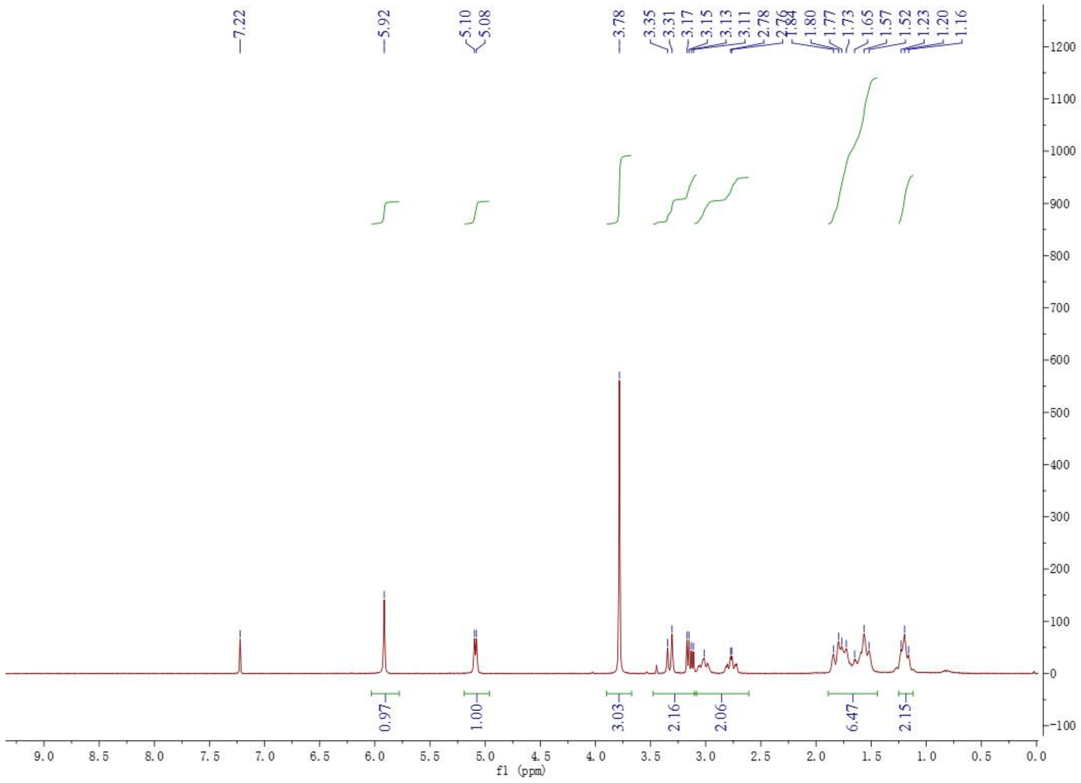

Figure S71. ^{1}H NMR spectrum of compound **4r**.

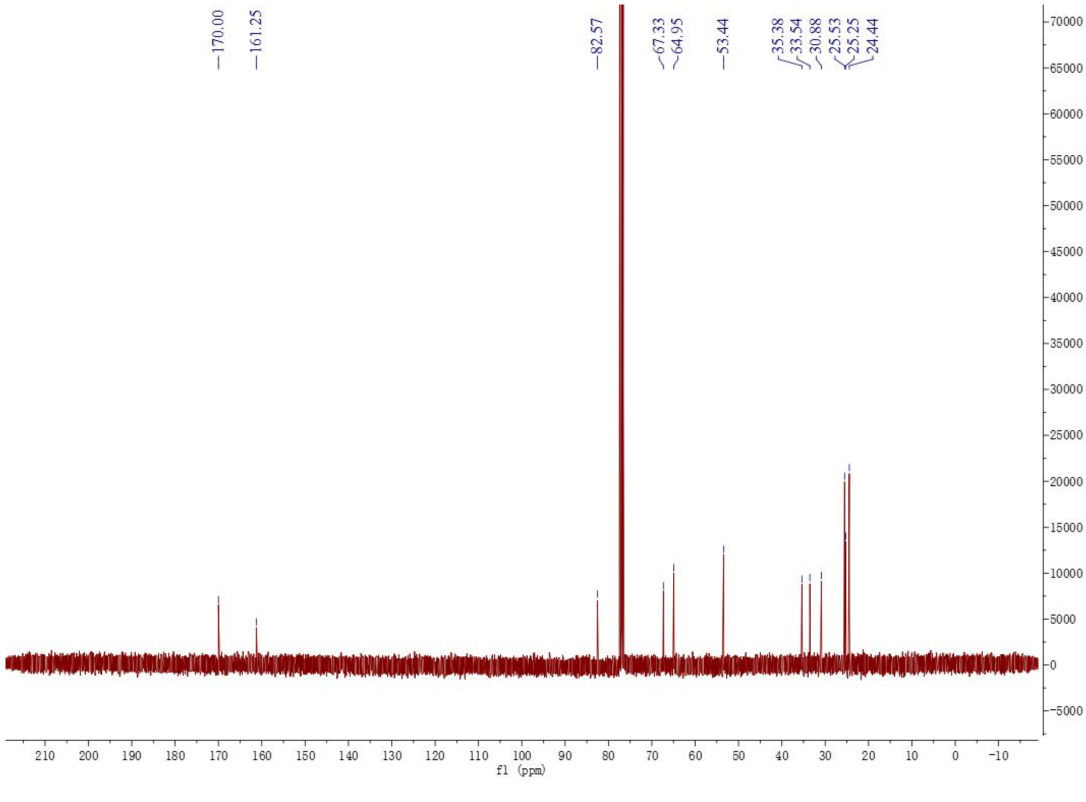

Figure S72. ^{13}C NMR spectrum of compound **4r** in $CDCl_3$.

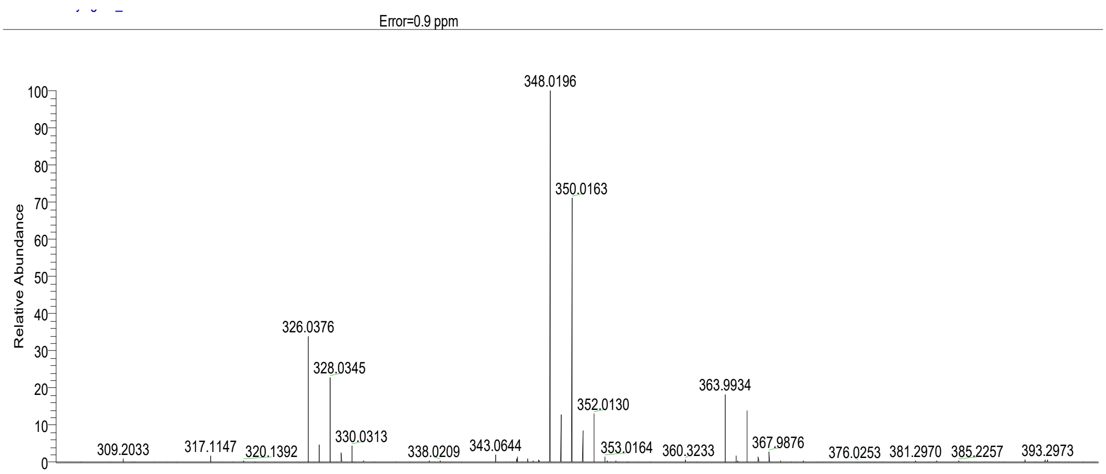

Figure S73. HMRS spectrum of compound **4r**.

Methyl (R)-3-(2,2-dichloroacetyl)-2,2-dimethylthiazolidine-4-carboxylate (4s).

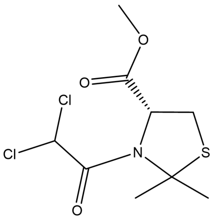

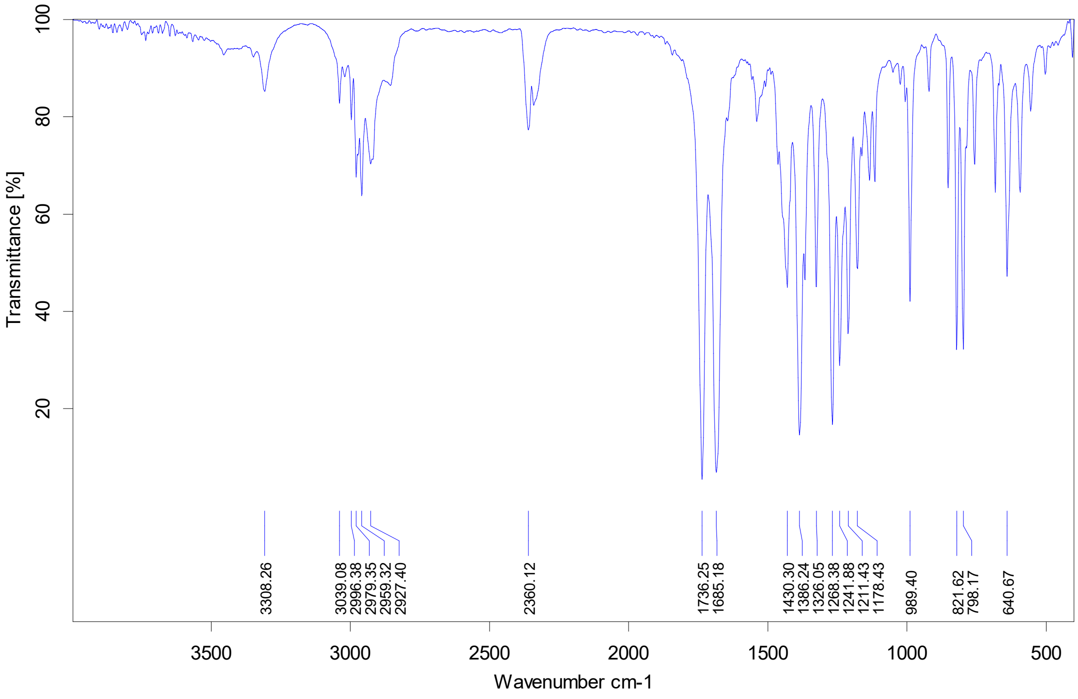

Figure S74. IR spectrum of compound **4s**.

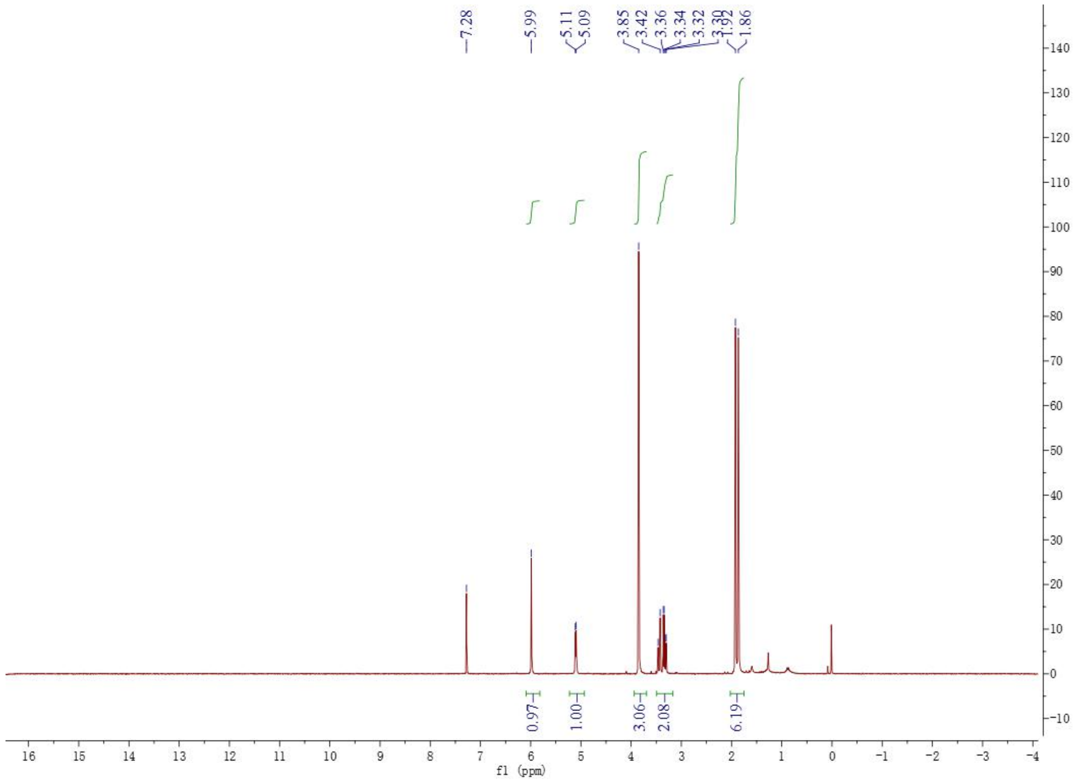

Figure S75. ^{1}H NMR spectrum of compound **4s**.

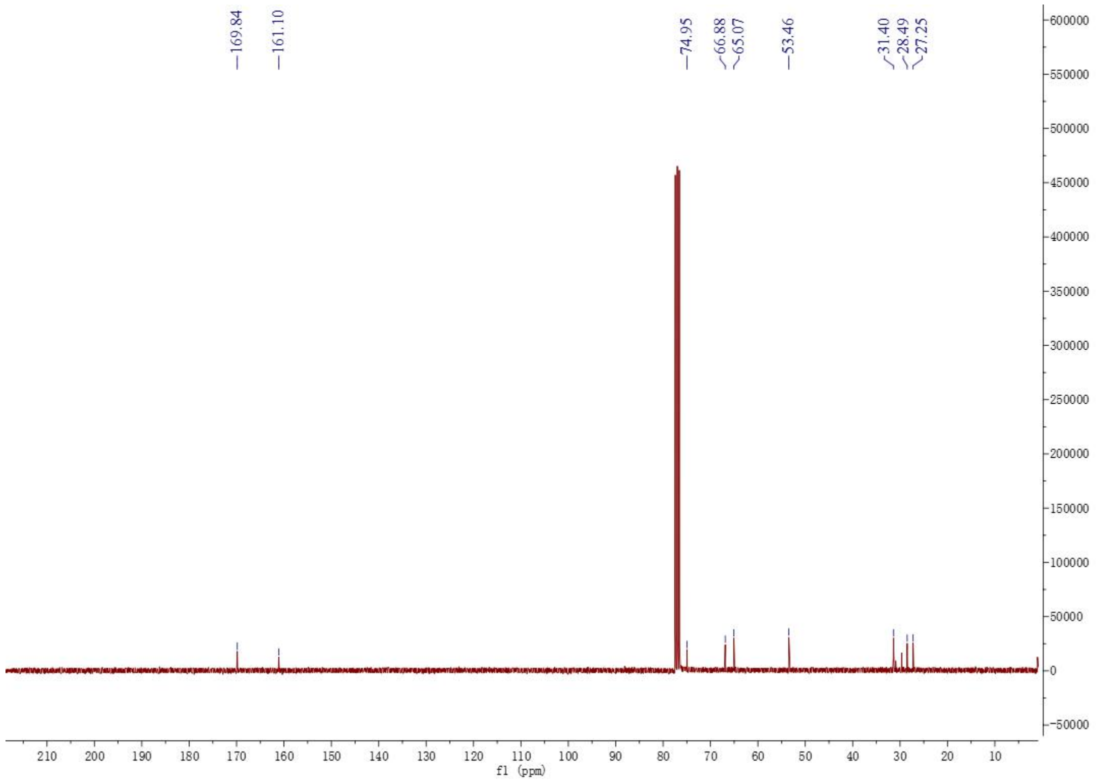

Figure S76. ^{13}C NMR spectrum of compound **4s** in $CDCl_3$.

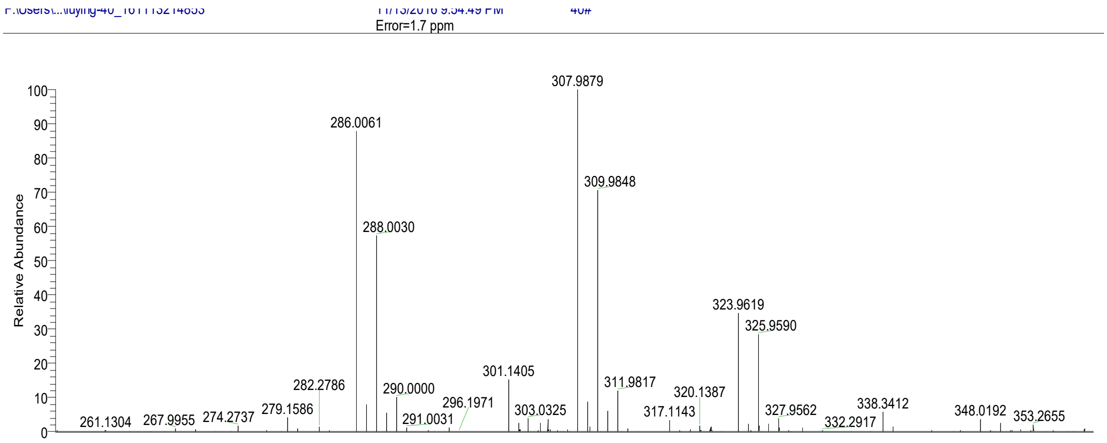

Figure S77. HMRS spectrum of compound **4s**.

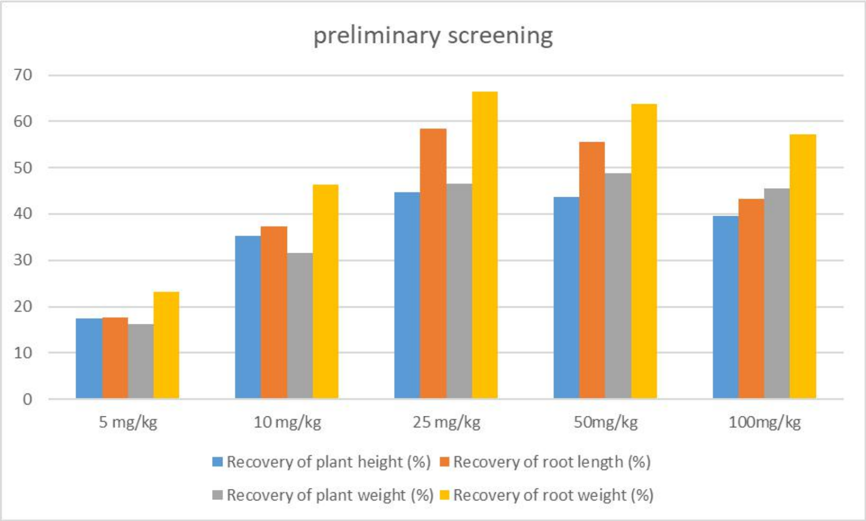

Figure S78. Preliminary screening

The physical data of the bioactivity test are presented in Table **S79-100**. Growth index were statistics of 21 maize plants. Each treatment was replicated three times in a completely randomized design.

Table S79. Maize seeds and soil were treated with water (control)

Growth index	Test 1	Test 2	Test 3	Recovery	Test 1	Test 2	Test 3
Mean value of plant height(cm)	18.02	18.58	17.58	Recovery of plant height(%)	100		
Mean value of root length(cm)	16.44	16.15	16.76	Recovery of root length(%)	100		
Sum of plant weight(g)	16.8711	17.4894	17.1067	Recovery of plant weight(%)	100		
Sum of root weight(g)	9.5541	9.1835	9.3929	Recovery of root weight(%)	100		

Table S80. Maize seeds were treated with water and soil was treated with chlorimuron-ethyl

Growth index	Test 1	Test 2	Test 3	Recovery	Test 1	Test 2	Test 3
Mean value of plant height(cm)	12.24	12.45	11.98	Recovery of plant height(%)	0		
Mean value of root length(cm)	11.48	11.82	11.13	Recovery of root length(%)	0		
Sum of plant weight(g)	11.6871	12.3418	11.9689	Recovery of plant weight(%)	0		
Sum of root weight(g)	5.6383	4.8282	5.1077	Recovery of root weight(%)	0		

Table S81. Maize seeds were treated with R-28725 and soil was treated with chlorimuron-ethyl

Growth index	Test 1	Test 2	Test 3	Recovery	Test 1	Test 2	Test 3
Mean value of plant height(cm)	17.23	17.82	16.73	Recovery of plant height(%)	86.31	87.54	84.80
Mean value of root length(cm)	15.71	15.44	15.94	Recovery of root length(%)	85.16	83.61	85.43
Sum of plant weight(g)	16.1743	16.9089	16.4455	Recovery of plant weight(%)	86.56	88.72	87.13
Sum of root weight(g)	8.9929	8.3868	8.6843	Recovery of root weight(%)	85.67	81.71	83.46

Table S82. Maize seeds were treated with **4a** and soil was treated with chlorimuron-ethyl

Growth index	Test 1	Test 2	Test 3	Recovery	Test 1	Test 2	Test 3
Mean value of plant height(cm)	14.65	15.07	14.46	Recovery of plant height(%)	41.79	42.71	44.21
Mean value of root length(cm)	14.41	14.49	14.56	Recovery of root length(%)	59.01	61.71	60.84
Sum of plant weight(g)	14.0079	14.7168	14.3825	Recovery of plant weight(%)	44.77	46.14	46.98
Sum of root weight(g)	8.2309	7.8025	8.1003	Recovery of root weight(%)	66.21	68.29	69.84

Table S83. Maize seeds were treated with **4b** and soil was treated with chlorimuron-ethyl

Growth index	Test 1	Test 2	Test 3	Recovery	Test 1	Test 2	Test 3
Mean value of plant height(cm)	15.64	16.18	15.35	Recovery of plant height(%)	58.78	60.93	60.16
Mean value of root length(cm)	13.71	13.85	13.64	Recovery of root length(%)	44.96	46.98	44.64
Sum of plant weight(g)	14.1246	14.7091	14.4589	Recovery of plant weight(%)	47.02	45.99	48.46
Sum of root weight(g)	8.3385	7.8211	8.0113	Recovery of root weight(%)	68.96	68.72	67.76

Table S84. Maize seeds were treated with **4c** and soil was treated with chlorimuron-ethyl

Growth index	Test 1	Test 2	Test 3	Recovery	Test 1	Test 2	Test 3
Mean value of plant height(cm)	16.87	17.43	16.44	Recovery of plant height(%)	80.15	81.22	79.61
Mean value of root length(cm)	15.81	15.58	15.95	Recovery of root length(%)	87.19	86.73	85.51
Sum of plant weight(g)	16.5148	17.2825	16.8348	Recovery of plant weight(%)	93.13	95.98	94.71
Sum of root weight(g)	8.9759	8.6437	8.7294	Recovery of root weight(%)	85.23	87.61	84.52

Table S85. Maize seeds were treated with **4d** and soil was treated with chlorimuron-ethyl

Growth index	Test 1	Test 2	Test 3	Recovery	Test 1	Test 2	Test 3
Mean value of plant height(cm)	16.23	16.59	15.74	Recovery of plant height(%)	69.13	67.56	67.21
Mean value of root length(cm)	13.61	13.66	13.41	Recovery of root length(%)	42.89	42.55	40.47
Sum of plant weight(g)	15.7475	16.3179	16.0375	Recovery of plant weight(%)	78.33	77.24	79.19
Sum of root weight(g)	7.3267	6.6473	6.8666	Recovery of root weight(%)	43.12	41.77	41.05

Table S86. Maize seeds were treated with **4e** and soil was treated with chlorimuron-ethyl

Growth index	Test 1	Test 2	Test 3	Recovery	Test 1	Test 2	Test 3
Mean value of plant height(cm)	17.40	17.86	16.99	Recovery of plant height(%)	89.32	88.34	89.57
Mean value of root length(cm)	16.00	15.73	16.32	Recovery of root length(%)	91.17	90.33	92.12
Sum of plant weight(g)	16.8652	17.3732	17.0613	Recovery of plant weight(%)	99.89	97.74	99.12
Sum of root weight(g)	9.3037	8.8583	9.1772	Recovery of root weight(%)	93.61	92.53	94.97

Table S87. Maize seeds were treated with **4f** and soil was treated with chlorimuron-ethyl

Growth index	Test 1	Test 2	Test 3	Recovery	Test 1	Test 2	Test 3
Mean value of plant height(cm)	15.82	16.27	15.44	Recovery of plant height(%)	61.99	62.28	61.87
Mean value of root length(cm)	14.65	14.61	14.74	Recovery of root length(%)	63.79	64.44	63.99
Sum of plant weight(g)	15.5161	16.2066	15.6835	Recovery of plant weight(%)	73.86	75.08	72.30
Sum of root weight(g)	7.6398	7.0984	7.3121	Recovery of root weight(%)	51.11	52.12	51.44

Table S88. Maize seeds were treated with **4g** and soil was treated with chlorimuron-ethyl

Growth index	Test 1	Test 2	Test 3	Recovery	Test 1	Test 2	Test 3
Mean value of plant height(cm)	17.26	17.70	16.74	Recovery of plant height(%)	86.91	85.69	84.99
Mean value of root length(cm)	15.87	15.65	15.97	Recovery of root length(%)	88.43	88.29	85.88
Sum of plant weight(g)	16.1868	16.7568	16.2999	Recovery of plant weight(%)	86.80	85.77	84.30
Sum of root weight(g)	8.8989	8.4232	8.7230	Recovery of root weight(%)	83.27	82.54	84.37

Table S89. Maize seeds were treated with **4h** and soil was treated with chlorimuron-ethyl

Growth index	Test 1	Test 2	Test 3	Recovery	Test 1	Test 2	Test 3
Mean value of plant height(cm)	15.58	15.93	15.15	Recovery of plant height(%)	57.87	56.72	56.64
Mean value of root length(cm)	13.76	13.90	13.75	Recovery of root length(%)	45.98	48.03	46.47
Sum of plant weight(g)	13.8622	14.4265	14.0924	Recovery of plant weight(%)	41.96	40.50	41.33
Sum of root weight(g)	7.9549	7.4588	7.6637	Recovery of root weight(%)	59.16	60.40	59.65

Table S90. Maize seeds were treated with **4i** and soil was treated with chlorimuron-ethyl

Growth index	Test 1	Test 2	Test 3	Recovery	Test 1	Test 2	Test 3
Mean value of plant height(cm)	14.85	15.36	14.46	Recovery of plant height(%)	45.12	47.46	44.35
Mean value of root length(cm)	14.27	14.21	14.24	Recovery of root length(%)	56.23	55.30	55.26
Sum of plant weight(g)	14.5551	15.0594	14.7258	Recovery of plant weight(%)	55.32	52.79	53.66
Sum of root weight(g)	7.8725	7.3601	7.5717	Recovery of root weight(%)	57.06	58.13	57.50

Table S91. Maize seeds were treated with **4j** and soil was treated with chlorimuron-ethyl

Growth index	Test 1	Test 2	Test 3	Recovery	Test 1	Test 2	Test 3
Mean value of plant height(cm)	15.64	15.91	15.17	Recovery of plant height(%)	58.82	56.41	57.01
Mean value of root length(cm)	14.87	14.72	14.77	Recovery of root length(%)	68.41	66.83	64.59
Sum of plant weight(g)	15.3002	16.0076	15.6951	Recovery of plant weight(%)	69.70	71.21	72.53
Sum of root weight(g)	8.3525	7.7647	7.9373	Recovery of root weight(%)	69.31	67.42	66.03

Table S92. Maize seeds were treated with **4k** and soil was treated with chlorimuron-ethyl

Growth index	Test 1	Test 2	Test 3	Recovery	Test 1	Test 2	Test 3
Mean value of plant height(cm)	14.69	15.03	14.28	Recovery of plant height(%)	42.38	42.01	41.06
Mean value of root length(cm)	13.79	13.82	13.73	Recovery of root length(%)	46.51	46.11	46.10
Sum of plant weight(g)	14.6309	15.2235	14.7797	Recovery of plant weight(%)	56.79	55.98	54.71
Sum of root weight(g)	7.7922	7.2284	7.4759	Recovery of root weight(%)	55.01	55.11	55.26

Table S93. Maize seeds were treated with **4l** and soil was treated with chlorimuron-ethyl

Growth index	Test 1	Test 2	Test 3	Recovery	Test 1	Test 2	Test 3
Mean value of plant height(cm)	13.47	13.70	13.11	Recovery of plant height(%)	0.2138	0.2033	0.2008
Mean value of root length(cm)	12.25	12.47	11.96	Recovery of root length(%)	0.1544	0.1501	0.1481
Sum of plant weight(g)	12.4218	13.0892	12.8137	Recovery of plant weight(%)	0.1417	0.1452	0.1644
Sum of root weight(g)	6.5783	5.9315	6.2423	Recovery of root weight(%)	0.2401	0.2533	0.2648

Table S94. Maize seeds were treated with **4m** and soil was treated with chlorimuron-ethyl

Growth index	Test 1	Test 2	Test 3	Recovery	Test 1	Test 2	Test 3
Mean value of plant height(cm)	15.10	15.41	14.73	Recovery of plant height(%)	49.48	48.36	49.12
Mean value of root length(cm)	14.79	14.62	14.89	Recovery of root length(%)	66.71	64.73	66.66
Sum of plant weight(g)	14.1924	14.7710	14.5021	Recovery of plant weight(%)	48.33	47.19	49.31
Sum of root weight(g)	7.5263	6.9099	7.1428	Recovery of root weight(%)	48.21	47.80	47.49

Table S95. Maize seeds were treated with **4n** and soil was treated with chlorimuron-ethyl

Growth index	Test 1	Test 2	Test 3	Recovery	Test 1	Test 2	Test 3
Mean value of plant height(cm)	13.74	13.99	13.33	Recovery of plant height(%)	25.93	25.05	24.09
Mean value of root length(cm)	12.98	13.07	12.73	Recovery of root length(%)	30.16	28.95	28.37
Sum of plant weight(g)	12.5407	13.2512	12.8363	Recovery of plant weight(%)	16.47	17.67	16.88
Sum of root weight(g)	6.6506	5.9092	6.1428	Recovery of root weight(%)	25.85	24.82	24.16

Table S96. Maize seeds were treated with **4o** and soil was treated with chlorimuron-ethyl

Growth index	Test 1	Test 2	Test 3	Recovery	Test 1	Test 2	Test 3
Mean value of plant height(cm)	14.30	14.52	13.83	Recovery of plant height(%)	35.60	33.71	33.07
Mean value of root length(cm)	13.36	13.47	13.32	Recovery of root length(%)	37.92	38.18	38.80
Sum of plant weight(g)	13.7411	14.4301	13.9488	Recovery of plant weight(%)	39.62	40.57	38.54
Sum of root weight(g)	7.8901	7.2798	7.4753	Recovery of root weight(%)	57.51	56.29	55.25

Table S97. Maize seeds were treated with **4p** and soil was treated with chlorimuron-ethyl

Growth index	Test 1	Test 2	Test 3	Recovery	Test 1	Test 2	Test 3
Mean value of plant height(cm)	16.65	17.16	16.16	Recovery of plant height(%)	76.38	76.93	74.70
Mean value of root length(cm)	14.60	14.46	14.48	Recovery of root length(%)	62.93	60.89	59.45
Sum of plant weight(g)	15.1935	15.8925	15.5738	Recovery of plant weight(%)	67.64	68.98	70.16
Sum of root weight(g)	7.8652	7.1809	7.4992	Recovery of root weight(%)	56.87	54.02	55.81

Table S98. Maize seeds were treated with **4q** and soil was treated with chlorimuron-ethyl

Growth index	Test 1	Test 2	Test 3	Recovery	Test 1	Test 2	Test 3
Mean value of plant height(cm)	13.57	13.85	13.31	Recovery of plant height(%)	23.01	22.79	23.80
Mean value of root length(cm)	12.20	12.44	11.97	Recovery of root length(%)	14.41	14.33	14.92
Sum of plant weight(g)	12.5968	13.3303	13.0156	Recovery of plant weight(%)	17.55	19.20	20.37
Sum of root weight(g)	6.6253	5.9054	6.2141	Recovery of root weight(%)	25.21	24.73	25.82

Table S99. Maize seeds were treated with **4r** and soil was treated with chlorimuron-ethyl

Growth index	Test 1	Test 2	Test 3	Recovery	Test 1	Test 2	Test 3
Mean value of plant height(cm)	15.66	15.93	15.18	Recovery of plant height(%)	59.24	56.85	57.12
Mean value of root length(cm)	13.51	13.52	13.28	Recovery of root length(%)	40.97	39.33	38.09
Sum of plant weight(g)	13.2760	13.9557	13.6331	Recovery of plant weight(%)	30.65	31.35	32.39
Sum of root weight(g)	7.5083	6.7940	7.0845	Recovery of root weight(%)	47.76	45.14	46.13

Table S100. Maize seeds were treated with **4s** and soil was treated with chlorimuron-ethyl

Growth index	Test 1	Test 2	Test 3	Recovery	Test 1	Test 2	Test 3
Mean value of plant height(cm)	14.17	14.38	13.93	Recovery of plant height(%)	33.36	31.51	34.80
Mean value of root length(cm)	13.36	13.38	13.10	Recovery of root length(%)	37.86	36.03	35.02
Sum of plant weight(g)	14.0624	14.7748	14.2388	Recovery of plant weight(%)	45.82	47.26	44.18
Sum of root weight(g)	7.0962	6.5134	6.7254	Recovery of root weight(%)	37.23	38.69	37.75
